# Correction: Advanced Pediatric Emergency Airway Management: A Multimodality Curriculum Addressing a Rare but Critical Procedure

**DOI:** 10.15766/mep_2374-8265.11113

**Published:** 2021-01-12

## Correction

In the publication “Advanced Pediatric Emergency Airway Management: A Multimodality Curriculum Addressing a Rare but Critical Procedure,” in the Methods section, on the equipment list for Station 2, an abbreviation in one of the bulleted items was incorrectly explained. The bulleted item should read:

• Cognitive aids (e.g., Broselow tape, Pediatric Advanced Life Support [PALS] cards, PediStat app, e-Broselow app)
