# Supplementary material for: Advanced Pediatric Emergency Airway Management: A Multimodality Curriculum Addressing a Rare but Critical Procedure
Source: MedEdPORTAL. 2020 Sep 4;16:10962. doi: 10.15766/mep_2374-8265.10962 (PMC7473185; doi:10.15766/mep_2374-8265.10962)
Supplement: Supplementary file 1 — Course Syllabus.docxStation 1 Didactic Videos.pptxStation 2 Needle Cricothyrotomy Cognitive Aid.pptxIntubation Teaching Feedback Rubrics.docxStation 3 Simulation.docxStation 4 Simulation.docxCurriculum Evaluation.docx [file mep_2374-8265.10962-s001.zip › A. Course Syllabus.docx]

***Appendix #A - Pediatric Airway Course Syllabus:***

A brief note to future curriculum leaders and facilitators:

*“Please accept this course syllabus as an outline for specifics about each learning station. It starts with a sample schedule, followed by detailed learning goals, objectives, and materials needed at each station. Finally, key teaching points are included in this document for stations 1 and 2 and in the SIM appendices for stations 3 and 4”*

**Logistics with a Sample Times:**

8:00 – 8:15:

Intro, logistics, the day’s goals/objectives

8:15 – 8:55:

*Station 1: Reviewing Key Pediatric Airway Management Considerations*

9:00 – 9:35:

*Station 2: Choosing the Right Equipment Quickly & Needle Cricothyroidotomy*

9:40 – 10:15:

*Station 3: SIMULATION #1 – Impending Respiratory Failure in the Pediatric Patient*

10:20 – 10:55:

*Station 4: SIMULATION #2 – The Difficult Pediatric Airway*

10:55 - 11:00:

Post-Participation Evaluations

***Participants will be assigned to their rotation groups by instructors as they arrive for the day. The goal is to have a good mix of care team members (RN, MD, Tech, Respiratory Therapy, or level of training (PGY 1, 2, 3, etc.…) for the SIMs and to have even numbers in each group***

**Intro:**

1. Introduction of faculty
2. Teaching, retaining skills around rare procedures is challenging but necessary! We are here to address this learning gap as it pertains to Pediatric Emergency Airway Management.
3. Review Learning Goals for the day
4. Introduce the 4 stations, preview each stations’ learning objectives
5. Review the learner rotation plan
6. Orient to location of bathrooms and other possible needs of the group

**Station 1:**

*Reviewing Key Pediatric Airway Management Considerations*

*Educational Modality: Brief interactive didactic and case discussions with integrated clinically obtained real intubation videos*

*Learning Goal -* **Appreciate the key aspects of pediatric anatomy and physiology, systems issues and technologies that lend to the successful assessment and management of the pediatric airway.**

*Learning Objectives -*

After completing this station**,** participants will be able to:

- - 1. Review and apply key pediatric anatomic and physiologic features that promote successful assessment and management of the pediatric airway
    2. Compare and contrast the pros and cons to different laryngoscopy techniques including: “curved” versus “straight” blades, “progressive visualization of landmarks,” versus “bury the blade technique,” “the right sided approach” versus “the midline approach”
    3. Observe the importance of oropharyngeal suction and external laryngeal manipulation in optimizing laryngoscopy
    4. Discuss how team-based direct laryngoscopy with a video laryngoscope facilitates immediate direct procedural feedback which may increase intubation success
    5. Describe the advantage of team-based intubation to confirm successful endotracheal intubation

**Materials**

- PowerPoint (appendix B)
- AV connection
- Laser pointer

**Station 2:**

*Choosing the Right Equipment Quickly & Needle Cricothyrotomy*

*Educational Modality: Direct observation and feedback by a faculty member using airway trainers*

*Learning Goal* **- Deliberately practice and receive coaching on direct and video laryngoscopy techniques and failed airway management skill sets such as Laryngeal Mask Airway (LMA) and Needle Cricothyrotomy.**

*Learning Objectives -*

After completing this station, participants will be able to:

1. Improve upon and recognize the importance of preparation time (chronometry) in obtaining proper intubation equipment
2. Practicing using established formulas and/or cognitive aids to select proper intubation equipment sizes.
3. Place an endotracheal tube in an infant and child airway trainer mannequin using both direct and video laryngoscopy.
4. Place an LMA in an infant and a child airway trainer.
5. List indications for surgical airways in children.
6. Place a needle cricothyrotomy on a needle cricothyrotomy airway trainer
7. Use a cognitive aid to facilitate procedural success with needle cricothyrotomy
8. Demonstrate slow ventilation technique through a needle cricothyrotomy on an airway trainer

**Materials**

- High fidelity pediatric airway trainers (e.g. Syndaver Child and Infant)
- Laryngoscope Handles
- Laryngoscope Blades – Whis-hipple 00, Miller 0, 1, 2; Macintosh 1, 2, 3
  - Video Laryngoscope blades and handles
- Cuffed endotracheal tubes sizes – 3.0, 3.5, 4.0, 4.5, 5.0, 5.5, 6.0, 6.5
- Laryngeal Mask Airways – Sizes 1, 1.5, 2, 2.5, 3, 4, 5
- Cognitive Aids (e.g. Broselow tape, PALS cards, PediStat app, e-Broselow app)
- Stopwatch
- Needle Cricothyrotomy Trainer and procedure equipment
  - 14g IV
  - 3mL syringes
  - Top of a 7.0 Endotracheal adapter
  - Pediatric self-inflatable bag valve mask
  - Needle Cricothyrotomy Cognitive Aid (appendix C)

**Logistics –**

*Activity #1 - Choosing the Right Equipment Quickly*

- Prep the learner – We just got a patch about a very sick 3-month-old. Have the learner sort through a pile of available equipment to choose the proper equipment and perform the intubation.
- Time the drill and critique their accuracy with respect to proper size of equipment selected.
- Repeat after providing and reviewing use of a cognitive aid such as the Pedistat smartphone application or a PALS card.
- Repeat this exercise for different peds ages as time allows
- Thereafter practice laryngoscopy, intubation and LMA placement on multiple sized airway trainers.
- Facilitators may consider using one of the two referenced intubation competency checklists to help standardize their teaching scripts for the learners (Appendix D).^1,2^

*Activity #2 - Needle Cricothyrotomy*

- Briefly discuss indications for needle cricothyrotomy in children under age 5.
- Demonstrate and then have learners practice the steps to place a needle cricothyrotomy and connect equipment to oxygenate the patient.
- Ensure long exhalation time and slow but forceful inhalation given the high resistance circuit established.
- [OPTIONAL] Depending on your faculty compliment, for this station, you may choose to set up this learning activity as an independent learning opportunity.
  - We suggest having learners read the following module:
    - Indications –
    - <http://www.emcurious.com/blog-1/2014/11/7/surgical-airway-part-2>
  - We then suggest having learners watch the following video for Needle Cricothyrotomy
    - Set up and Placement
    - <https://www.youtube.com/watch?v=XMtPg6wkdKI>
  - Of note, reviewing this material may be beneficial for faculty teaching this activity as this procedure is exceedingly rare.

**Station #3**

*SIMULATION #1 – Impending Respiratory Failure in the Pediatric Patient*

*Educational Modality: Simulation-based scenario followed by debrief*

*Learning Goal* **- Recognize and manage respiratory failure in a pediatric patient.**

*Learning Objectives –*

After completing this station**,** participants will be able to:

1. Recognize at least two signs and symptoms of impending respiratory failure in the pediatric patient
2. Verbalize and implement a pediatric rapid sequence intubation plan considering the risks and benefits of different pre-medications, induction agents and neuromuscular blocking agents
3. Use a peri-intubation checklist
4. To optimize first pass success, perform “team-based intubation” using direct laryngoscopy technique with a video laryngoscope so the team leader / airway preceptor can provide immediate procedural feedback as indicated.
5. Experience the advantage of team-based intubation to confirm proper endotracheal tube placement

**Materials**

- Simulation scenario instructor guide (appendix # E)
- SIM Baby with IV in place, no additional moulage
- Blades, Wis-Hipple 00, Miller 0, 1, 2, Macintosh 2, 3
- Cuffed endotracheal tubes of variable sizes
- **Video Laryngoscope such as Glidescope or CMAC system*
- Self-inflating bag and masks sizes infant, pediatric/child, adult
- Cognitive Aids (e.g. Broselow tape, PALS cards, PediStat app, e-Broselow app)
- Airway adjuncts – Nasopharyngeal airways, Oropharyngeal airways of varying sizes
- Airway Rescue Devices – Supraglottic airway such as an LMA
- Code and RSI medication tray
  - Dextrose as D10, D25 and D50
  - Bag of NS
  - Ativan
  - Midazolam
  - Phosphenytoin
  - Keppra
  - Propofol
  - Etomidate
  - Fentanyl
  - Morphine
  - Atropine
  - Rocuronium
  - Vecuronium
  - Succinylcholine
  - Epinephrine

The SIM in Brief –

- Senior MD precepting a more novice airway manager using “team-based” laryngoscopy:
  - The novice airway manager performs traditional Direct Laryngoscopy – looking into the mouth and calling out key anatomic structures as they advance towards the glottis.
  - The more senior team leader / airway preceptor can articulate as much as needed based on what they see on the video screen using short and direct feedback.
    - E.g. Pull back, you are too deep, that is the esophagus, pull the tube!
- Scene - Status Epilepticus with Respiratory Failure.
- Debrief focuses on how/why this feels different from their adult airway management?
  - What was similar and what was different to your process with the child?
  - Was the Video Laryngoscope helpful in your process as the preceptor?
  - Was the Video Laryngoscope helpful to confirm endotracheal tube placement?

The SIM’s Key Learning Points are noted in the branch point and anticipated mistakes section of the Simulation Appendix.

**Station 4**

*SIMULATION #2 – The Difficult Pediatric Airway*

*Educational Modality: Simulation-based scenario followed by debrief*

***Learning Goal* -** Predict, plan for and manage the difficult pediatric airway.

*Learning Objectives –*

After completing this station, participants will be able:

1. List clinical signs, symptoms and historical factors that should raise concern for a difficult pediatric airway
2. Activate back-up plans under the control of the immediate provider as well as within a hospital or regional health care system
3. Apply a difficult airway algorithm that includes bag mask ventilation, use of airway adjunctive equipment and early consideration of a supraglottic airway device

**Materials**

- Simulation scenario instructor guide (appendix # F)
- SIM Baby with IV in place, gtube, no moulage
- Blades, Wis-Hipple 00, Miller 0, 1, 2, Macintosh 2, 3
- Cuffed endotracheal tubes of variable sizes
- **Video Laryngoscope such as Glidescope or CMAC system*
- Self-inflating bag and masks sizes infant, pediatric/child, adult
- Cognitive Aids (e.g. Broselow tape, PALS cards, PediStat app, e-broselow app)
- Airway adjuncts – Nasopharyngeal airways, Oropharyngeal airways
- Airway Rescue Devices – Supraglottic airway (e.g. laryngeal mask airway - LMA)
- Supplies for needle cricothyrotomy
  - Self-Inflating Ventilation Bag
  - 7.0 ETT Connector
  - 3mL Syringe Barrel (plunger removed)
  - 14g IV Catheter loaded on a small saline filled syringe
- Code and RSI medication tray
  - Dextrose as D10, D25 and D50
  - Bag of NS
  - Ativan
  - Midazolam
  - Propofol
  - Etomidate
  - Atropine
  - Fentanyl
  - Morphine
  - Rocuronium
  - Vecuronium
  - Succinylcholine
  - Epinephrine
- Early consideration of supraglottic rescue devices
- Early activation of a backup plan – critical care transport teams, ENT/Anesthesia, etc.!

The SIM in Brief –

- Team leader is foot of bed. Airway manager is ***clued in*** on the fact that emphasis of this SIM is activating your ED’s difficult airway backup plan – so they will act that they cannot pass the tube!
- While the respiratory failure will be clear, the role of bagging and or non-invasive ventilation with airway adjuncts, obtaining and using your rescue devices and activating your backup plan / transport team early is the ultimate learning goal for this station.

Helpful Reference –

- <https://vimeo.com/34883844>
  - VERY good video for placing emphasis on ventilation skills over laryngoscopy!

**Key References:**

**Two Validated Teaching Rubrics / Checklists for Pediatric Intubation:**

1. Johnston L, Sawyer T, Nishisaki A, et al. Neonatal Intubation Competency Assessment Tool: Development and Validation. *Acad Pediatr*. 2019. doi:10.1016/j.acap.2018.07.008

2. Abu-Sultaneh S, Whitfill T, Rowan CM, et al. Improving Simulated Pediatric Airway Management in Community Emergency Departments Using a Collaborative Program With a Pediatric Academic Medical Center. *Respir Care*. April 2019:respcare.06750. doi:10.4187/respcare.06750

**References for the Key Learning Points Covered in all 4 Stations:**

1. Nagler, J, Balga, T, Goldman, MP. “Approach to Pediatric Emergency Airway Management Podcast.” Yale Emergency Medicine Apple Podcasts. Co-host and content developer. <https://podcasts.apple.com/us/podcast/yale-emergency-medicine-podcasts/id986369835?i=1000440671132>. 4/2019.
2. Nagler J, Nishisaki, A, Goldman MP, Johnston L, Scherzer D. Sawyer T, White ML, Auerbach M, Wolbrink TA. Pediatric Emergent Tracheal Intubation. Online video. OPENPediatrics. <https://www.openpediatrics.org/assets/video/pediatric-endotracheal-intubation.12/2019>
3. The Difficult Airway Course: Emergency and Walls, RM, et al. Manual of Emergency Airway Management, 2nd Ed, Lippincott Williams & Wilkins, Philadelphia 2004
4. Strayer, R. Emergency Ventilation in 11 Minutes. <https://vimeo.com/34883844> -

**We designed the Pediatric Airway Course with the following RRC Objectives in Mind:**

**Program Requirements for GME in Emergency Medicine – ACGME Home**

<https://www.acgme.org/Portals/0/PFAssets/ProgramRequirements/110_emergency_medicine_2017-07-01.pdf>

IV.A.5.a).(1).(j) identifying life-threatening conditions and the most likely diagnosis, synthesizing acquired patient data, and identifying how and when to access current medical information;

**IV.A.5.a).(2) Residents must be able to competently perform all medical, diagnostic and surgical procedures considered essential for the area of practice. Residents**

IV.A.5.a).(2).(a).(i) performing diagnostic and therapeutic procedures and emergency stabilization

IV.A.5.a).(2).(a).(ii) managing critically-ill and injured patients who present to the emergency department, prioritizing critical initial stabilization action, mobilizing hospital support services in the resuscitation of critically-ill or injured patients and reassessing after a stabilizing intervention;

IV.A.5.a).(2).(a).(iv) mobilizing and managing necessary personnel and other hospital resources to meet critical needs of multiple patients; and,

IV.A.5.a).(2).(a).(v) performing invasive procedures, monitoring unstable patients, and directing major resuscitations of all types on all age groups

IV.A.5.a).(2).(b) must perform indicated procedures on all appropriate patients, including those who are uncooperative, at the extremes of age, hemodynamically unstable and who have multiple co-morbidities, poorly defined anatomy, high risk for pain or procedural complications, or require sedation, take steps to avoid potential complications; and recognize the outcome and/or complications resulting from the procedures

IV.A.5.a).(2).(c) must demonstrate competence in performing the following key index procedures

IV.A.5.a).(2).(c).(vi) cricothyrotomy;

IV.A.5.a).(2).(c).(ix) intubations; (Outcome)

***IV.A.5.a).(2).(c).(ix).(a) Residents must perform airway management on all appropriate patients, including those who are uncooperative, at the extremes of age, hemodynamically unstable and who have multiple co-morbidities, poorly-defined anatomy, high risk for pain or procedural complications, or require sedation); take steps to avoid potential complications; and recognize the outcome and/or complications resulting from the procedures.

IV.A.5.a).(2).(c).(xi) pediatric medical resuscitation; (Outcome)

IV.A.5.a).(2).(c).(xii) pediatric trauma resuscitation

IV.A.5.a).(2).(c).(xvi) vascular access; and, (Outcome)

IV.A.5.a).(2).(c).(xvi).(a) Residents must successfully obtain vascular access in patients of all ages regardless of the clinical situation

**IV.A.5.d) Interpersonal and Communication Skills**

**Residents must demonstrate interpersonal and communication skills that result in the effective exchange of information and collaboration with patients, their families, and health professionals**

**IV.A.5.d).(3) work effectively as a member or leader of a health care team or other professional group**

IV.A.5.d).(7) lead patient care teams, ensuring effective communication and mutual respect among team members

**IV.A.5.f) Systems-based Practice Residents must demonstrate an awareness of and responsiveness to the larger context and system of health care, as well as the ability to call effectively on other resources in the system to provide optimal health care**

**IV.A.5.f).(1) work effectively in various health care delivery settings and systems relevant to their clinical specialty;**(Outcome)

**IV.A.5.f).(2) coordinate patient care within the health care system relevant to their clinical specialty**

IV.A.6.a).(1) four months of dedicated critical care experiences, including critical care of infants and children
