# Supplementary material for: Advanced Pediatric Emergency Airway Management: A Multimodality Curriculum Addressing a Rare but Critical Procedure
Source: MedEdPORTAL. 2020 Sep 4;16:10962. doi: 10.15766/mep_2374-8265.10962 (PMC7473185; doi:10.15766/mep_2374-8265.10962)
Supplement: Supplementary file 1 — Course Syllabus.docxStation 1 Didactic Videos.pptxStation 2 Needle Cricothyrotomy Cognitive Aid.pptxIntubation Teaching Feedback Rubrics.docxStation 3 Simulation.docxStation 4 Simulation.docxCurriculum Evaluation.docx [file mep_2374-8265.10962-s001.zip › B. Station 1 Didactic Videos.pptx]

## Slide 1
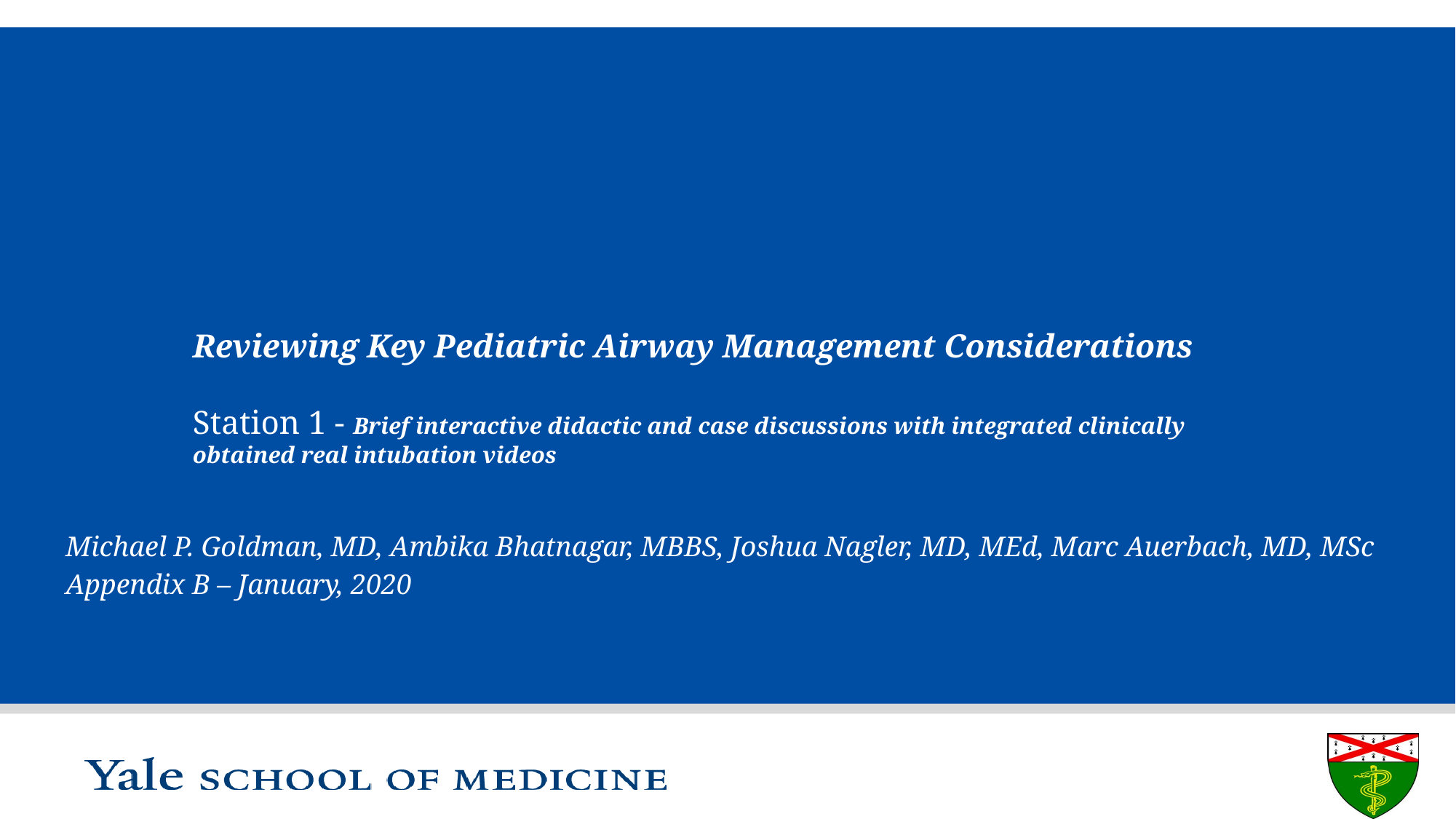

# Reviewing Key Pediatric Airway Management Considerations Station 1 - Brief interactive didactic and case discussions with integrated clinically obtained real intubation videos
Michael P. Goldman, MD, Ambika Bhatnagar, MBBS, Joshua Nagler, MD, MEd, Marc Auerbach, MD, MSc
Appendix B – January, 2020

## Slide 2
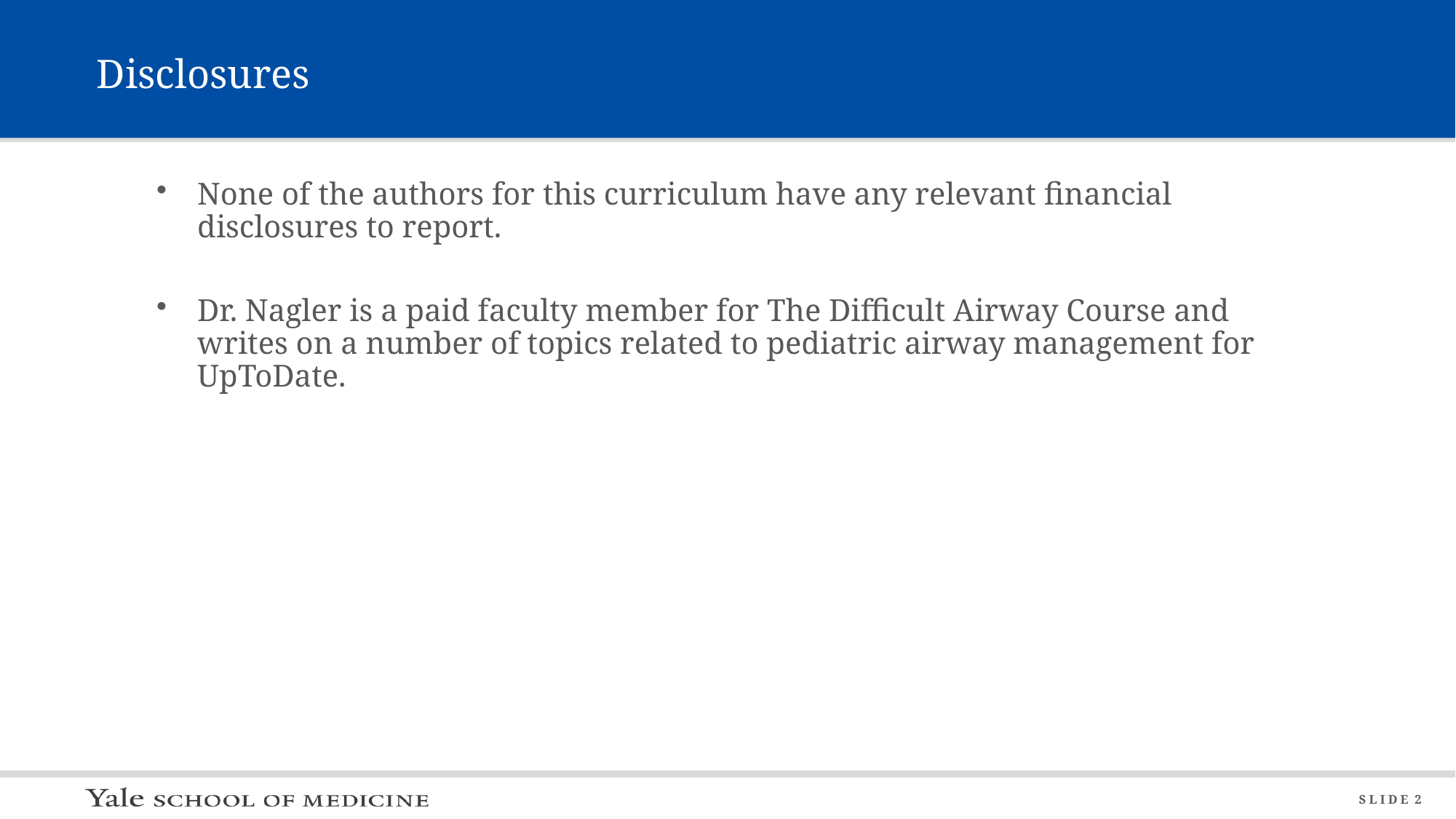

# Disclosures
None of the authors for this curriculum have any relevant financial disclosures to report.
Dr. Nagler is a paid faculty member for The Difficult Airway Course and writes on a number of topics related to pediatric airway management for UpToDate.

## Slide 3
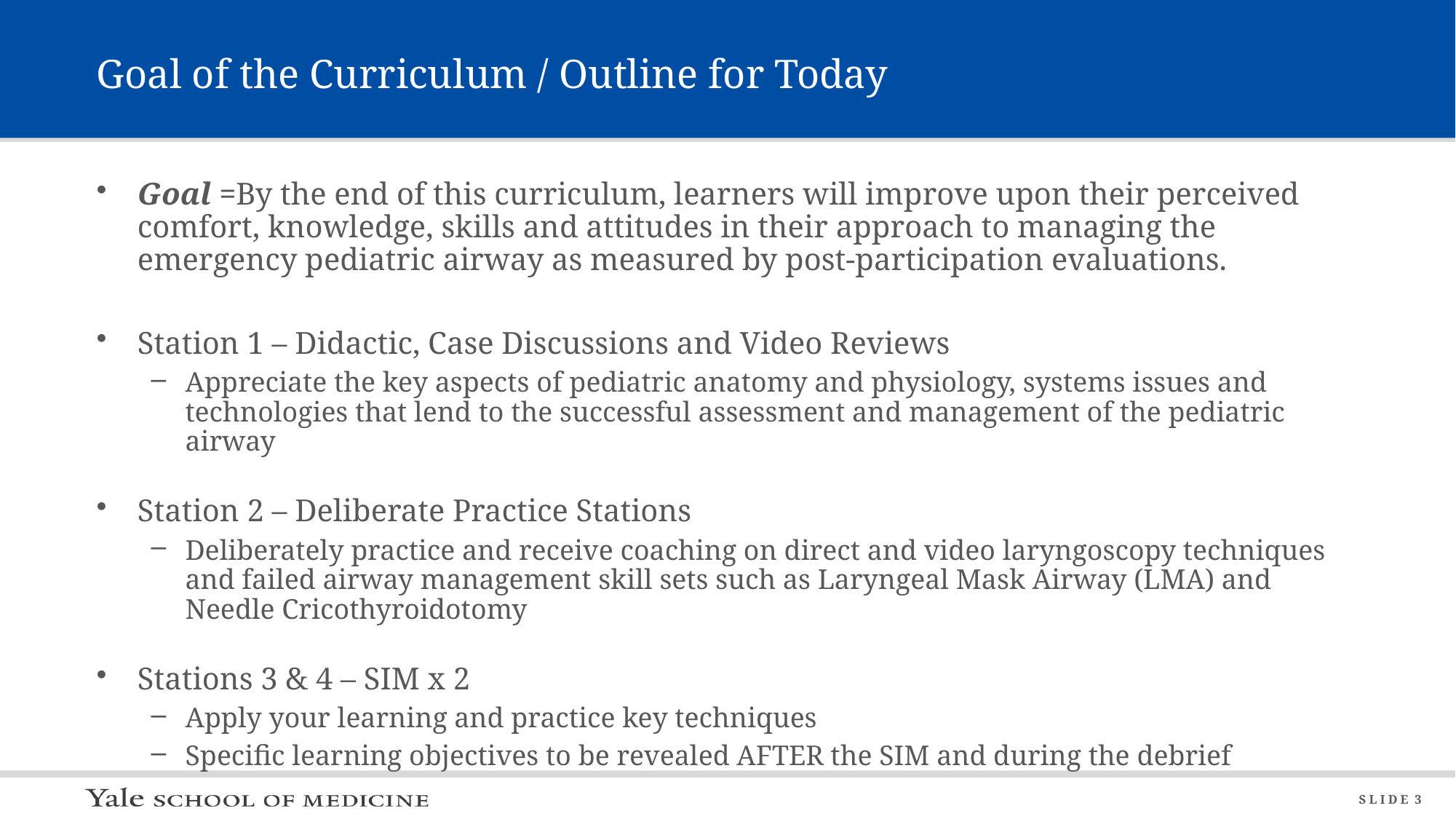

# Goal of the Curriculum / Outline for Today
Goal =By the end of this curriculum, learners will improve upon their perceived comfort, knowledge, skills and attitudes in their approach to managing the emergency pediatric airway as measured by post-participation evaluations.
Station 1 – Didactic, Case Discussions and Video Reviews
Appreciate the key aspects of pediatric anatomy and physiology, systems issues and technologies that lend to the successful assessment and management of the pediatric airway
Station 2 – Deliberate Practice Stations
Deliberately practice and receive coaching on direct and video laryngoscopy techniques and failed airway management skill sets such as Laryngeal Mask Airway (LMA) and Needle Cricothyroidotomy
Stations 3 & 4 – SIM x 2
Apply your learning and practice key techniques
Specific learning objectives to be revealed AFTER the SIM and during the debrief

## Slide 4
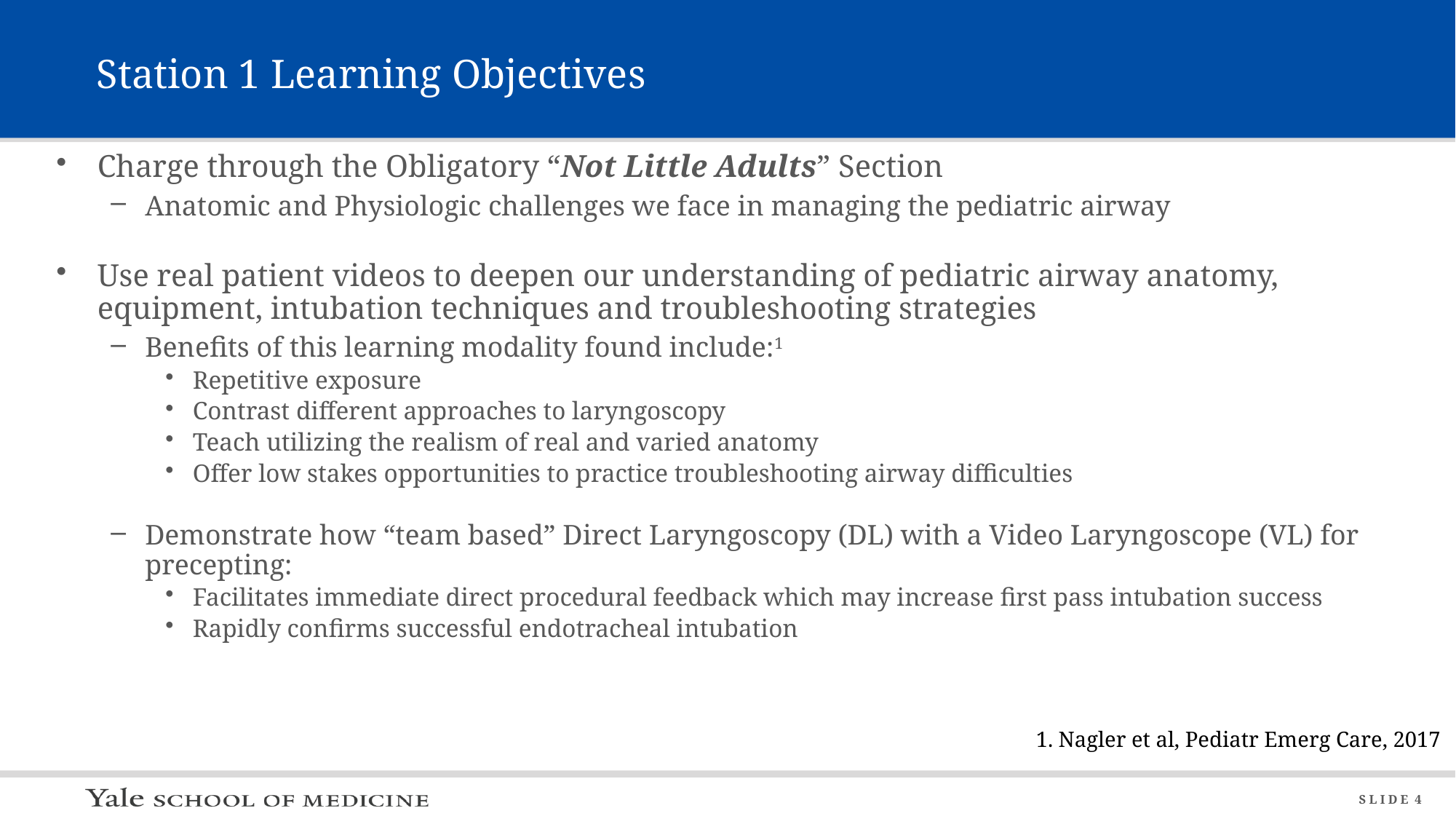

# Station 1 Learning Objectives
Charge through the Obligatory “Not Little Adults” Section
Anatomic and Physiologic challenges we face in managing the pediatric airway
Use real patient videos to deepen our understanding of pediatric airway anatomy, equipment, intubation techniques and troubleshooting strategies
Benefits of this learning modality found include:1
Repetitive exposure
Contrast different approaches to laryngoscopy
Teach utilizing the realism of real and varied anatomy
Offer low stakes opportunities to practice troubleshooting airway difficulties
Demonstrate how “team based” Direct Laryngoscopy (DL) with a Video Laryngoscope (VL) for precepting:
Facilitates immediate direct procedural feedback which may increase first pass intubation success
Rapidly confirms successful endotracheal intubation
1. Nagler et al, Pediatr Emerg Care, 2017

## Slide 5
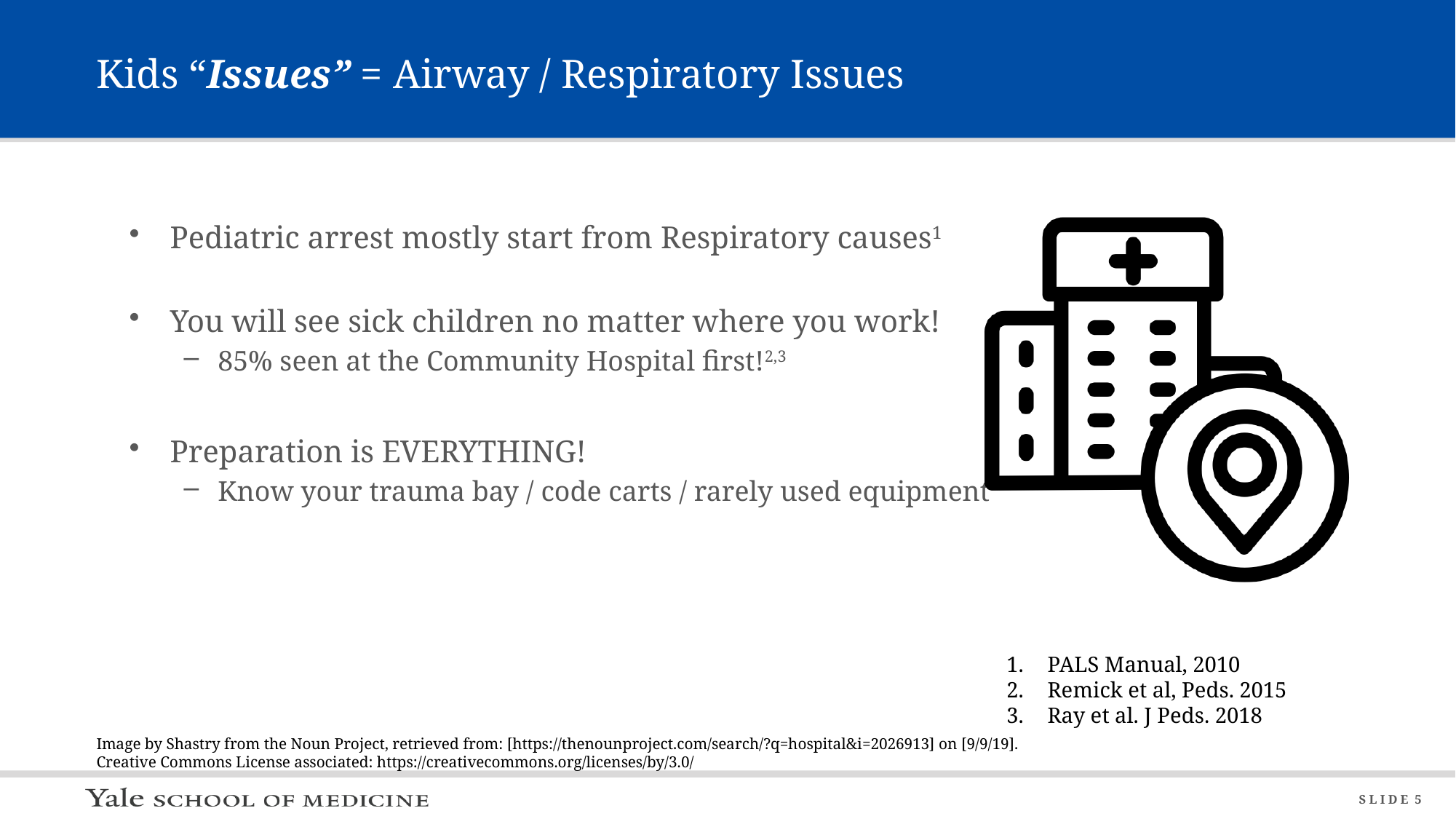

# Kids “Issues” = Airway / Respiratory Issues
Pediatric arrest mostly start from Respiratory causes1
You will see sick children no matter where you work!
85% seen at the Community Hospital first!2,3
Preparation is EVERYTHING!
Know your trauma bay / code carts / rarely used equipment
PALS Manual, 2010
Remick et al, Peds. 2015
Ray et al. J Peds. 2018
Image by Shastry from the Noun Project, retrieved from: [https://thenounproject.com/search/?q=hospital&i=2026913] on [9/9/19].
Creative Commons License associated: https://creativecommons.org/licenses/by/3.0/

## Slide 6
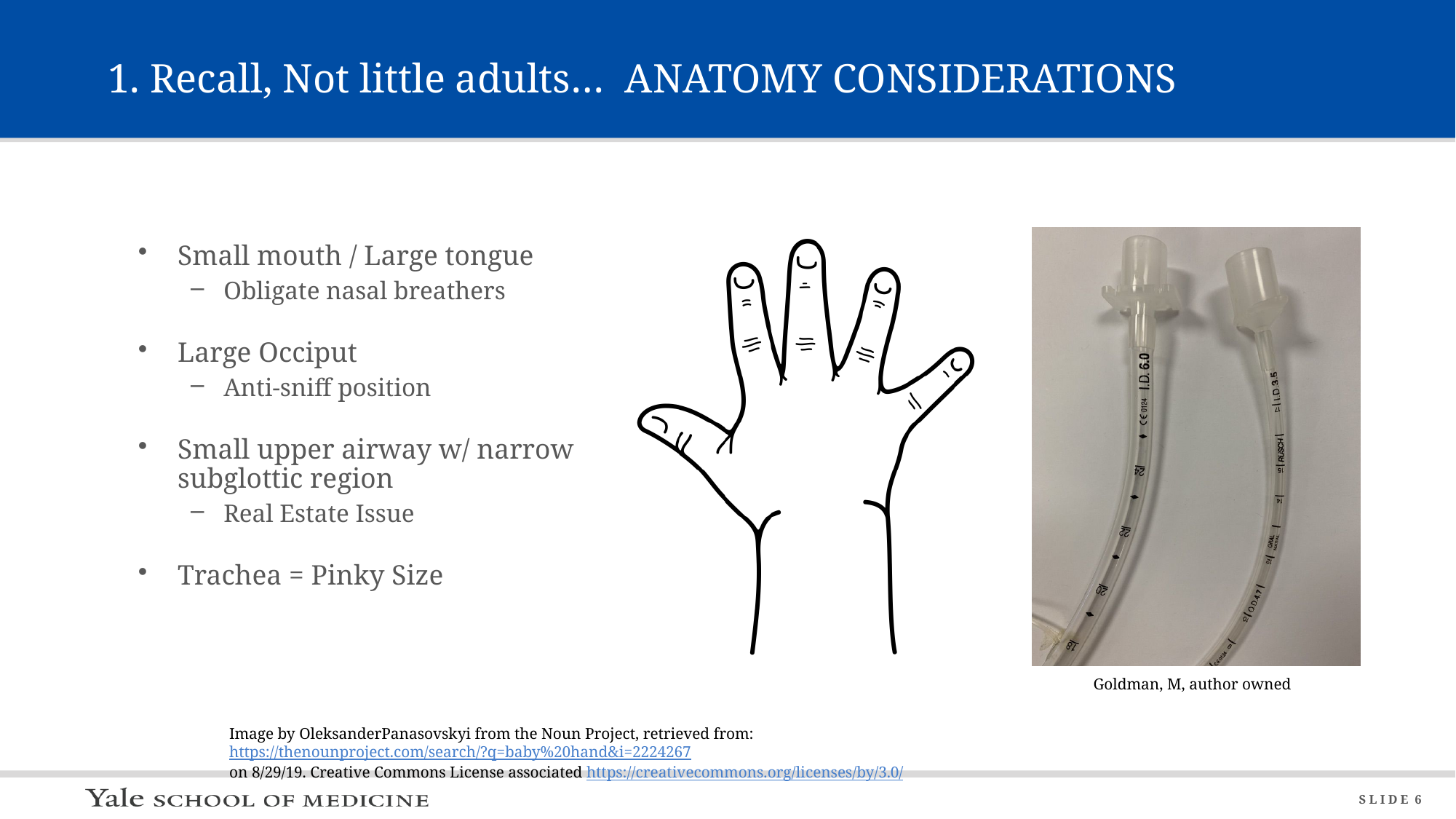

# 1. Recall, Not little adults… ANATOMY CONSIDERATIONS
Small mouth / Large tongue
Obligate nasal breathers
Large Occiput
Anti-sniff position
Small upper airway w/ narrow subglottic region
Real Estate Issue
Trachea = Pinky Size
Goldman, M, author owned
Image by OleksanderPanasovskyi from the Noun Project, retrieved from: https://thenounproject.com/search/?q=baby%20hand&i=2224267
on 8/29/19. Creative Commons License associated https://creativecommons.org/licenses/by/3.0/

## Slide 7
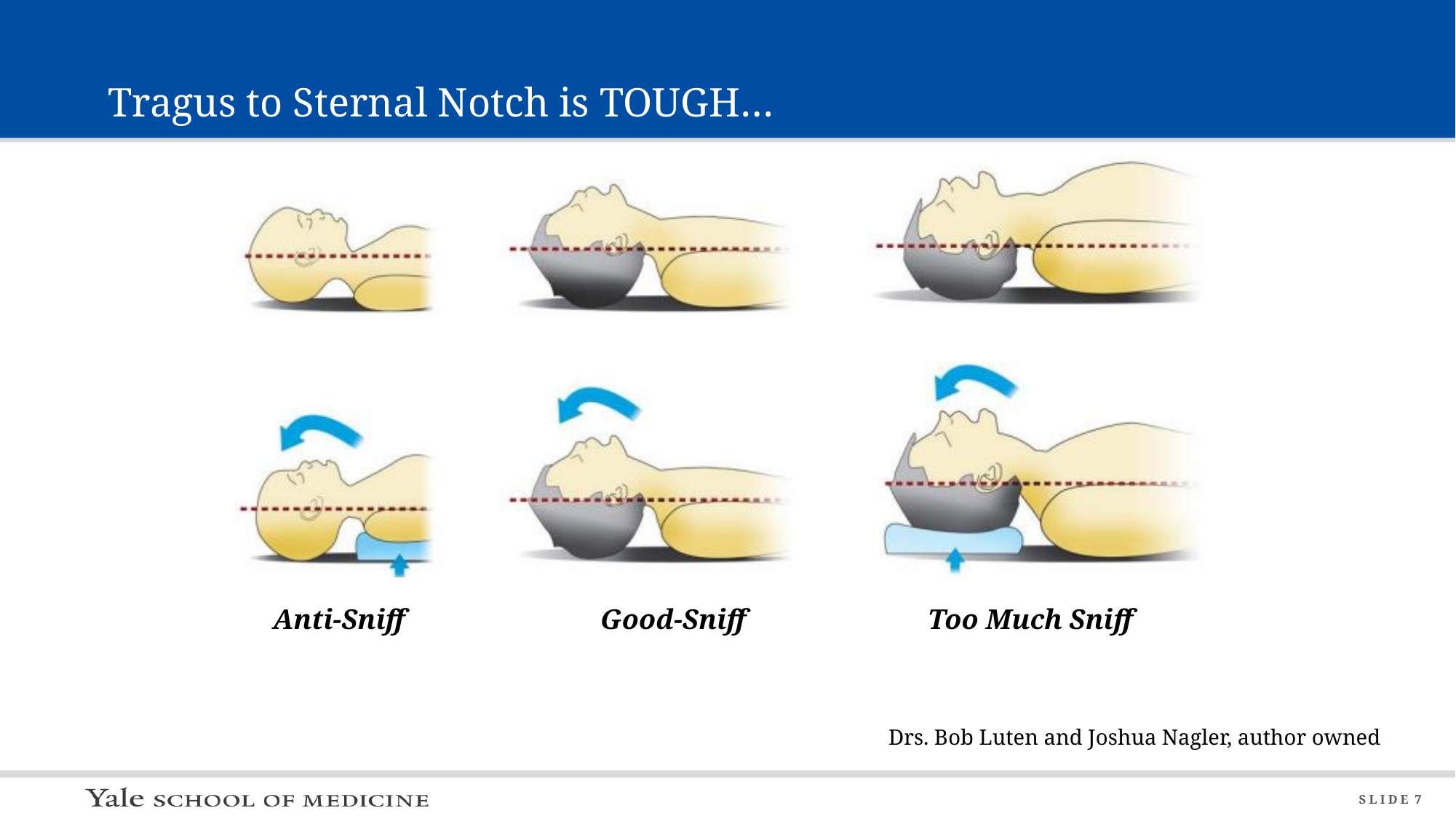

# Tragus to Sternal Notch is TOUGH…
Anti-Sniff		Good-Sniff		Too Much Sniff
Drs. Bob Luten and Joshua Nagler, author owned

## Slide 8
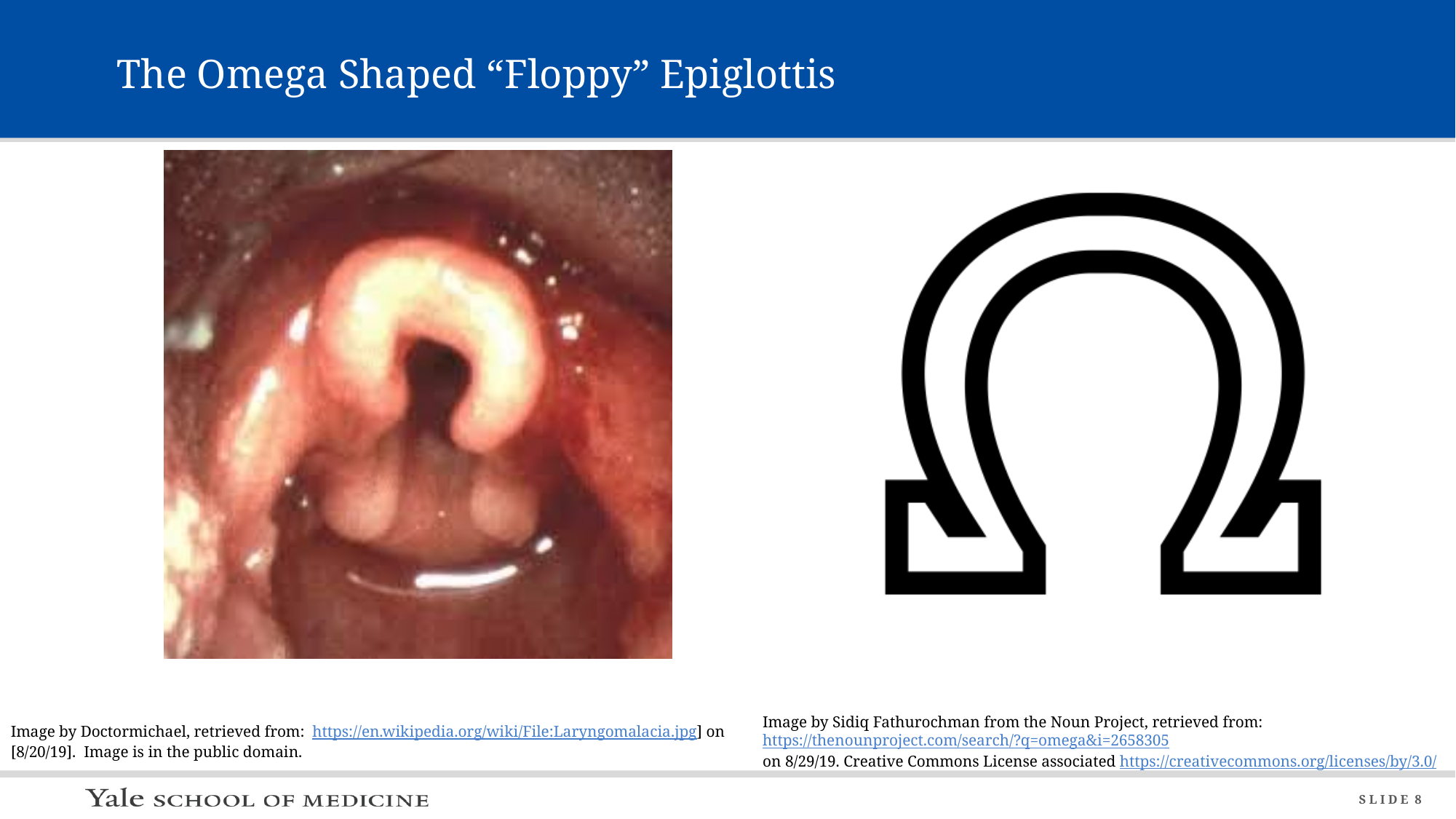

# The Omega Shaped “Floppy” Epiglottis
Image by Sidiq Fathurochman from the Noun Project, retrieved from:
https://thenounproject.com/search/?q=omega&i=2658305
on 8/29/19. Creative Commons License associated https://creativecommons.org/licenses/by/3.0/
Image by Doctormichael, retrieved from: https://en.wikipedia.org/wiki/File:Laryngomalacia.jpg] on [8/20/19]. Image is in the public domain.

## Slide 9
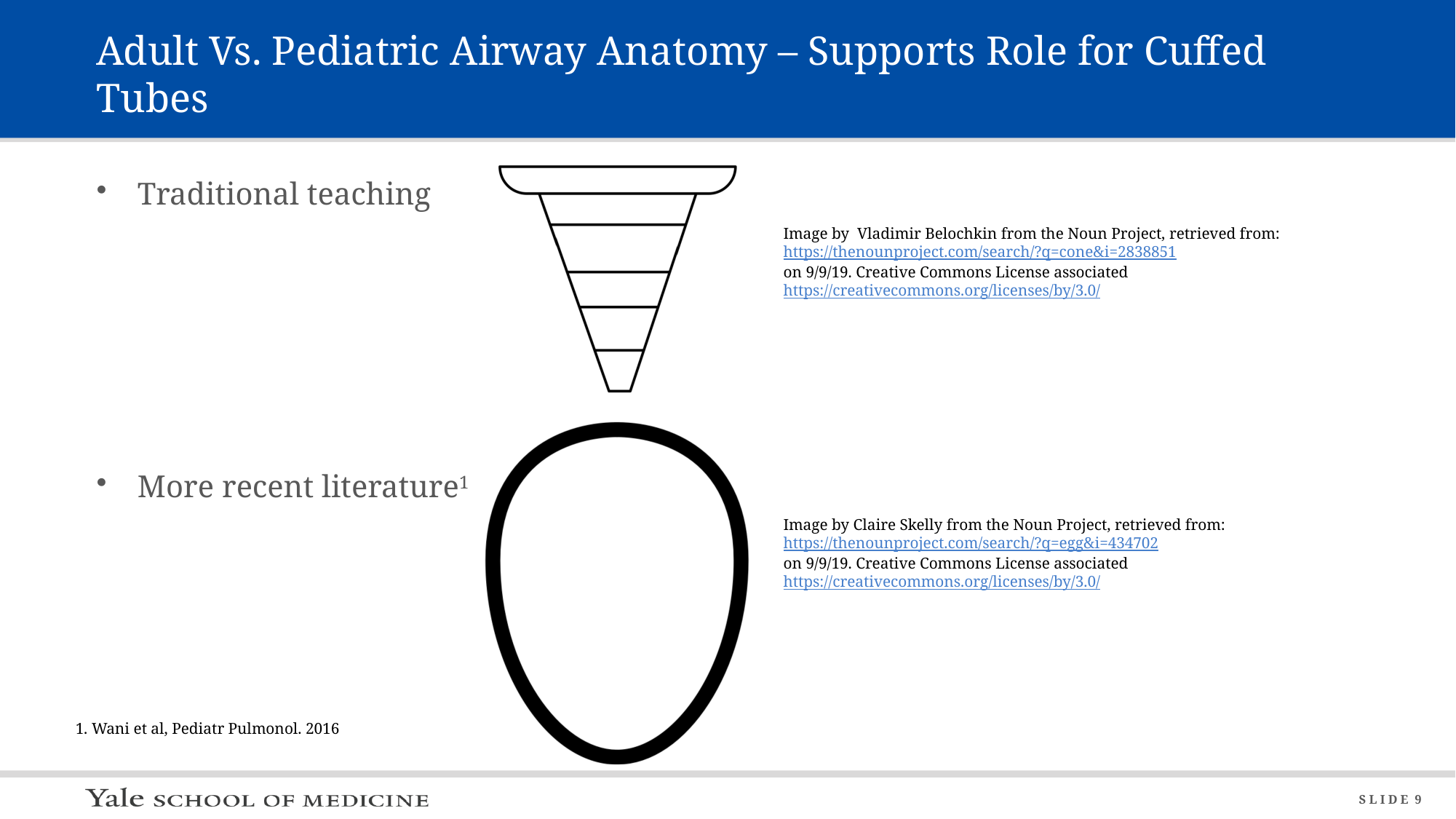

# Adult Vs. Pediatric Airway Anatomy – Supports Role for Cuffed Tubes
Traditional teaching
More recent literature1
Image by Vladimir Belochkin from the Noun Project, retrieved from:
https://thenounproject.com/search/?q=cone&i=2838851
on 9/9/19. Creative Commons License associated https://creativecommons.org/licenses/by/3.0/
Image by Claire Skelly from the Noun Project, retrieved from:
https://thenounproject.com/search/?q=egg&i=434702
on 9/9/19. Creative Commons License associated https://creativecommons.org/licenses/by/3.0/
1. Wani et al, Pediatr Pulmonol. 2016

## Slide 10
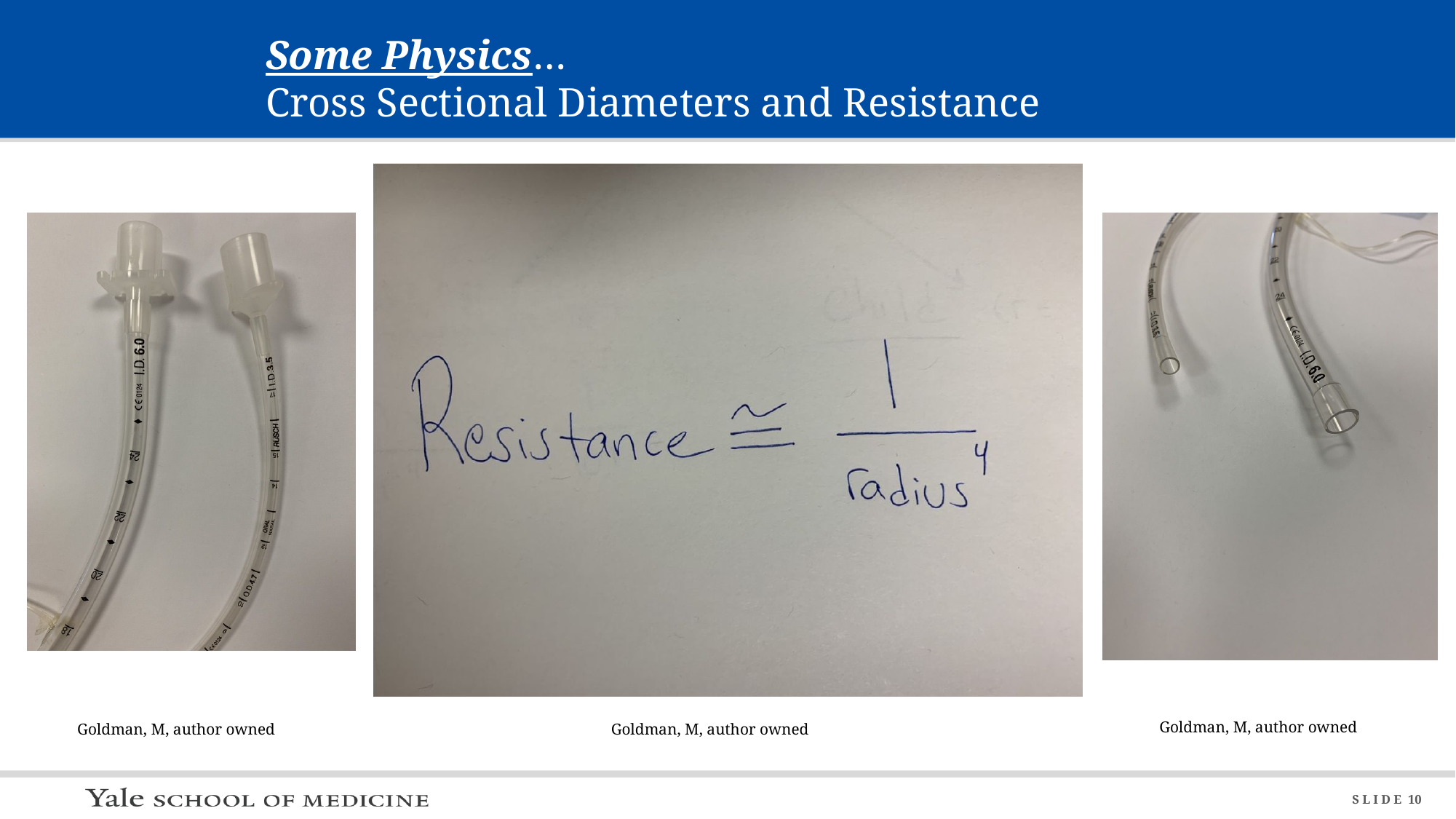

# Some Physics… Cross Sectional Diameters and Resistance
Goldman, M, author owned
Goldman, M, author owned
Goldman, M, author owned

## Slide 11
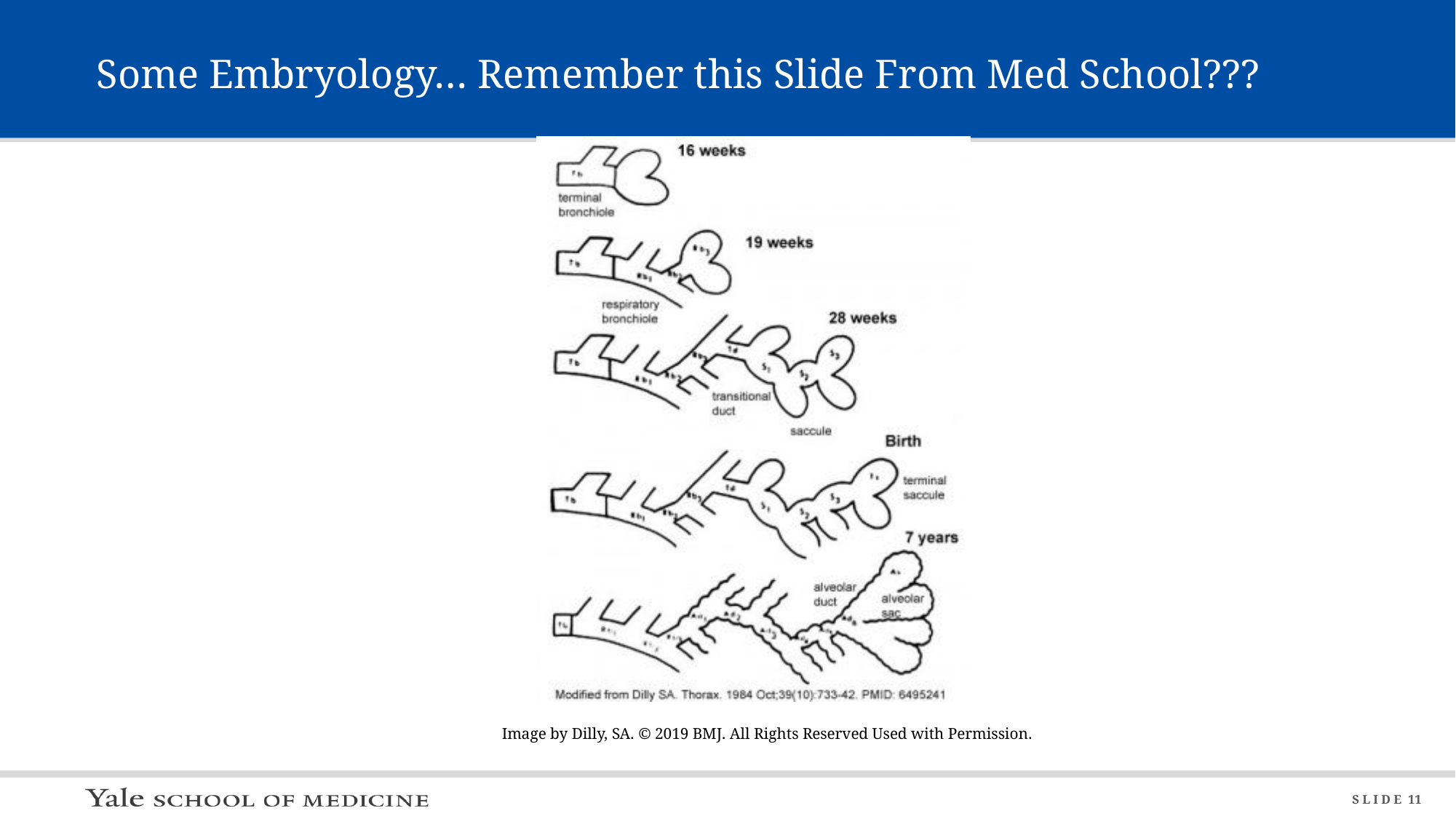

# Some Embryology… Remember this Slide From Med School???
Image by Dilly, SA. © 2019 BMJ. All Rights Reserved Used with Permission.

## Slide 12
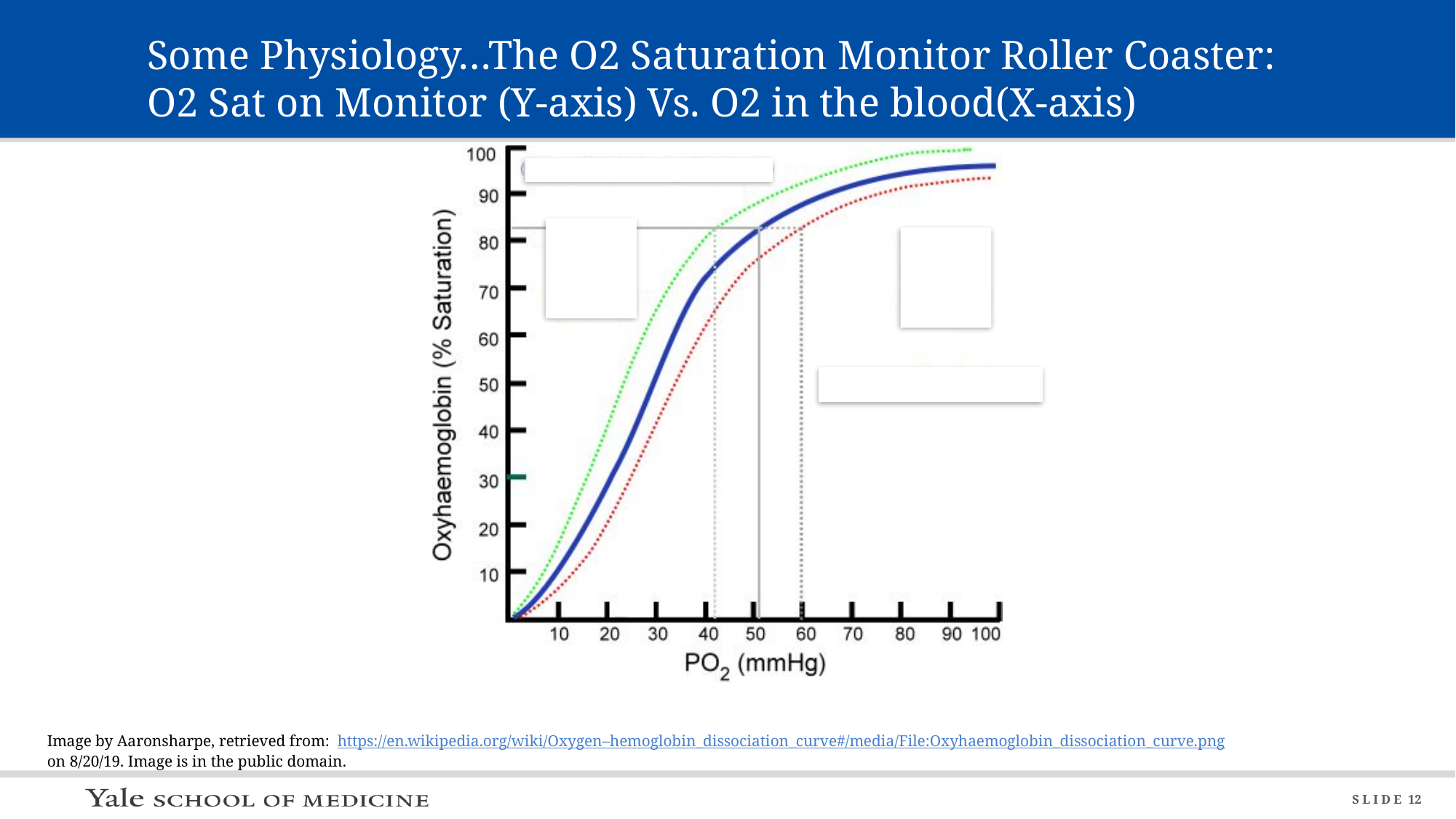

# Some Physiology…The O2 Saturation Monitor Roller Coaster: O2 Sat on Monitor (Y-axis) Vs. O2 in the blood(X-axis)
Image by Aaronsharpe, retrieved from: https://en.wikipedia.org/wiki/Oxygen–hemoglobin_dissociation_curve#/media/File:Oxyhaemoglobin_dissociation_curve.png
on 8/20/19. Image is in the public domain.

## Slide 13
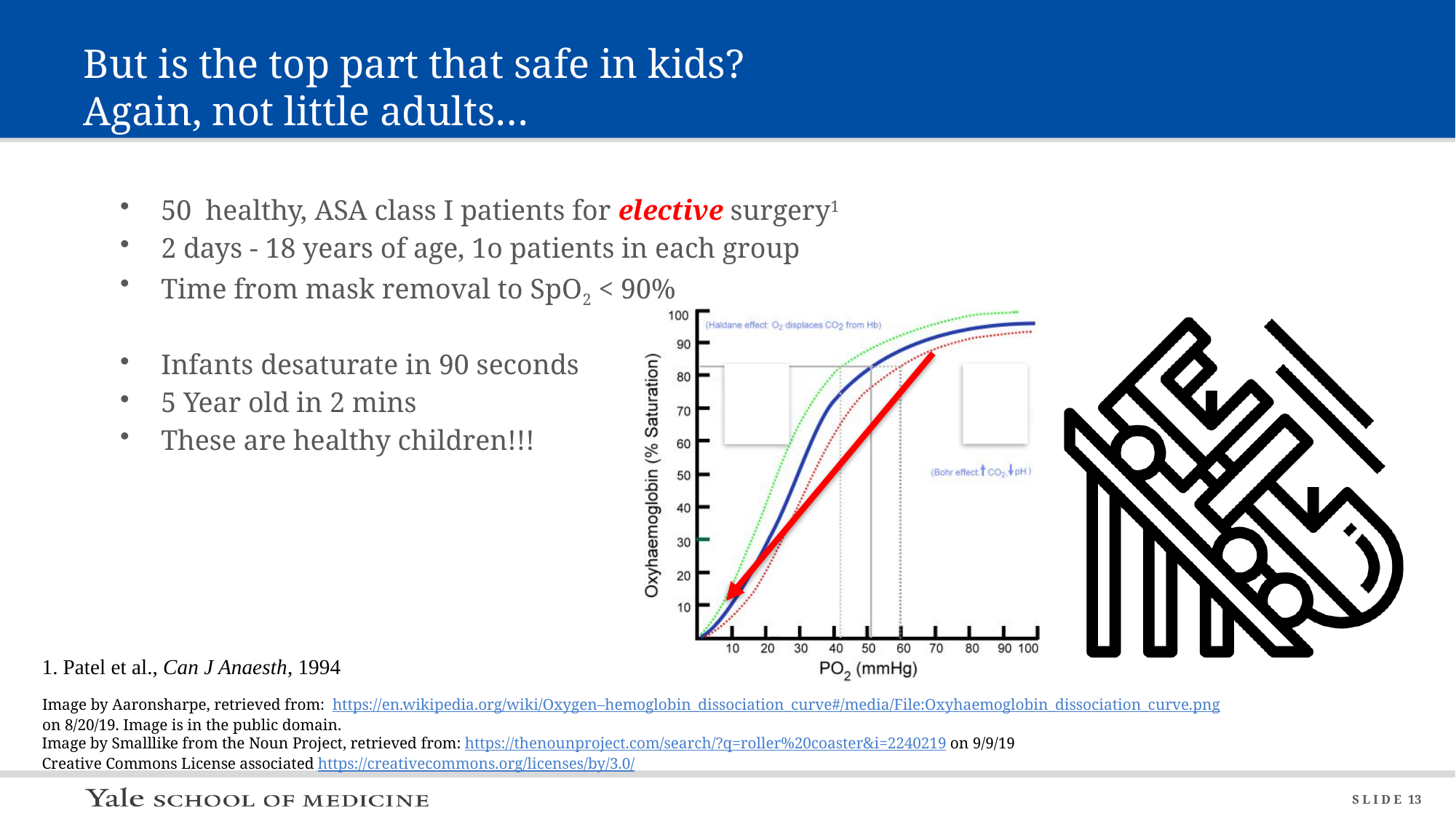

# But is the top part that safe in kids? Again, not little adults…
50 healthy, ASA class I patients for elective surgery1
2 days - 18 years of age, 1o patients in each group
Time from mask removal to SpO2 < 90%
Infants desaturate in 90 seconds
5 Year old in 2 mins
These are healthy children!!!
1. Patel et al., Can J Anaesth, 1994
Image by Aaronsharpe, retrieved from: https://en.wikipedia.org/wiki/Oxygen–hemoglobin_dissociation_curve#/media/File:Oxyhaemoglobin_dissociation_curve.png
on 8/20/19. Image is in the public domain.
Image by Smalllike from the Noun Project, retrieved from: https://thenounproject.com/search/?q=roller%20coaster&i=2240219 on 9/9/19
Creative Commons License associated https://creativecommons.org/licenses/by/3.0/

## Slide 14
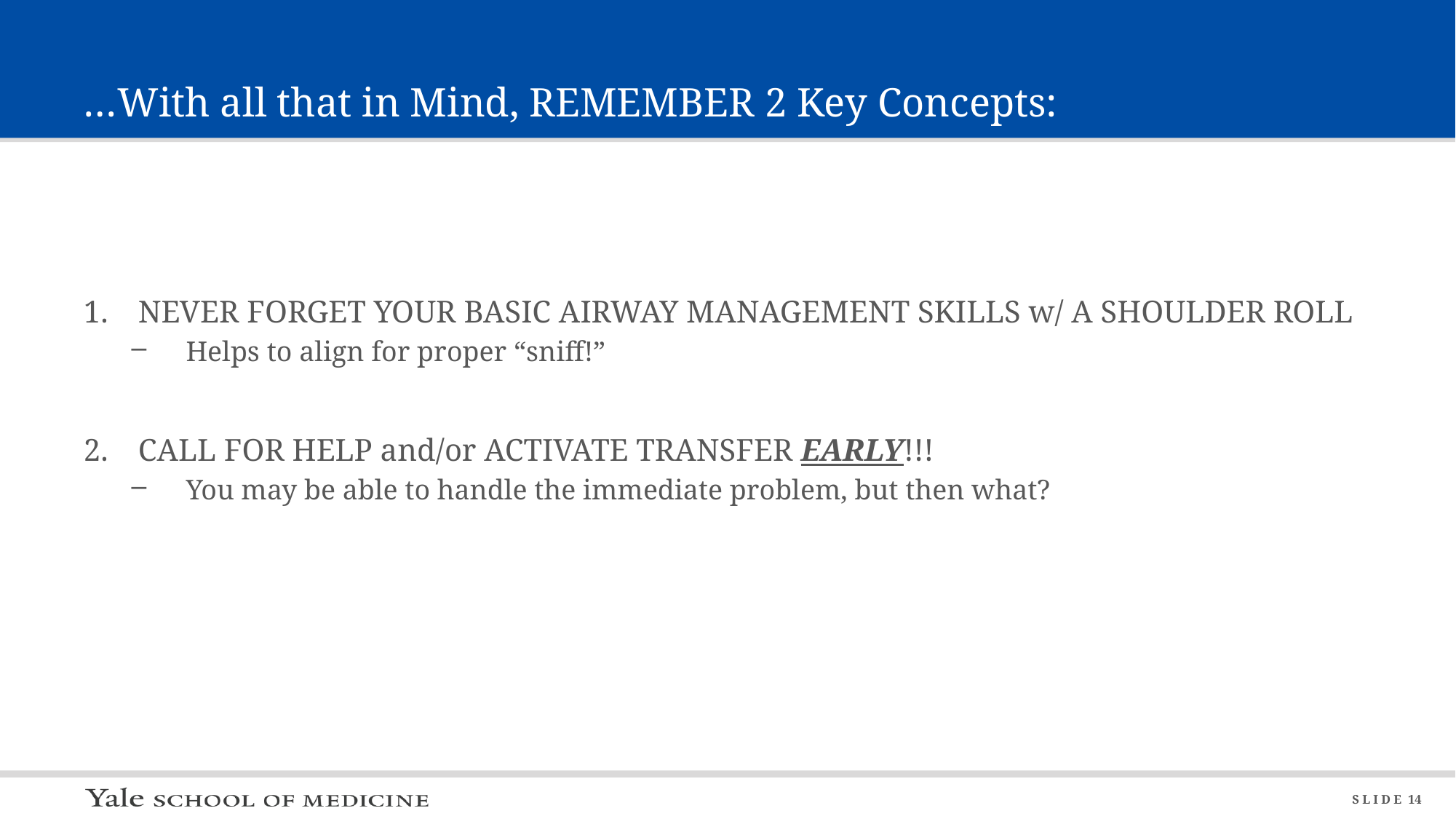

# …With all that in Mind, REMEMBER 2 Key Concepts:
NEVER FORGET YOUR BASIC AIRWAY MANAGEMENT SKILLS w/ A SHOULDER ROLL
Helps to align for proper “sniff!”
CALL FOR HELP and/or ACTIVATE TRANSFER EARLY!!!
You may be able to handle the immediate problem, but then what?

## Slide 15
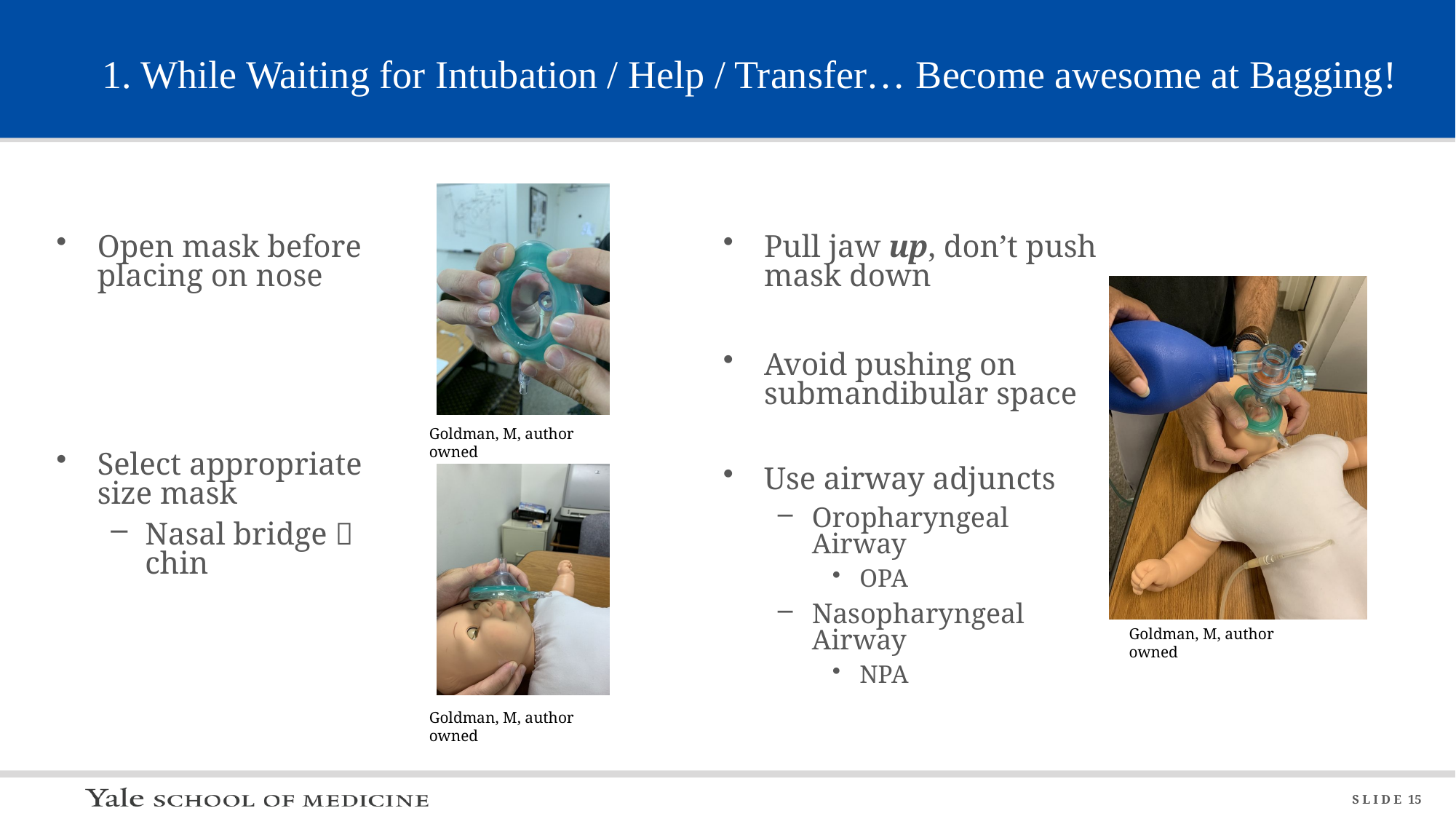

# 1. While Waiting for Intubation / Help / Transfer… Become awesome at Bagging!
Open mask before placing on nose
Select appropriate size mask
Nasal bridge  chin
Pull jaw up, don’t push mask down
Avoid pushing on submandibular space
Use airway adjuncts
Oropharyngeal Airway
OPA
Nasopharyngeal Airway
NPA
Goldman, M, author owned
Goldman, M, author owned
Goldman, M, author owned

## Slide 16
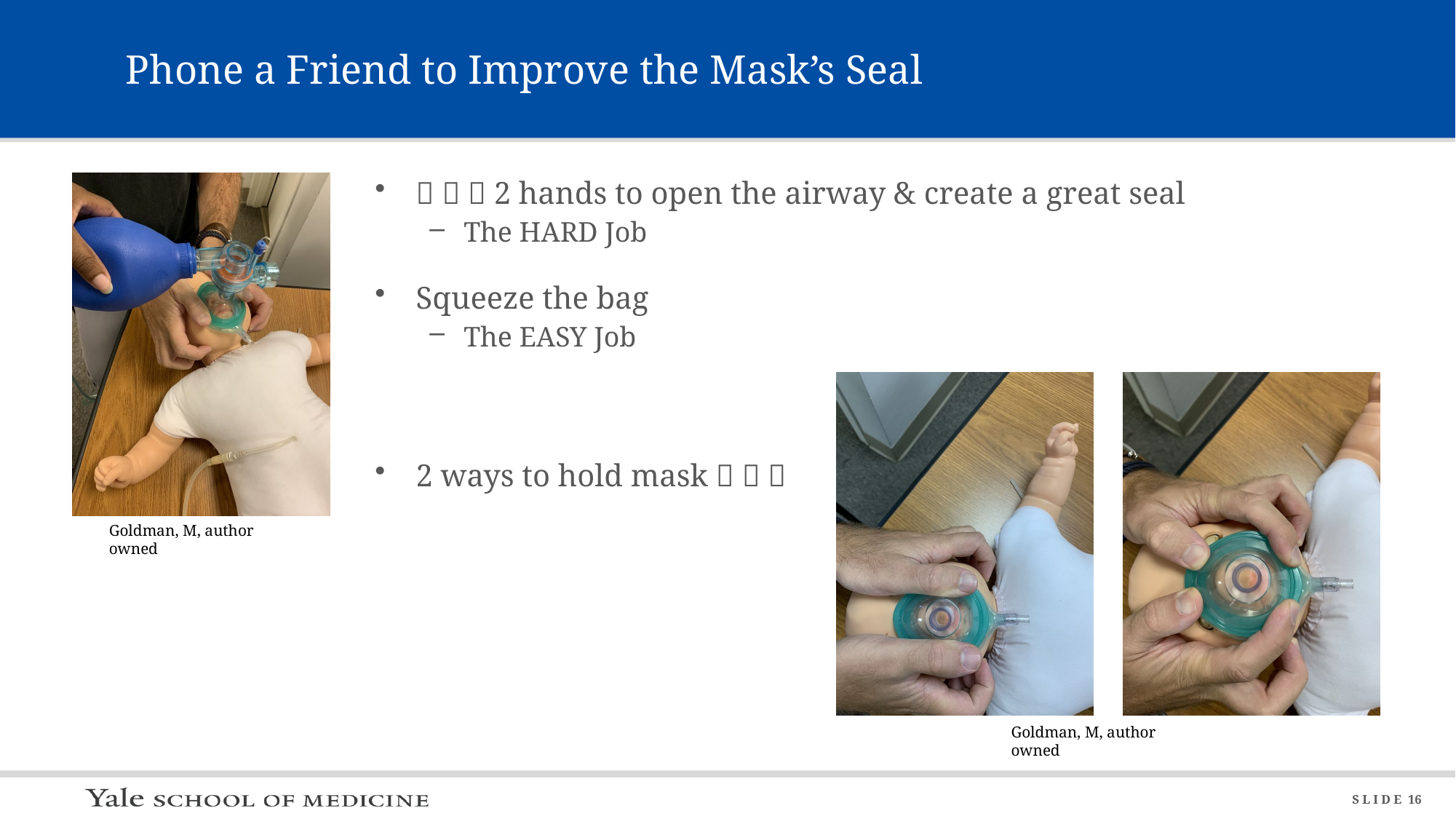

# Phone a Friend to Improve the Mask’s Seal
   2 hands to open the airway & create a great seal
The HARD Job
Squeeze the bag
The EASY Job
2 ways to hold mask   
Goldman, M, author owned
Goldman, M, author owned

## Slide 17
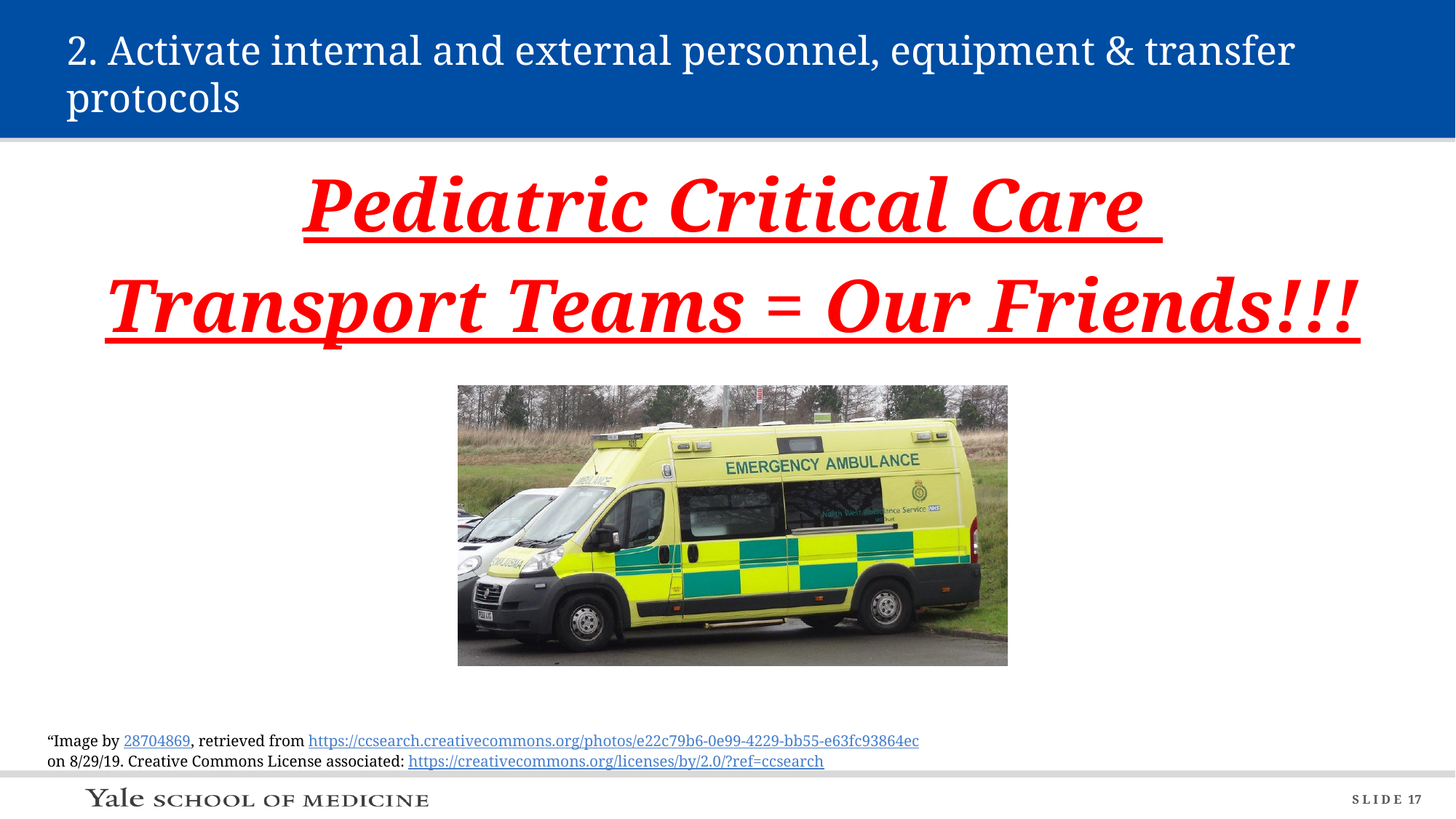

# 2. Activate internal and external personnel, equipment & transfer protocols
Pediatric Critical Care
Transport Teams = Our Friends!!!
“Image by 28704869, retrieved from https://ccsearch.creativecommons.org/photos/e22c79b6-0e99-4229-bb55-e63fc93864ec
on 8/29/19. Creative Commons License associated: https://creativecommons.org/licenses/by/2.0/?ref=ccsearch

## Slide 18
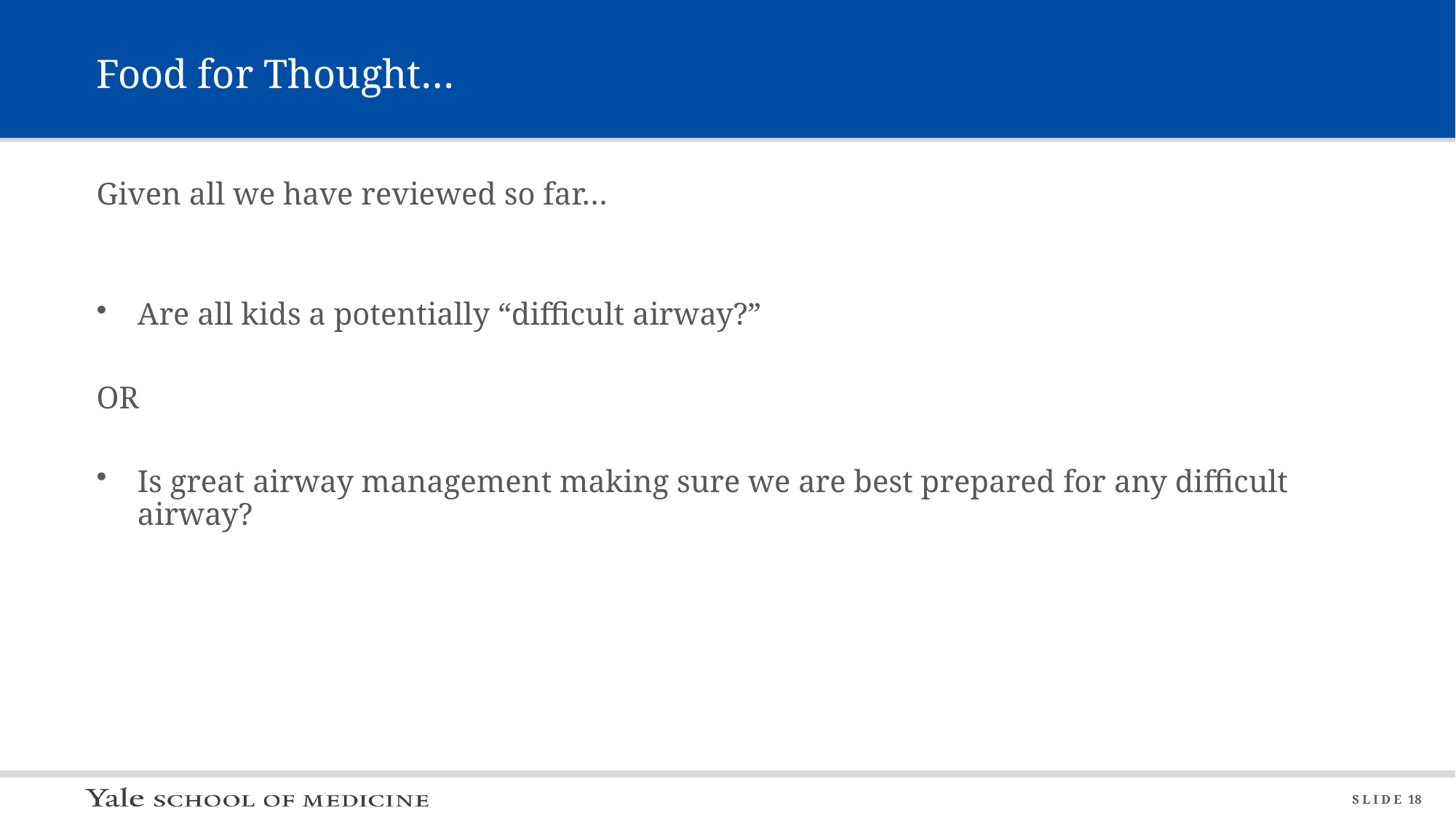

# Food for Thought…
Given all we have reviewed so far…
Are all kids a potentially “difficult airway?”
OR
Is great airway management making sure we are best prepared for any difficult airway?

## Slide 19
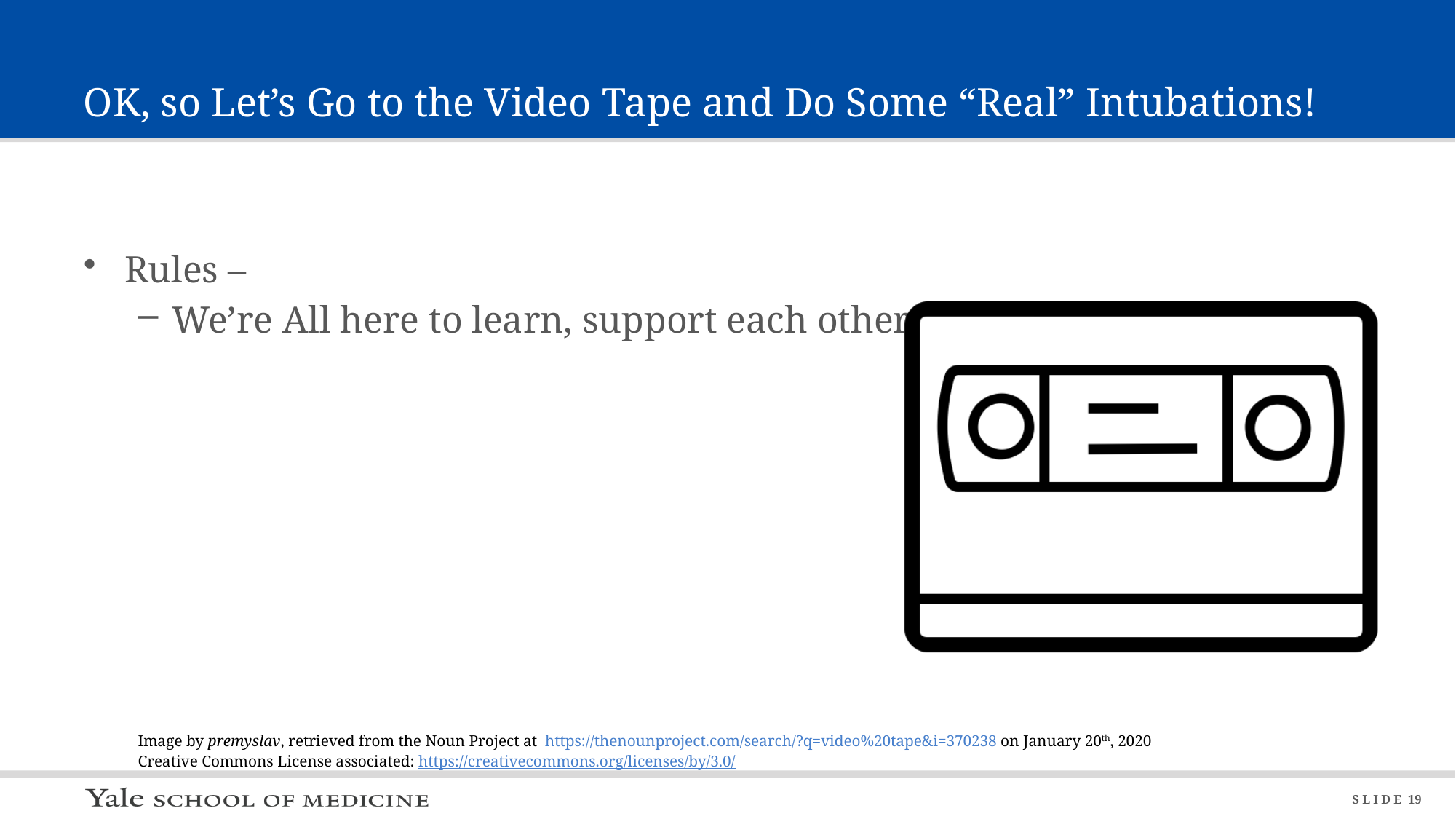

# OK, so Let’s Go to the Video Tape and Do Some “Real” Intubations!
Rules –
We’re All here to learn, support each other
Image by premyslav, retrieved from the Noun Project at https://thenounproject.com/search/?q=video%20tape&i=370238 on January 20th, 2020
Creative Commons License associated: https://creativecommons.org/licenses/by/3.0/

## Slide 20
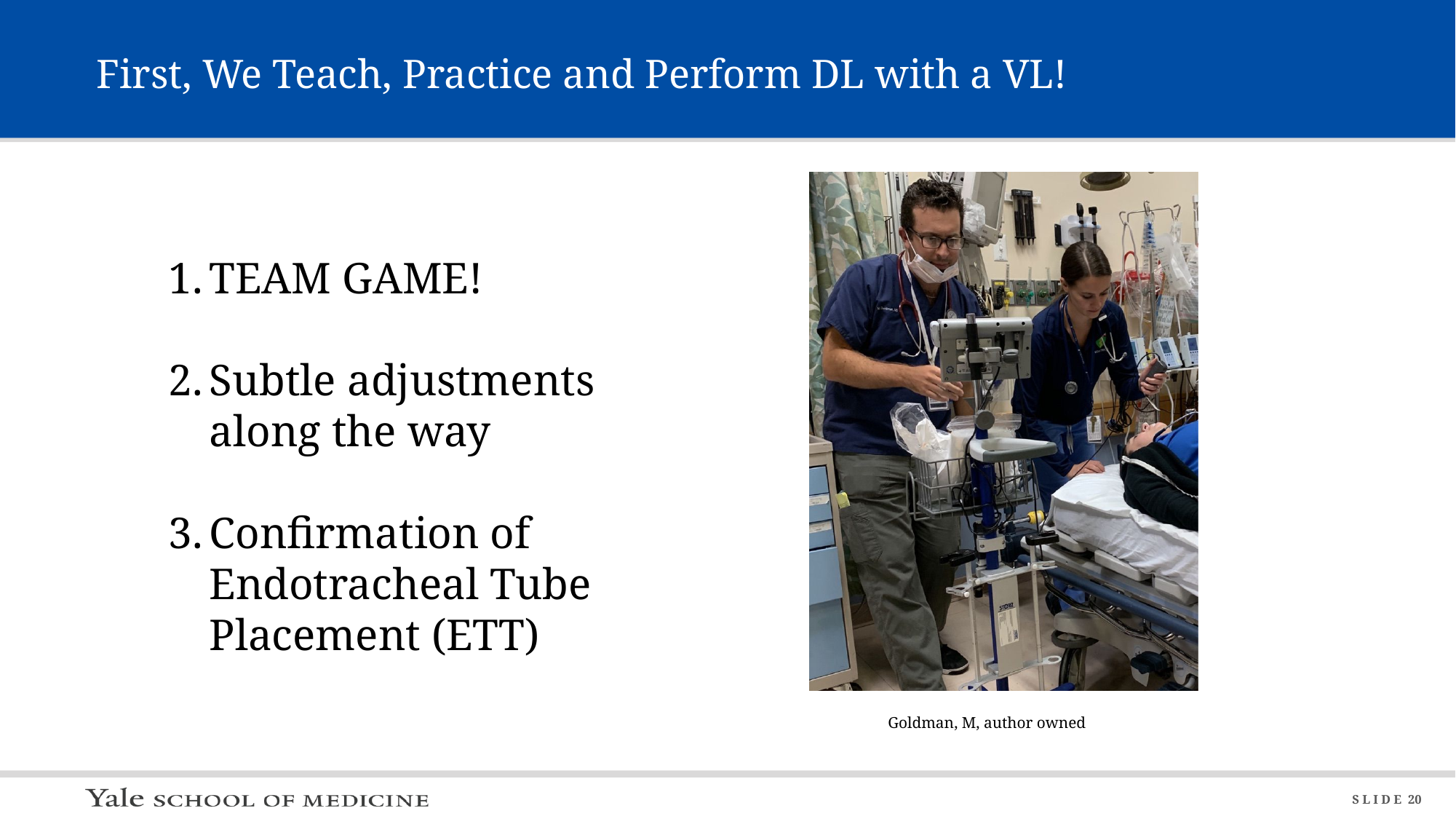

# First, We Teach, Practice and Perform DL with a VL!
TEAM GAME!
Subtle adjustments along the way
Confirmation of Endotracheal Tube Placement (ETT)
Goldman, M, author owned

## Slide 21
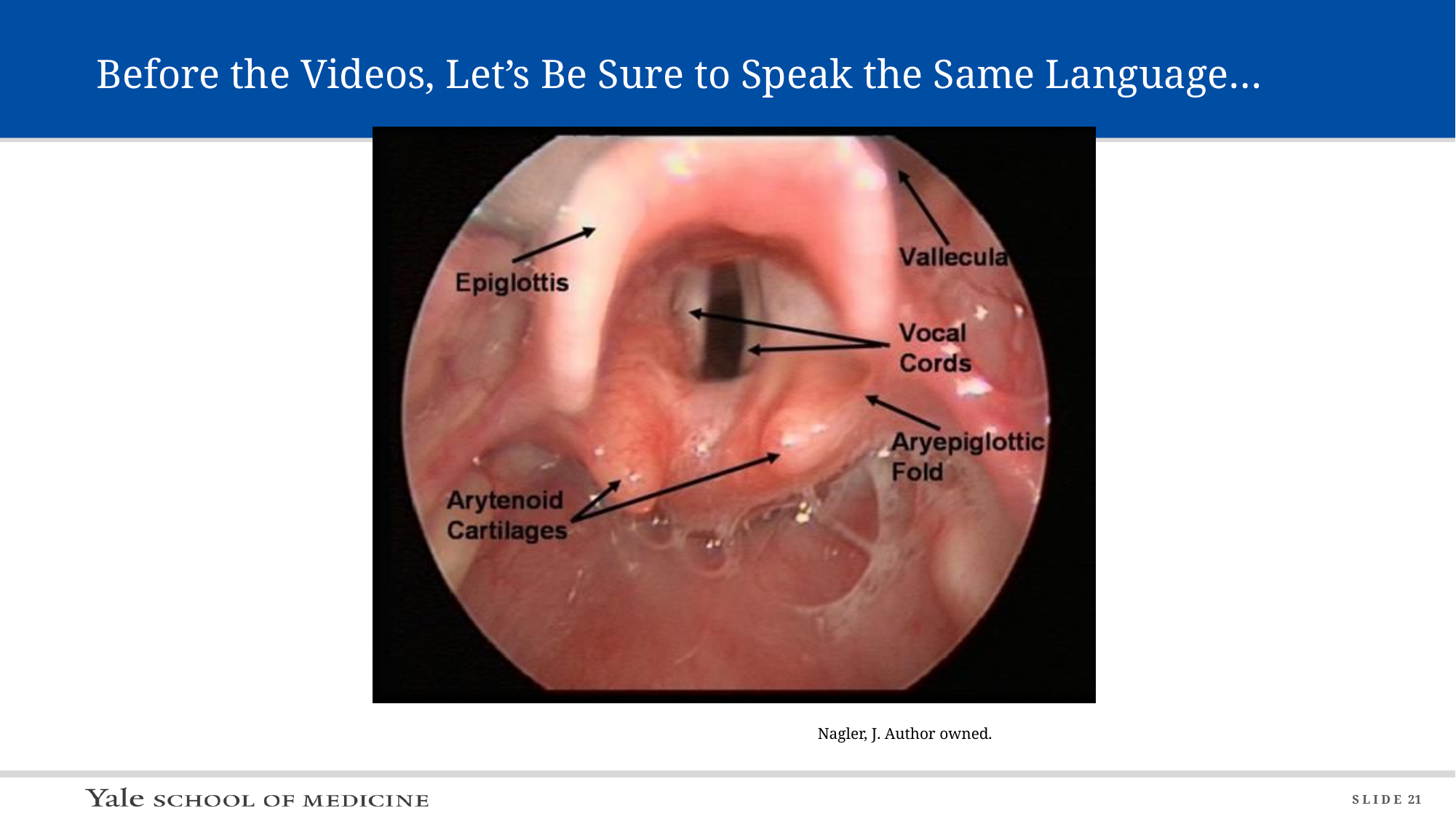

# Before the Videos, Let’s Be Sure to Speak the Same Language…
Nagler, J. Author owned.

## Slide 22
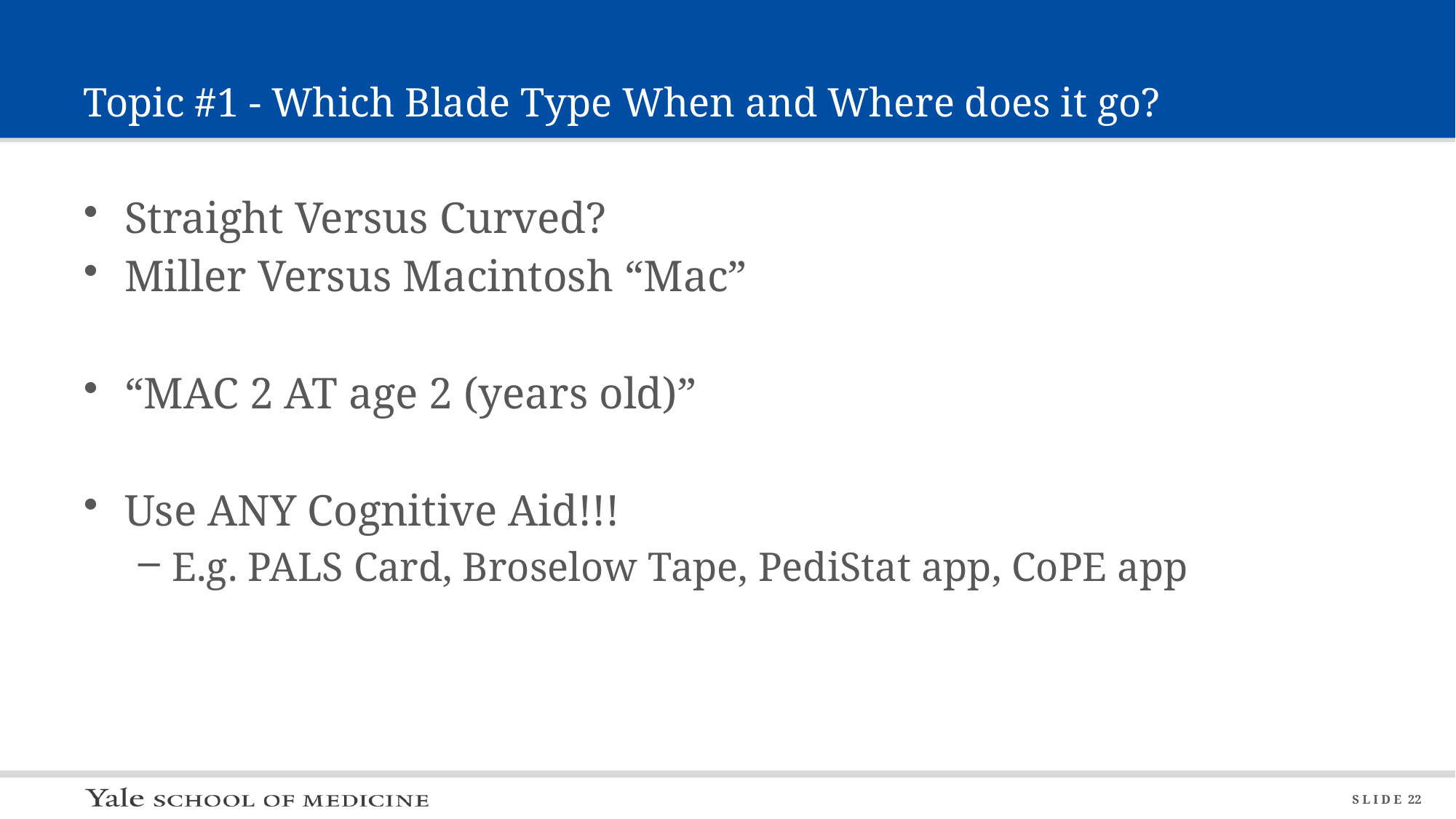

# Topic #1 - Which Blade Type When and Where does it go?
Straight Versus Curved?
Miller Versus Macintosh “Mac”
“MAC 2 AT age 2 (years old)”
Use ANY Cognitive Aid!!!
E.g. PALS Card, Broselow Tape, PediStat app, CoPE app

## Slide 23
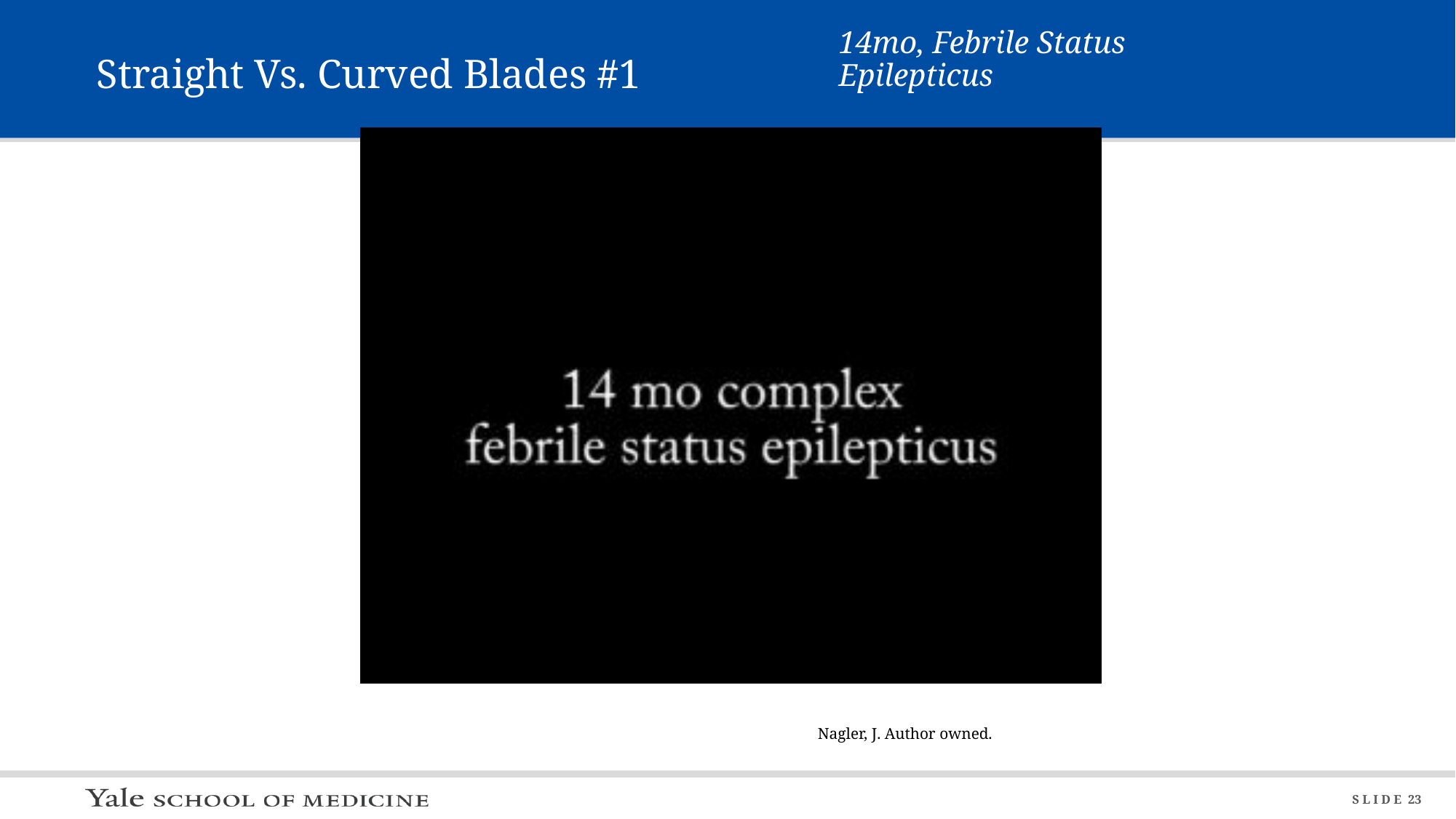

# Straight Vs. Curved Blades #1
14mo, Febrile Status Epilepticus
Nagler, J. Author owned.

## Slide 24
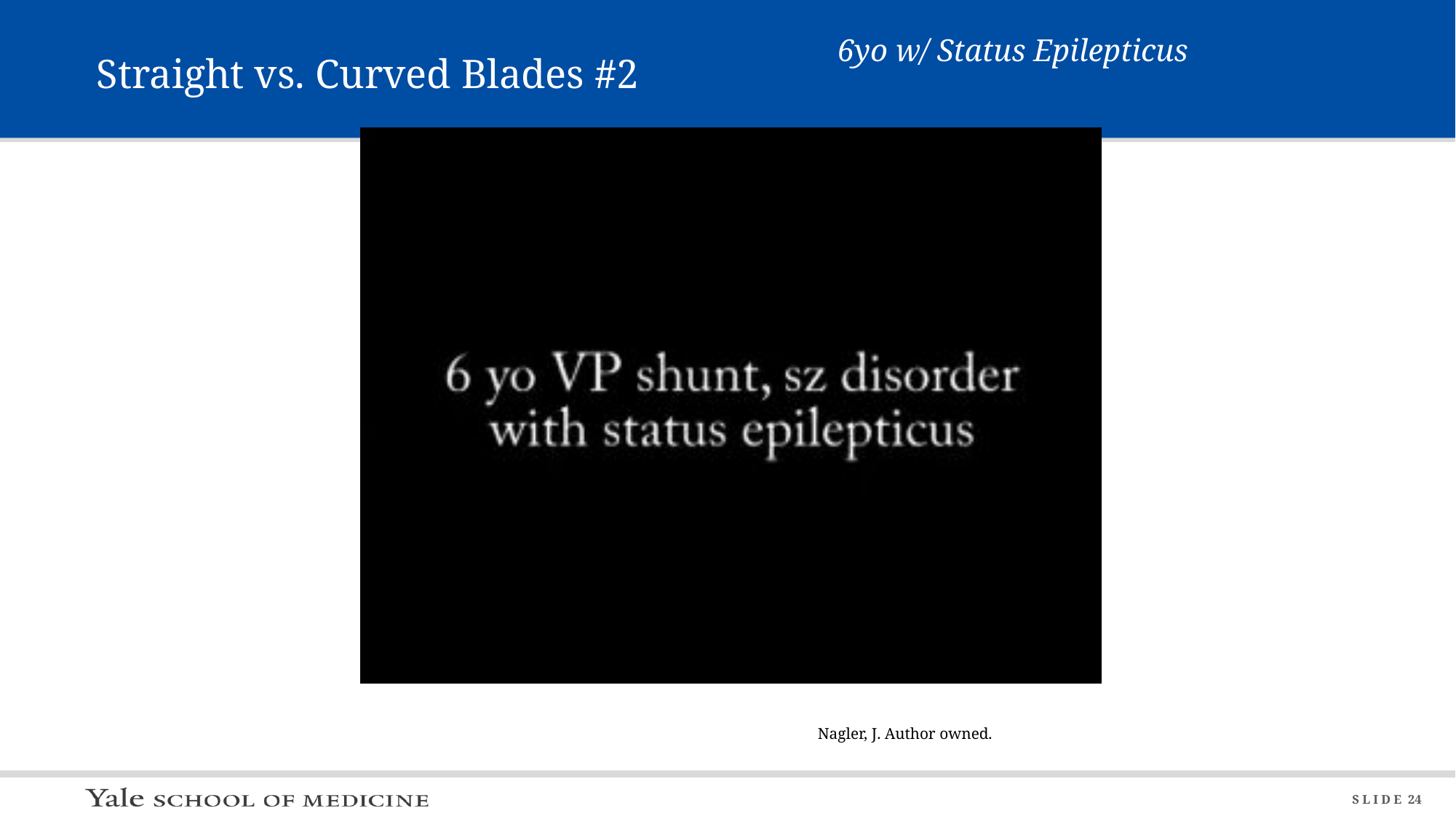

# Straight vs. Curved Blades #2
6yo w/ Status Epilepticus
Nagler, J. Author owned.

## Slide 25
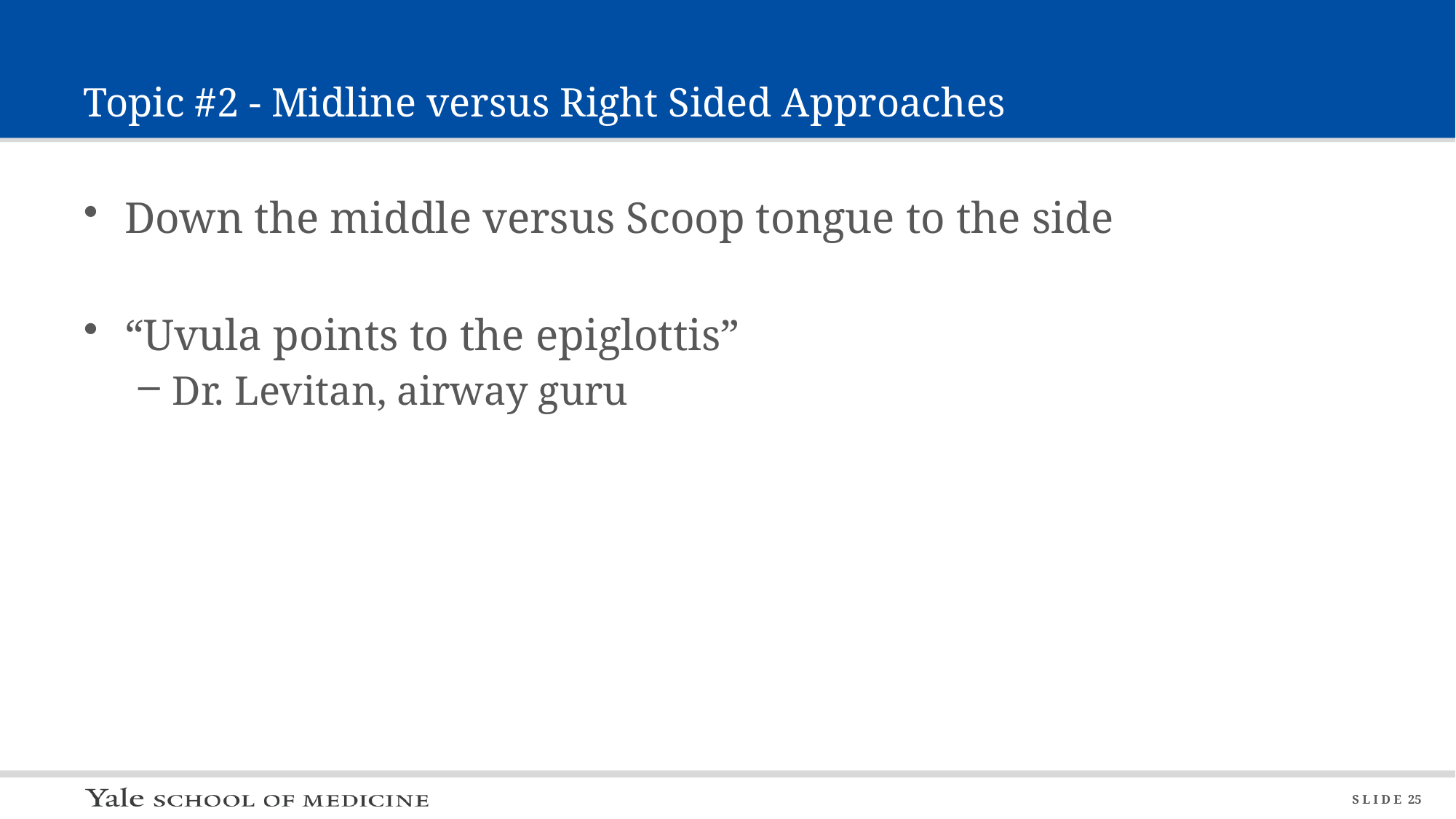

# Topic #2 - Midline versus Right Sided Approaches
Down the middle versus Scoop tongue to the side
“Uvula points to the epiglottis”
Dr. Levitan, airway guru

## Slide 26
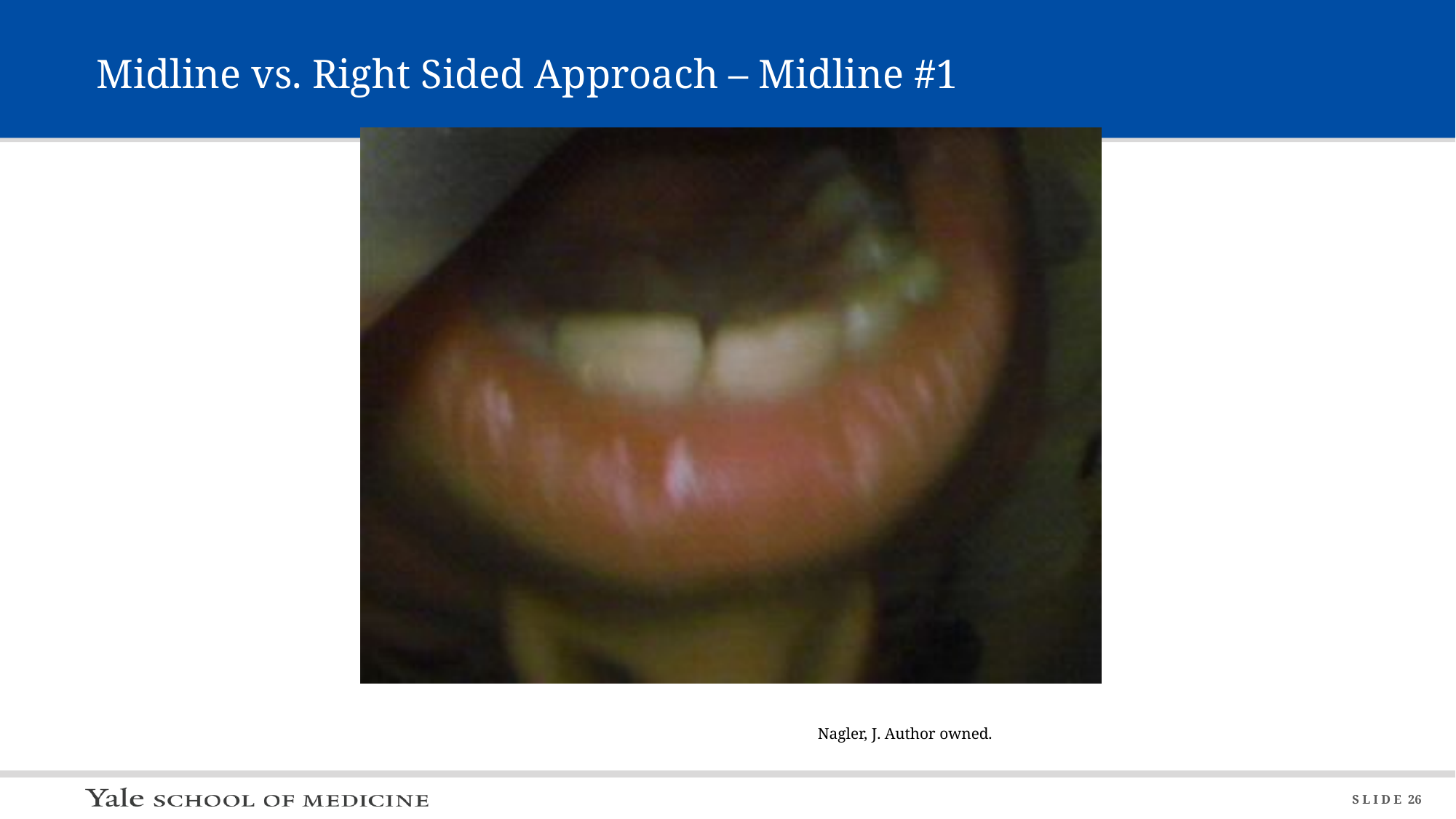

# Midline vs. Right Sided Approach – Midline #1
Nagler, J. Author owned.

## Slide 27
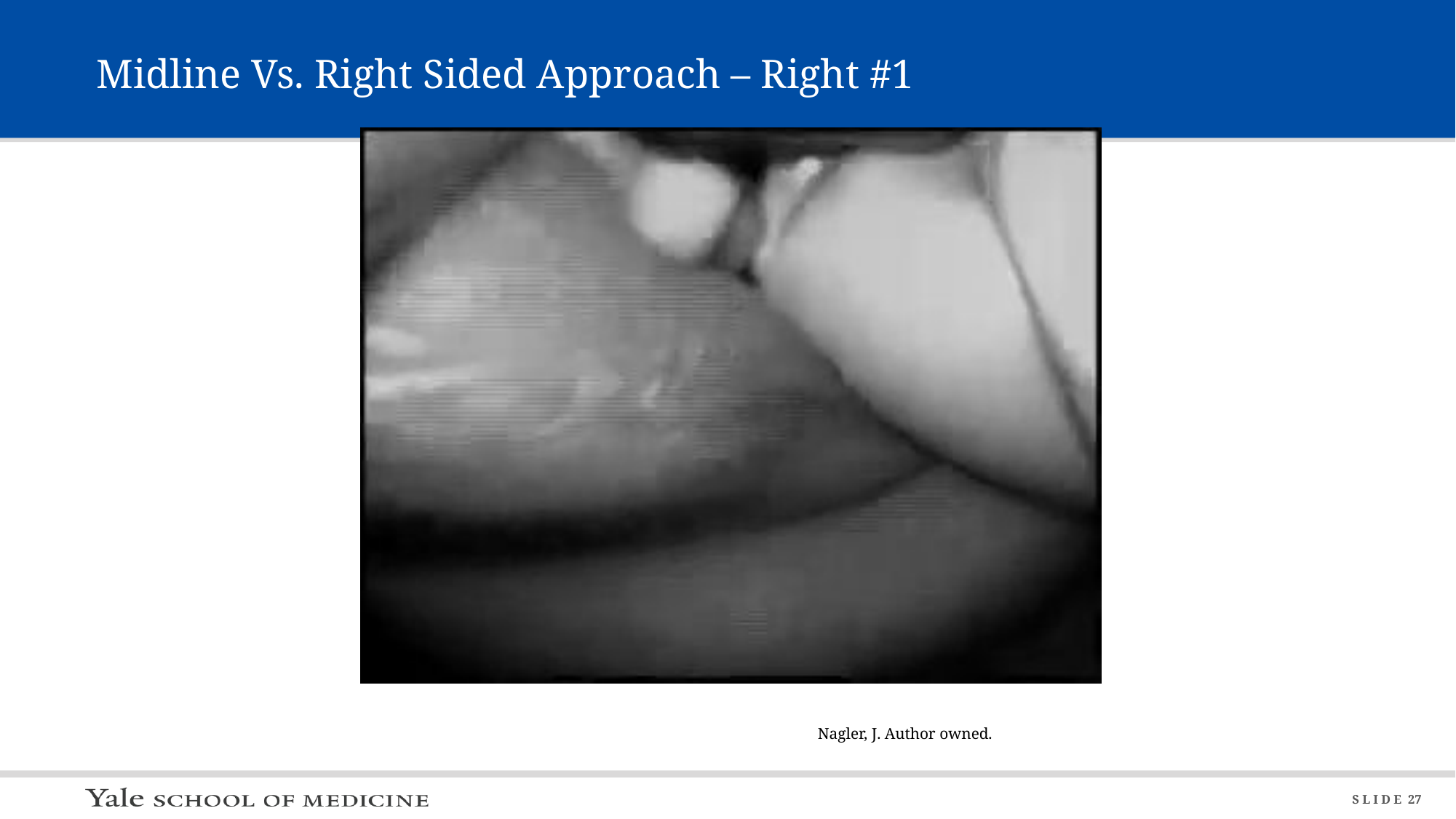

# Midline Vs. Right Sided Approach – Right #1
Nagler, J. Author owned.

## Slide 28
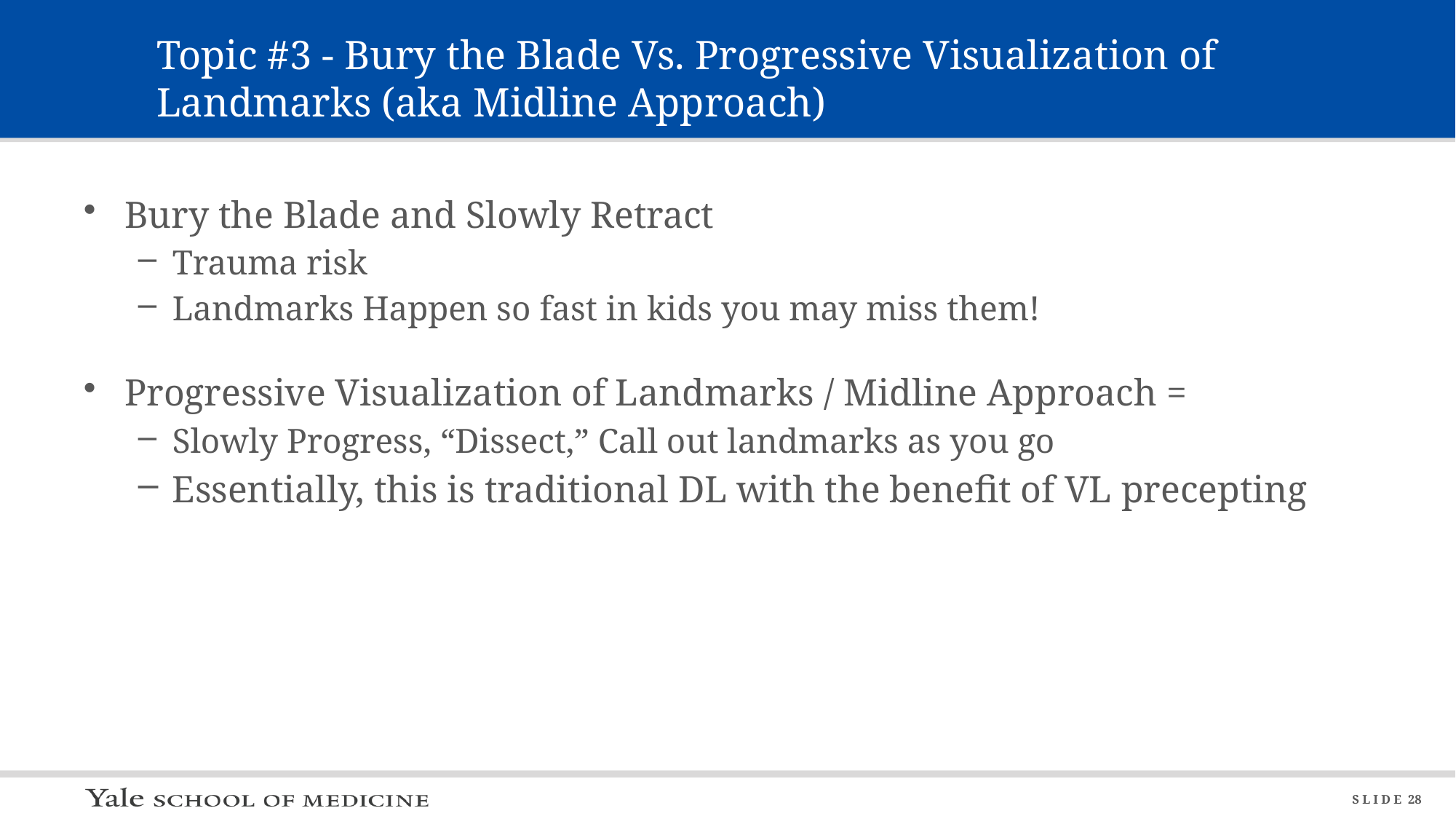

# Topic #3 - Bury the Blade Vs. Progressive Visualization of Landmarks (aka Midline Approach)
Bury the Blade and Slowly Retract
Trauma risk
Landmarks Happen so fast in kids you may miss them!
Progressive Visualization of Landmarks / Midline Approach =
Slowly Progress, “Dissect,” Call out landmarks as you go
Essentially, this is traditional DL with the benefit of VL precepting

## Slide 29
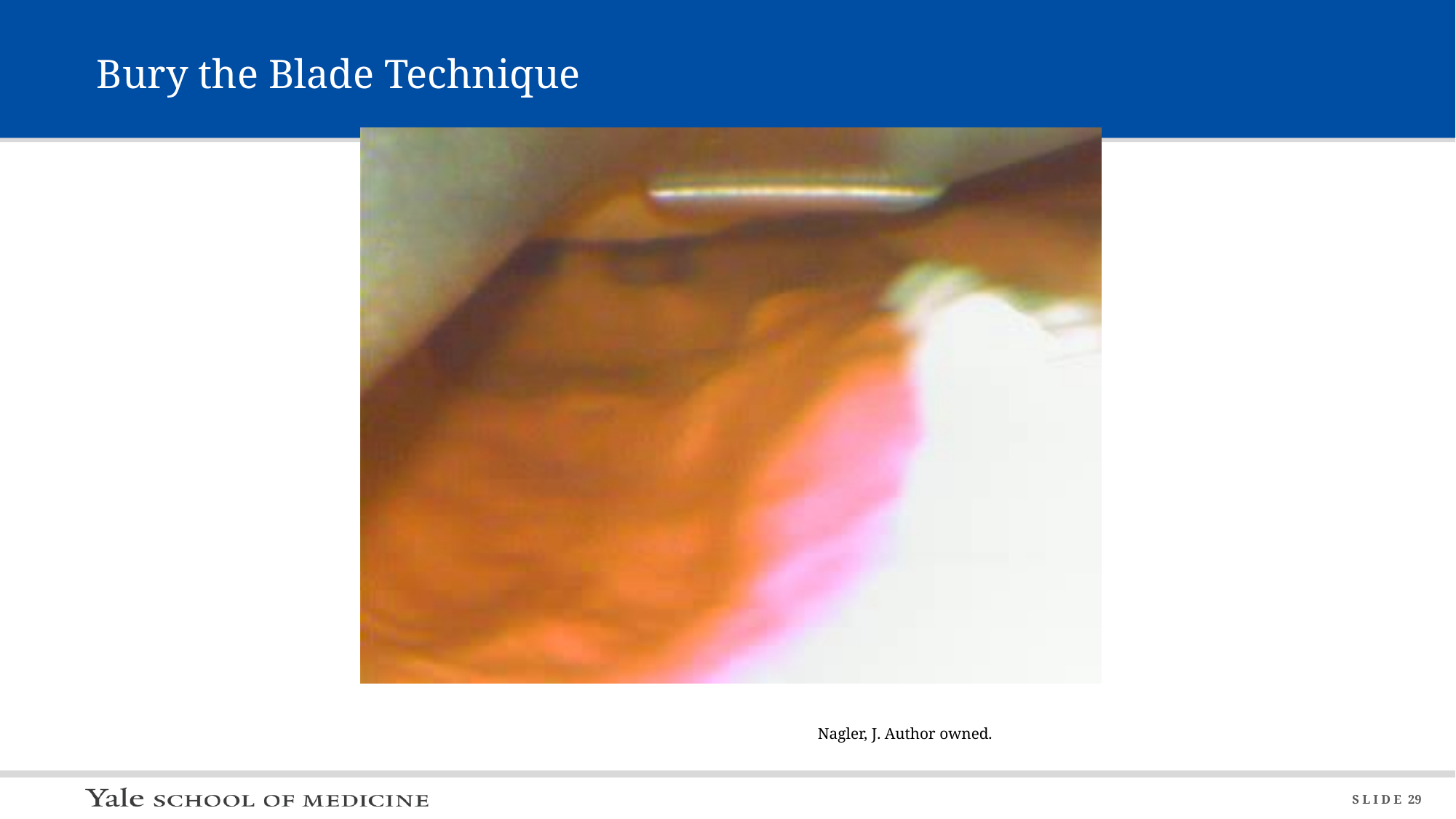

# Bury the Blade Technique
Nagler, J. Author owned.

## Slide 30
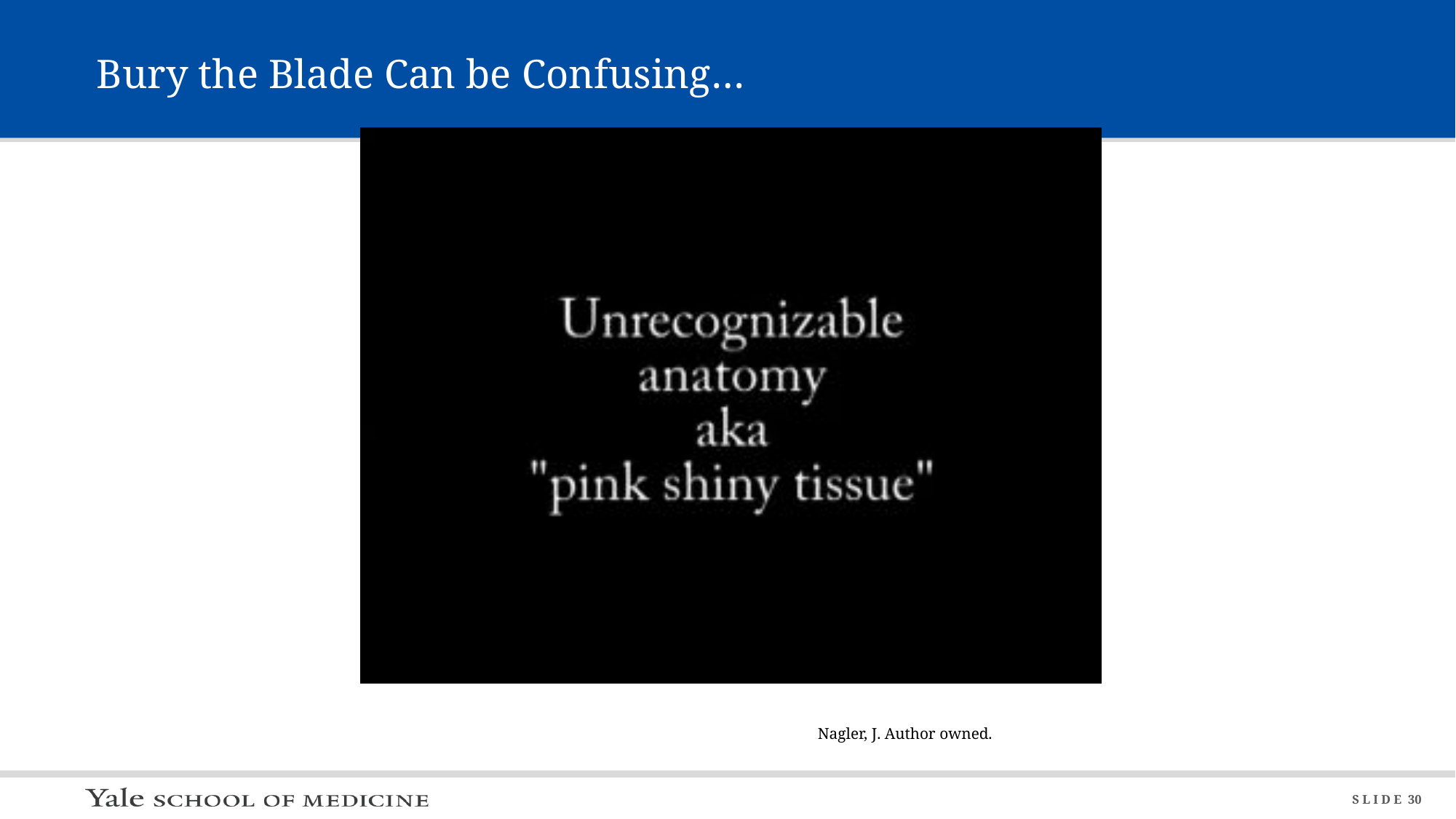

# Bury the Blade Can be Confusing…
Nagler, J. Author owned.

## Slide 31
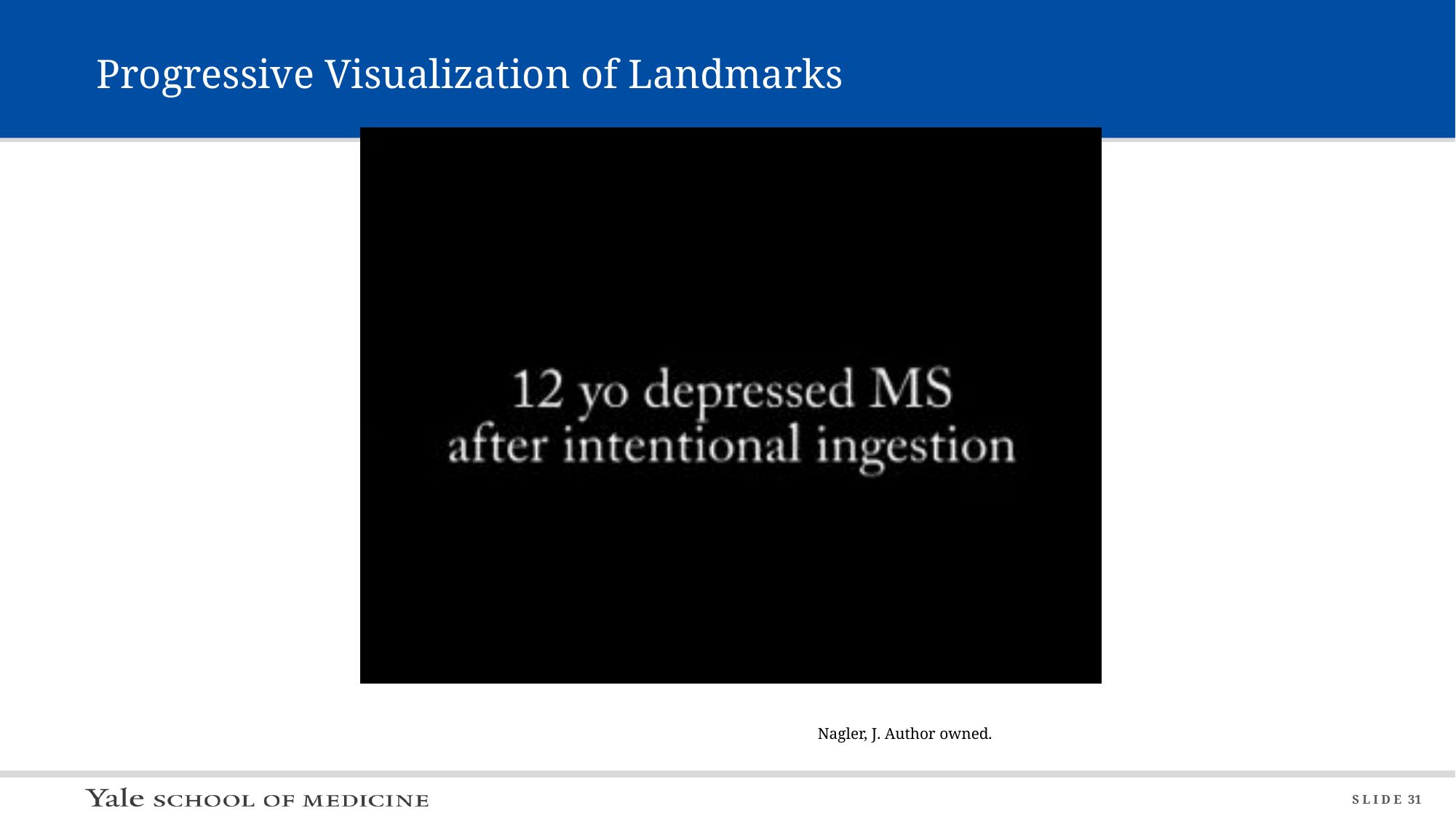

# Progressive Visualization of Landmarks
Nagler, J. Author owned.

## Slide 32
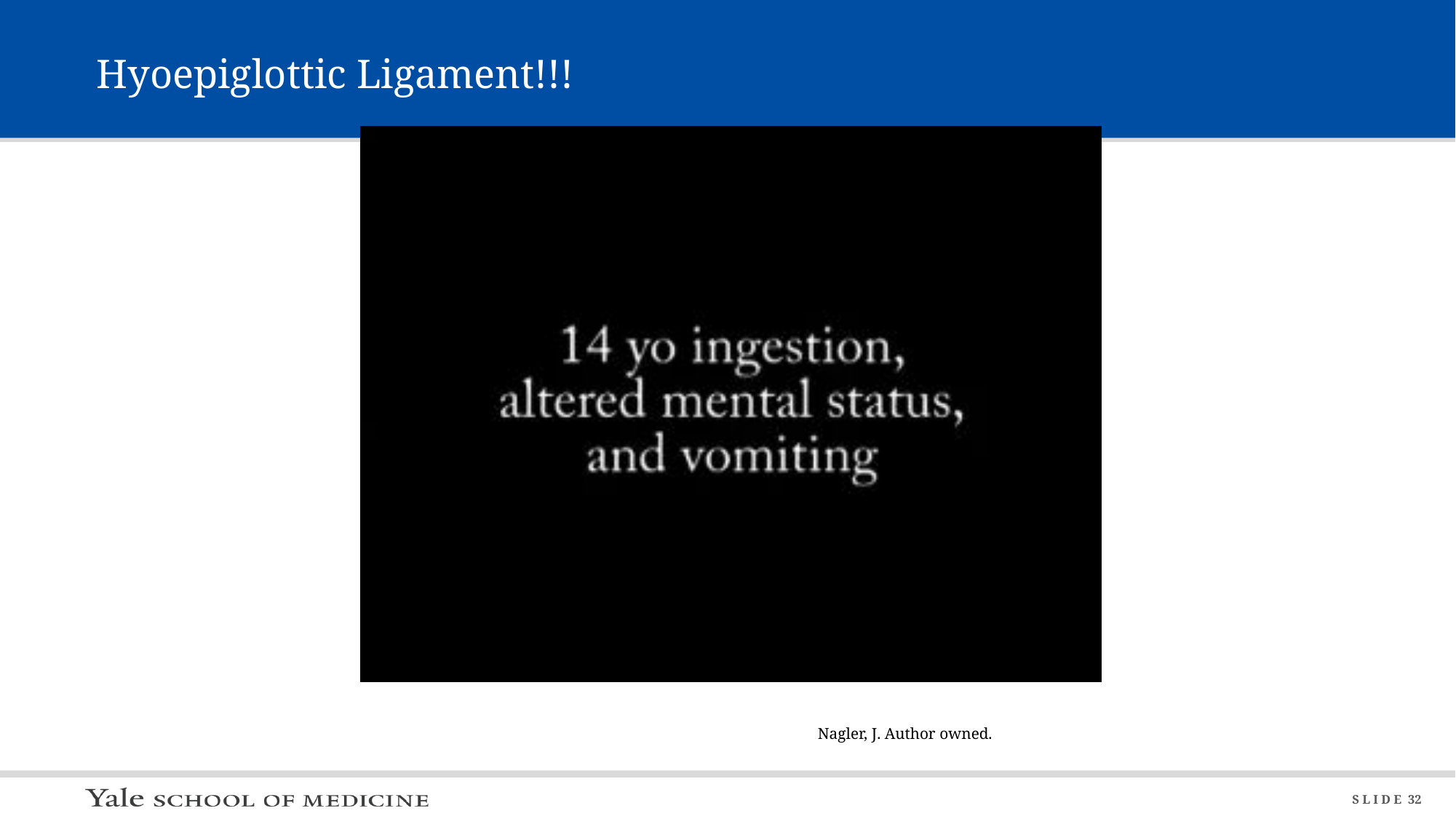

# Hyoepiglottic Ligament!!!
Nagler, J. Author owned.

## Slide 33
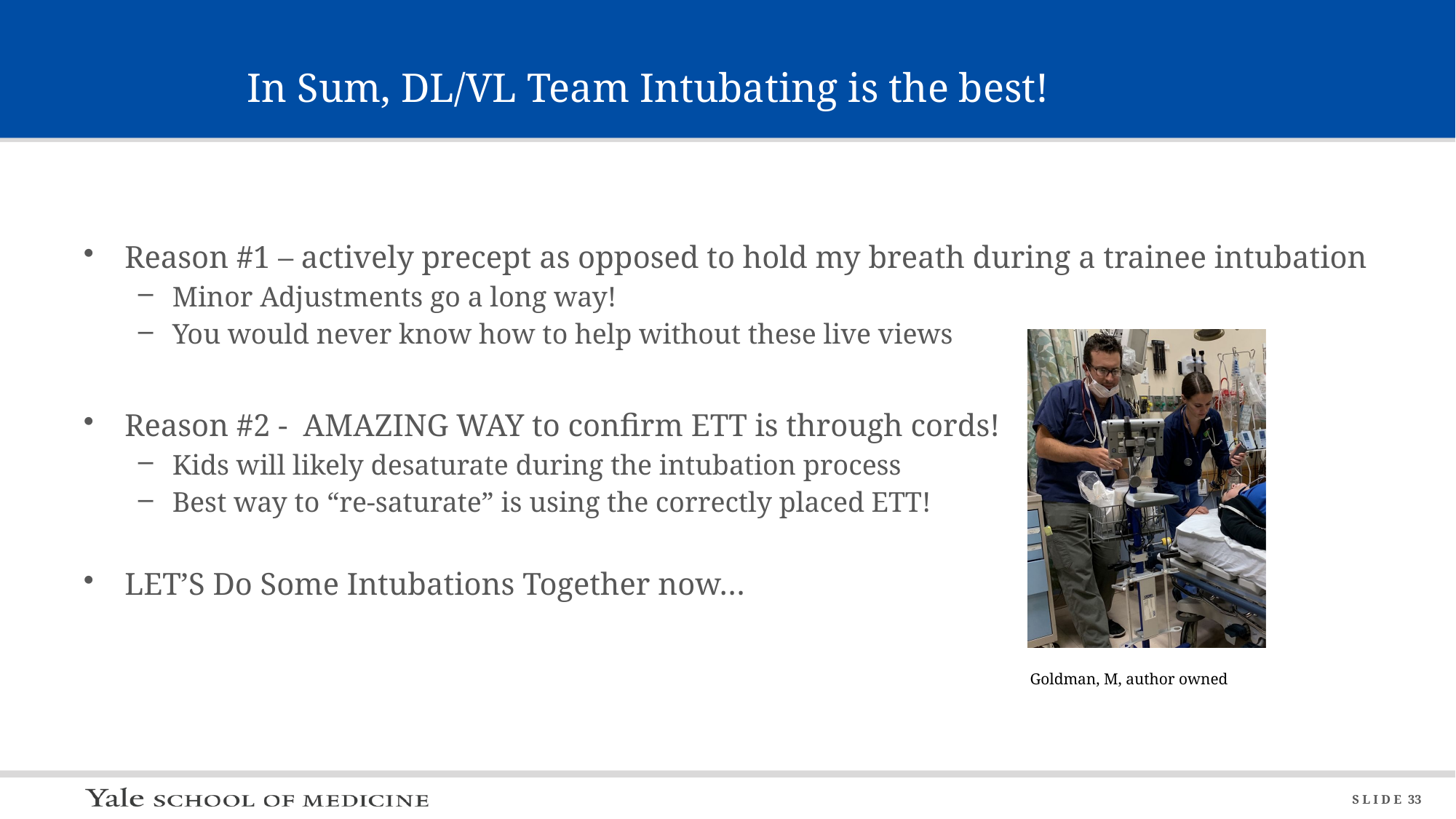

# In Sum, DL/VL Team Intubating is the best!
Reason #1 – actively precept as opposed to hold my breath during a trainee intubation
Minor Adjustments go a long way!
You would never know how to help without these live views
Reason #2 - AMAZING WAY to confirm ETT is through cords!
Kids will likely desaturate during the intubation process
Best way to “re-saturate” is using the correctly placed ETT!
LET’S Do Some Intubations Together now…
Goldman, M, author owned

## Slide 34
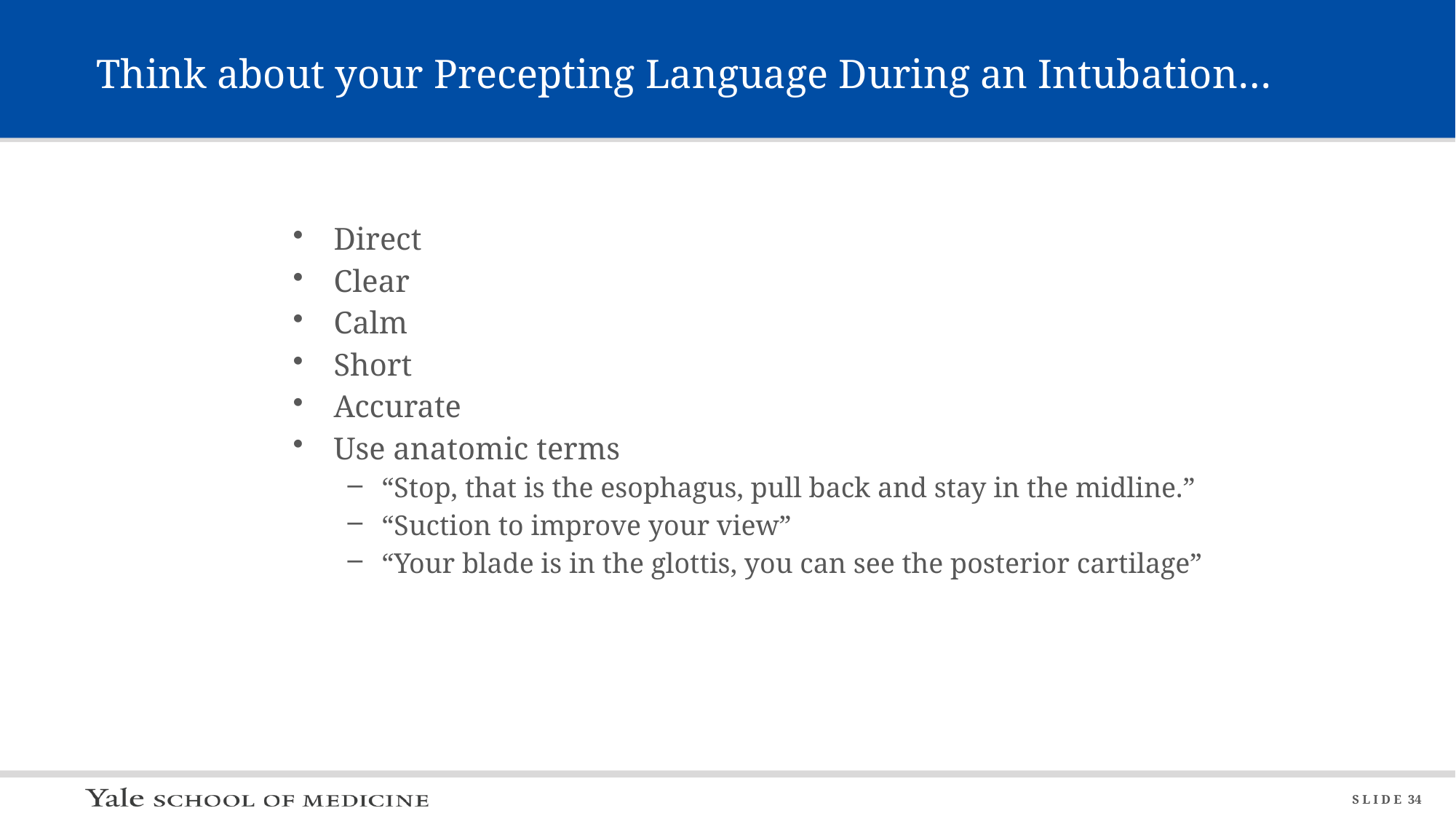

# Think about your Precepting Language During an Intubation…
Direct
Clear
Calm
Short
Accurate
Use anatomic terms
“Stop, that is the esophagus, pull back and stay in the midline.”
“Suction to improve your view”
“Your blade is in the glottis, you can see the posterior cartilage”

## Slide 35
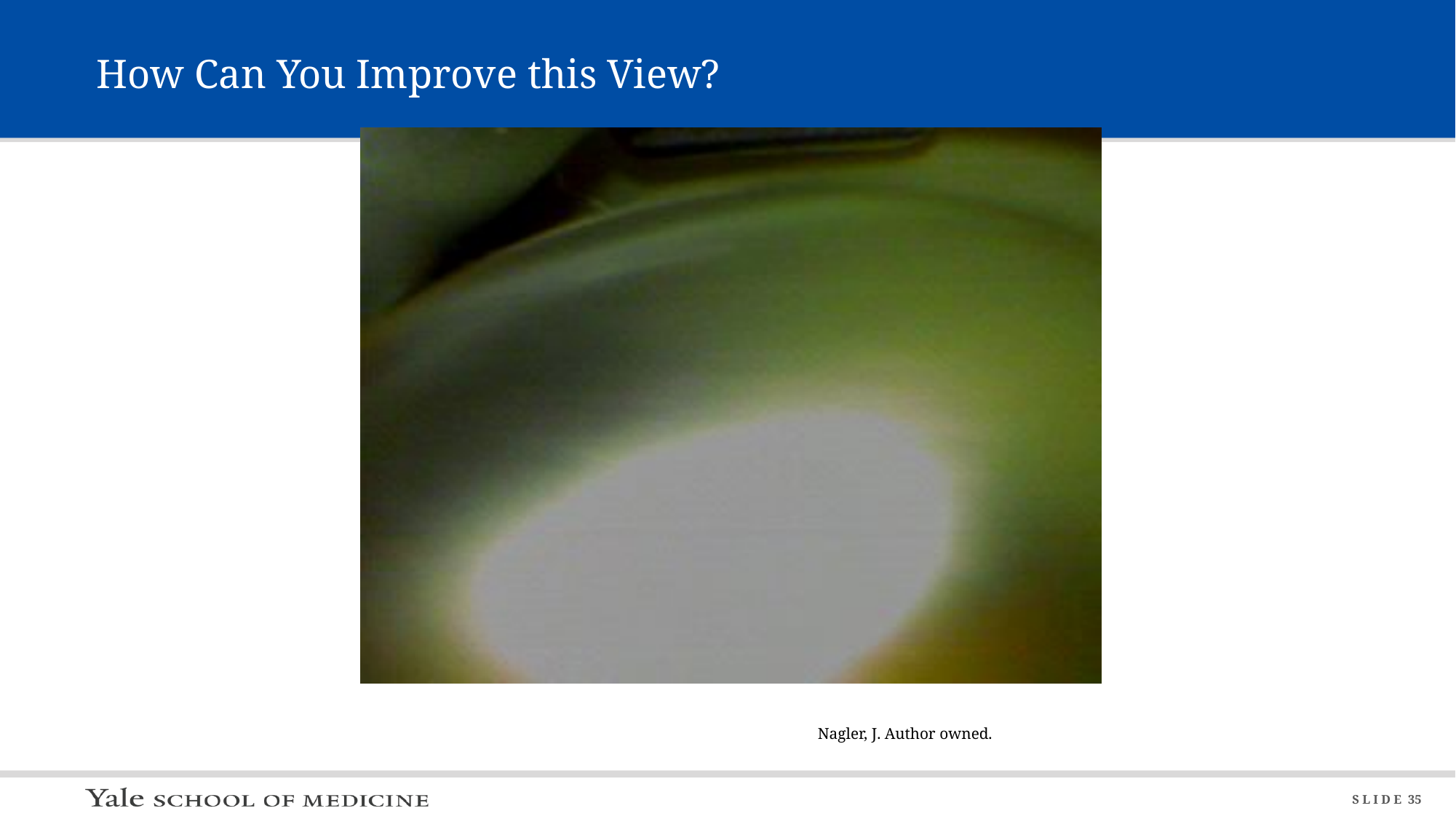

# How Can You Improve this View?
Nagler, J. Author owned.

## Slide 36
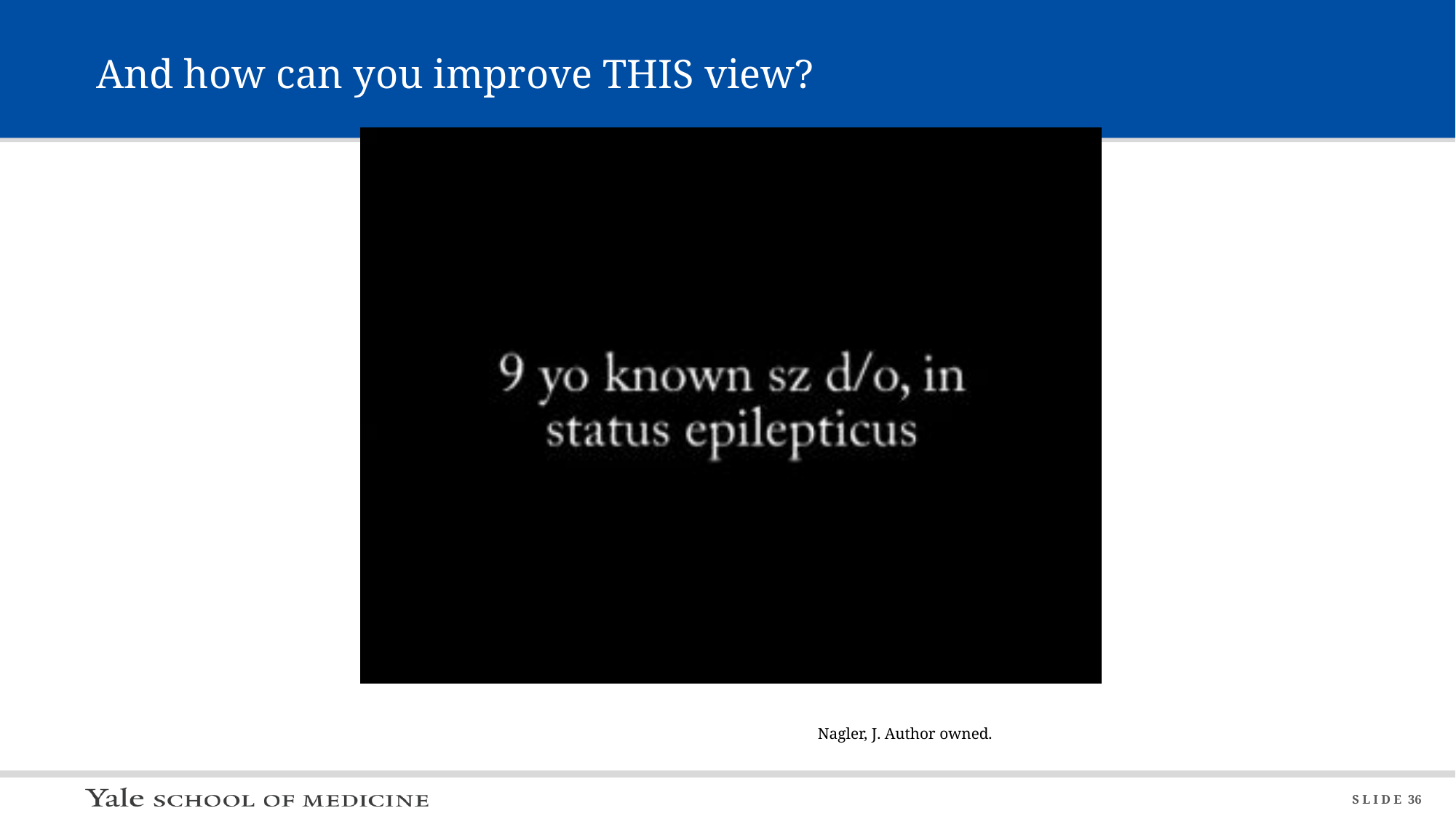

# And how can you improve THIS view?
Nagler, J. Author owned.

## Slide 37
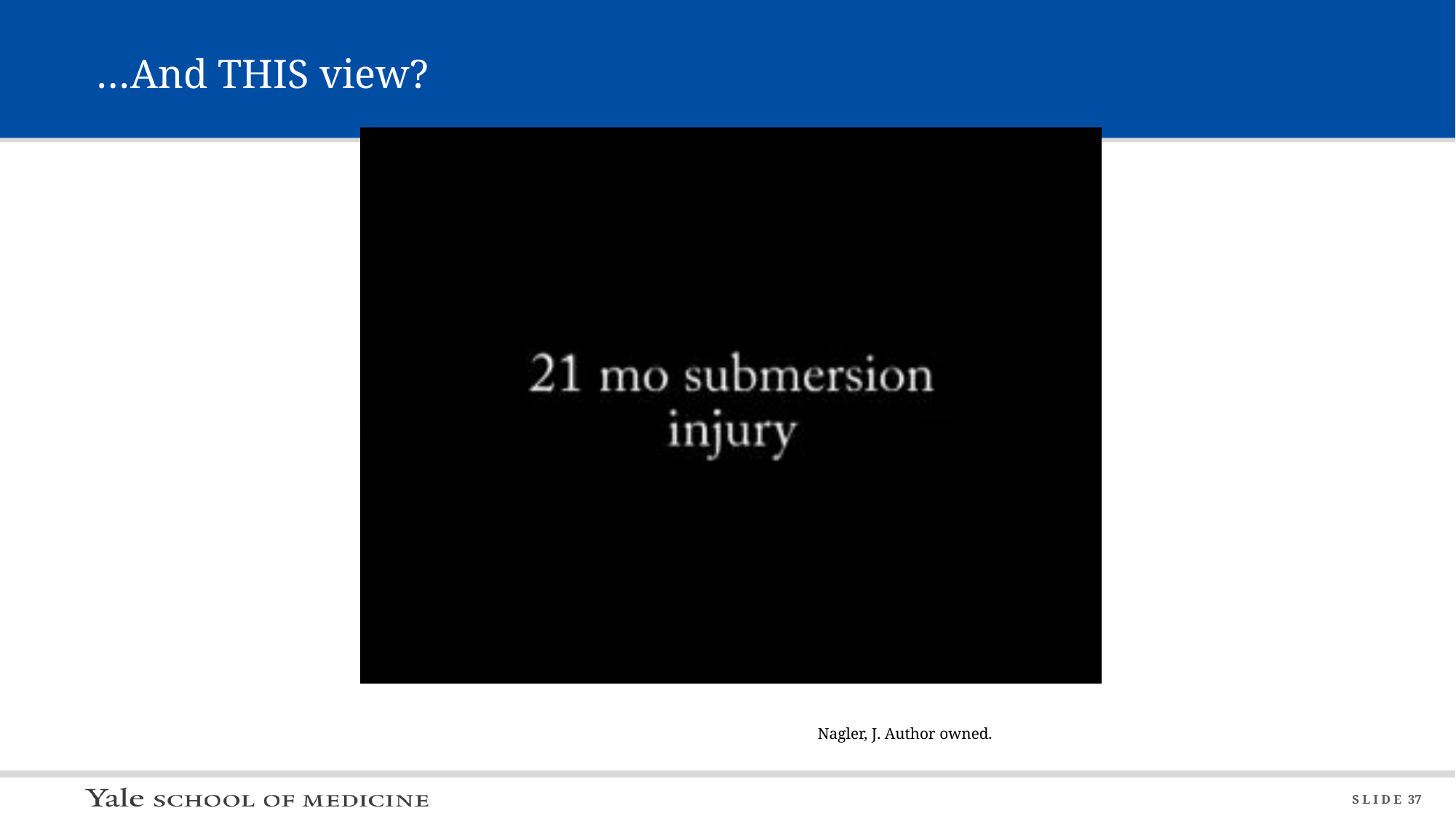

# …And THIS view?
Nagler, J. Author owned.

## Slide 38
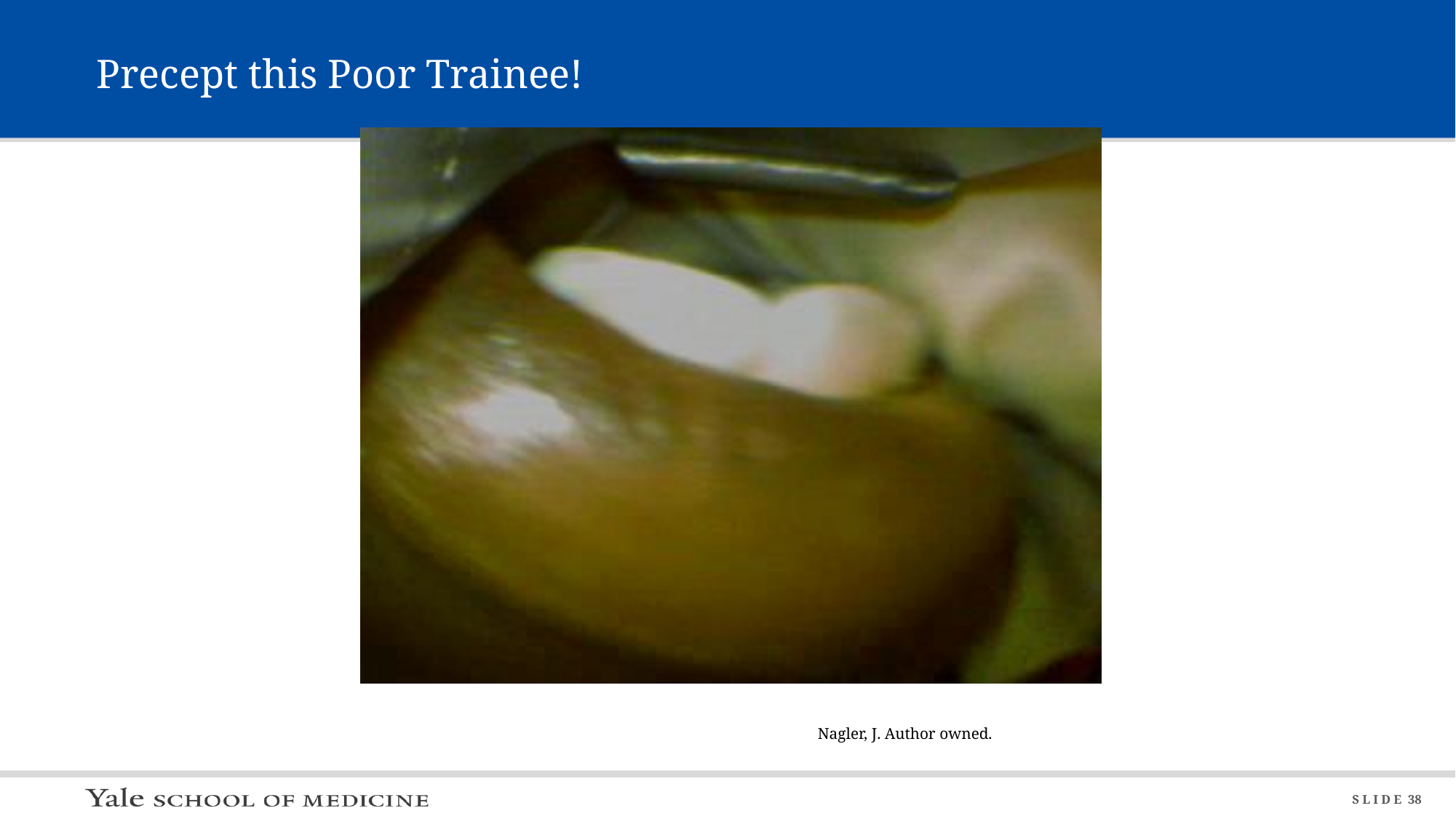

# Precept this Poor Trainee!
Nagler, J. Author owned.

## Slide 39
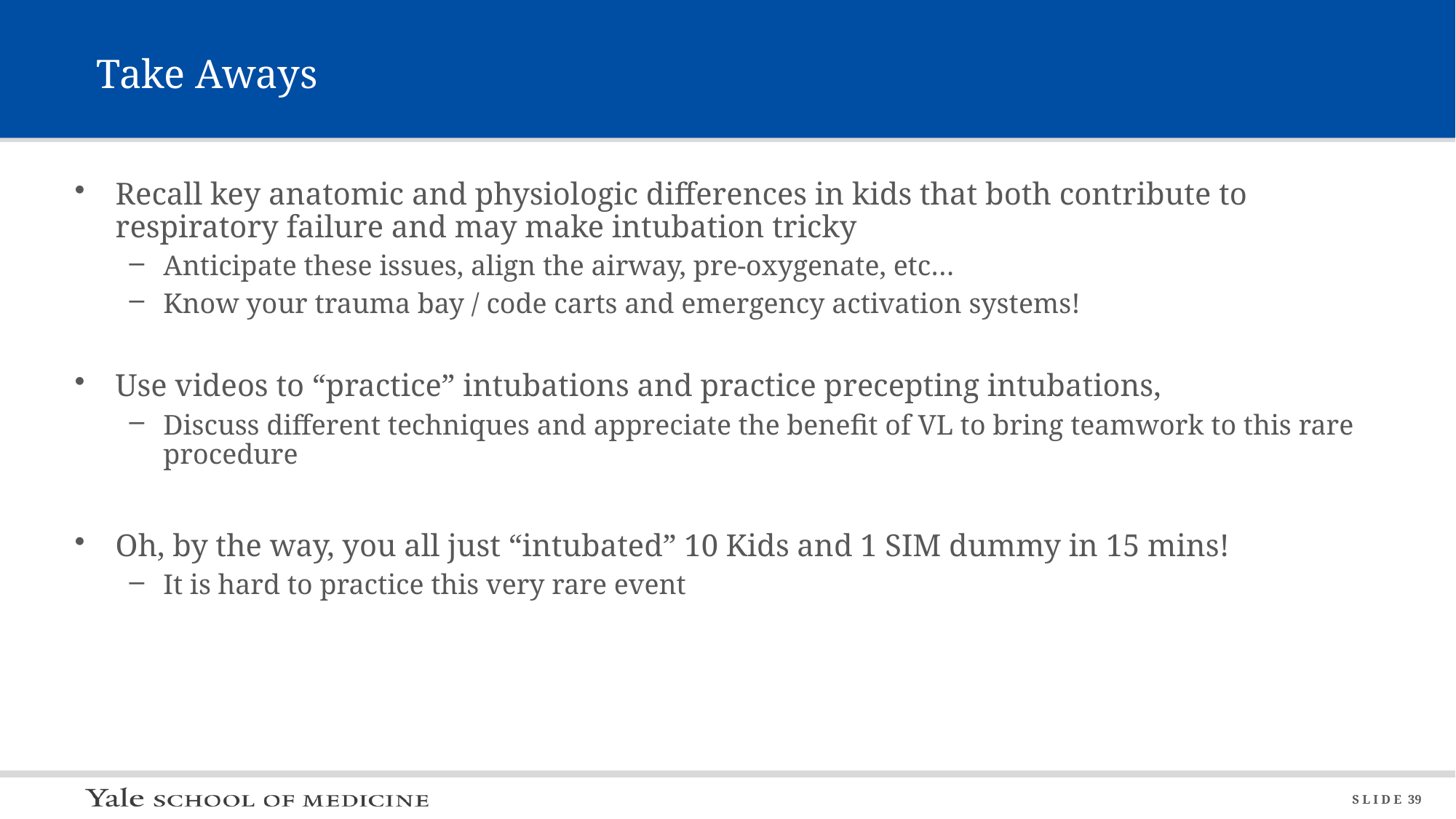

# Take Aways
Recall key anatomic and physiologic differences in kids that both contribute to respiratory failure and may make intubation tricky
Anticipate these issues, align the airway, pre-oxygenate, etc…
Know your trauma bay / code carts and emergency activation systems!
Use videos to “practice” intubations and practice precepting intubations,
Discuss different techniques and appreciate the benefit of VL to bring teamwork to this rare procedure
Oh, by the way, you all just “intubated” 10 Kids and 1 SIM dummy in 15 mins!
It is hard to practice this very rare event
