# Supplementary material for: Advanced Pediatric Emergency Airway Management: A Multimodality Curriculum Addressing a Rare but Critical Procedure
Source: MedEdPORTAL. 2020 Sep 4;16:10962. doi: 10.15766/mep_2374-8265.10962 (PMC7473185; doi:10.15766/mep_2374-8265.10962)
Supplement: Supplementary file 1 — Course Syllabus.docxStation 1 Didactic Videos.pptxStation 2 Needle Cricothyrotomy Cognitive Aid.pptxIntubation Teaching Feedback Rubrics.docxStation 3 Simulation.docxStation 4 Simulation.docxCurriculum Evaluation.docx [file mep_2374-8265.10962-s001.zip › C. Station 2 Needle Cricothyrotomy Cognitive Aid.pptx]

## Slide 1
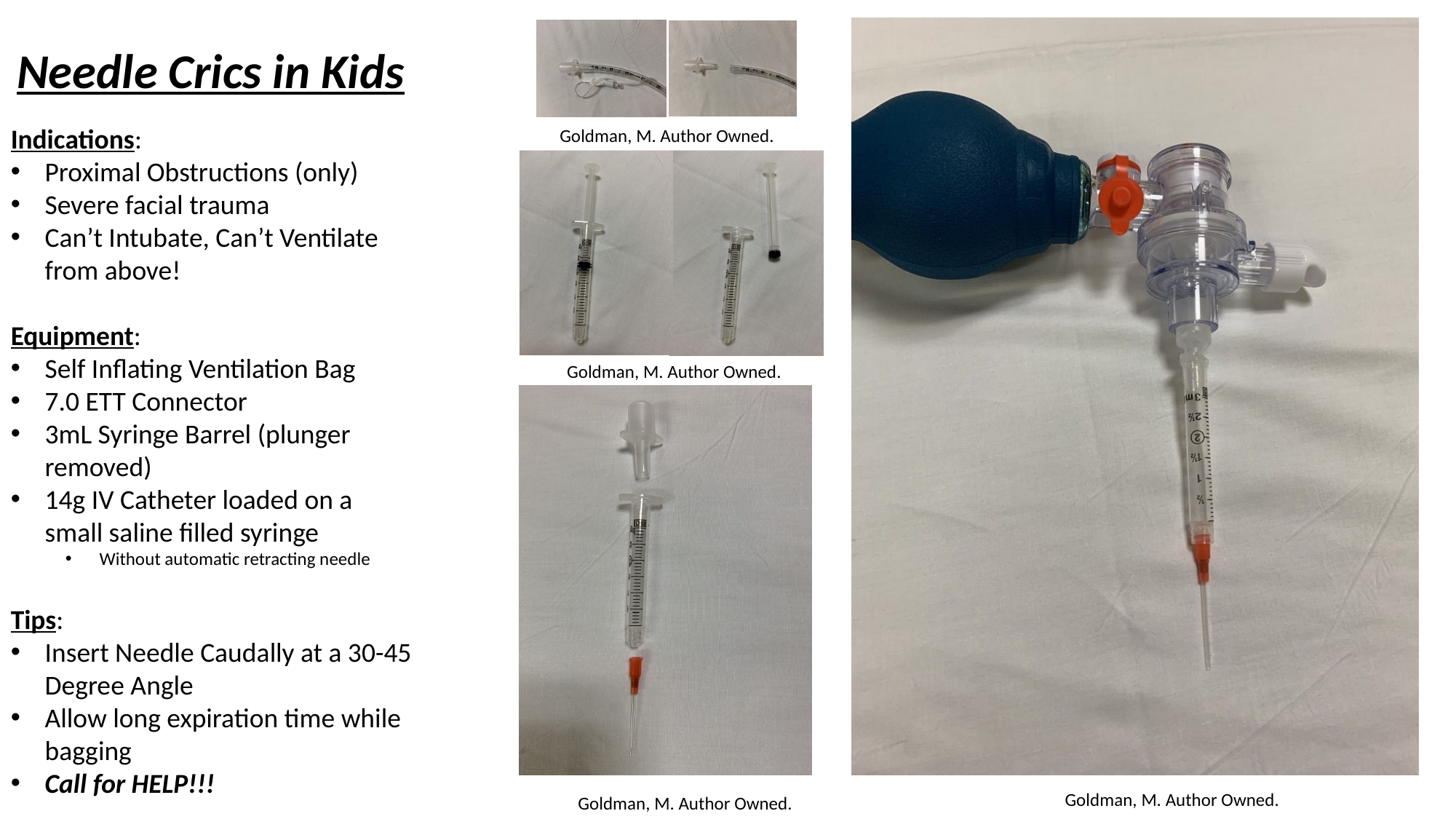

Needle Crics in Kids
Indications:
Proximal Obstructions (only)
Severe facial trauma
Can’t Intubate, Can’t Ventilate from above!
Equipment:
Self Inflating Ventilation Bag
7.0 ETT Connector
3mL Syringe Barrel (plunger removed)
14g IV Catheter loaded on a small saline filled syringe
Without automatic retracting needle
Tips:
Insert Needle Caudally at a 30-45 Degree Angle
Allow long expiration time while bagging
Call for HELP!!!
Goldman, M. Author Owned.
Goldman, M. Author Owned.
Goldman, M. Author Owned.
Goldman, M. Author Owned.

## Slide 2
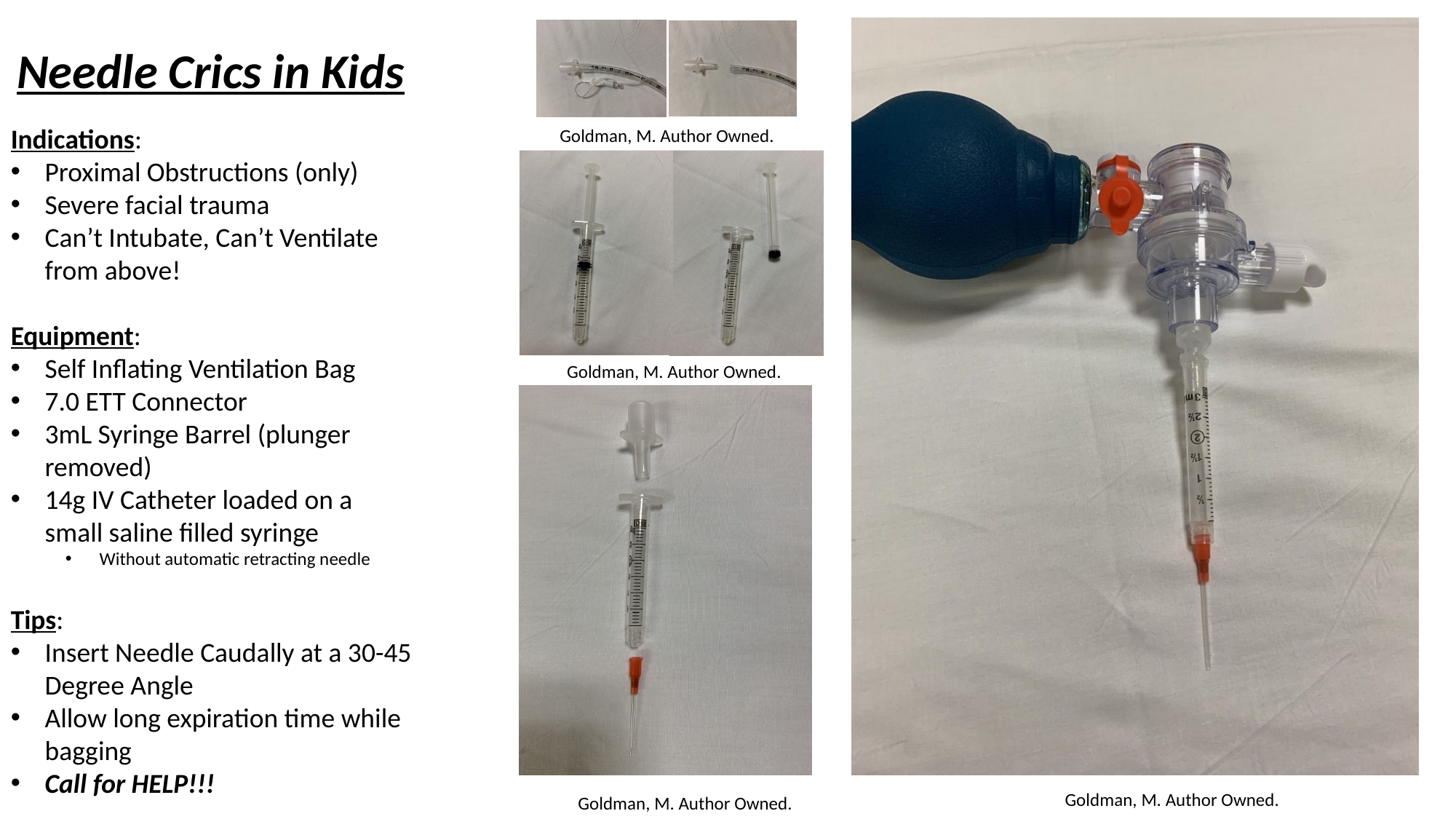

Needle Crics in Kids
Indications:
Proximal Obstructions (only)
Severe facial trauma
Can’t Intubate, Can’t Ventilate from above!
Equipment:
Self Inflating Ventilation Bag
7.0 ETT Connector
3mL Syringe Barrel (plunger removed)
14g IV Catheter loaded on a small saline filled syringe
Without automatic retracting needle
Tips:
Insert Needle Caudally at a 30-45 Degree Angle
Allow long expiration time while bagging
Call for HELP!!!
Goldman, M. Author Owned.
Goldman, M. Author Owned.
Goldman, M. Author Owned.
Goldman, M. Author Owned.
