# Supplementary material for: Advanced Pediatric Emergency Airway Management: A Multimodality Curriculum Addressing a Rare but Critical Procedure
Source: MedEdPORTAL. 2020 Sep 4;16:10962. doi: 10.15766/mep_2374-8265.10962 (PMC7473185; doi:10.15766/mep_2374-8265.10962)
Supplement: Supplementary file 1 — Course Syllabus.docxStation 1 Didactic Videos.pptxStation 2 Needle Cricothyrotomy Cognitive Aid.pptxIntubation Teaching Feedback Rubrics.docxStation 3 Simulation.docxStation 4 Simulation.docxCurriculum Evaluation.docx [file mep_2374-8265.10962-s001.zip › D. Intubation Teaching Feedback Rubrics.docx]

**Examples of Direct Observation / Coaching Pediatric Intubation Rubric -**

These published intubation rubrics / checklists can provide facilitators a framework for their feedback and coaching when working with learners either at the deliberate practice station (2) or the two simulations (stations 3 and 4).

1. *As adapted from Abu-Sultaneh et al’s “Improving Simulated Pediatric Airway Management in Community Emergency Departments Using a Collaborative Program with a Pediatric Academic Medical Center” – Respiratory Care - 2019*

| Item | Y/N | Comments |
| --- | --- | --- |
| Equipment’ selection:   1. ETT Size 2. Cuffed vs non-cuffed 3. Cuff checked 4. Stylet 5. Blade size 6. Laryngoscope check 7. Suction catheter 8. Bag and mask |  |  |
| 1. Backup Plan Articulated 2. Time out performed |  |  |
| 1. Patient’s head positioned properly |  |  |
| 1. Appropriate bagging technique |  |  |
| 1. Laryngoscope blade inserted properly |  |  |
| 1. ETT inserted to appropriate depth |  |  |
| 1. Stylet removed |  |  |
| 1. ETT placement verified using ETCO2 AND chest auscultation |  |  |
| 1. CXR ordered for confirmation |  |  |

1. *As adapted from Johnston et al. “*Neonatal Intubation Competency Assessment Tool: Development and Validation.” *Academic Pediatrics. 2019.*

|  | ***Done Correctly*** | ***Done Incorrectly or Not Done*** | ***Not needed or applicable*** |
| --- | --- | --- | --- |
|  | ***1Pts*** | ***0pts*** | ***N/A*** |
| **Verbalizes the *indications* for procedure?** |  |  |  |
| **Verbalizes the risks and/ or c*ontraindications* for procedure?** |  |  |  |
| **Verbalizes appropriate *planning* for the procedure (identifies risk factors for difficult intubation, including patient history, anatomic features, and physiologic instability)?** |  |  |  |
| **Verbalizes AND demonstrates appropriate *preparation of equipment* for procedure (including standard intubation equipment, as well as adjunct devices if concern for difficult airway)?** |  |  |  |
| **Requests that *appropriate personnel are present* for procedure, including team leader (separate from airway provider) nursing, respiratory therapy, and potentially ENT/ anesthesia if concerns for difficult airway** |  |  |  |
| **Obtains/ verifies consent for elective intubation, identifies patient and performs a time-out?** |  |  |  |
| **1. Chooses appropriate size and type of ET tube (if utilized, inserts stylet appropriately)** |  |  |  |
| 1. **Performs equipment check (ensures that laryngoscope, suction, ET tube (with additional tubes/ sizes available), CO2 detector, BVM and monitoring devices), are assembled, available & in working order before commencing procedure** |  |  |  |
| 1. **Demonstrates the appropriate use of sedation/ pre-medication (including possible use of paralytics/ atropine)** |  |  |  |
| 1. **Performs preoxygenation (method depends on area of practice/ patient population)** |  |  |  |
| 1. **Demonstrates appropriate positioning of patient (utilizing proper bed height, head position, shoulder roll, and c-spine precautions (if appropriate))** |  |  |  |
| 1. **Employs appropriate technique to open mouth prior to inserting blade** |  |  |  |
| 1. **Demonstrates smooth blade insertion using left hand (must use left hand)** |  |  |  |
| 1. **Demonstrates appropriate technique to lift handle of laryngoscope forward (does not pivot handle, and utilizes smooth movement**   **9. Demonstrates appropriate technique to visualize vocal cords (understands how to manipulate blade, requests other maneuvers to see cords if necessary) and states when they have achieved view of glottis** |  |  |  |
| **10. Demonstrates appropriate use of suction (if needed)** |  |  |  |
| **11. Demonstrates appropriate insertion of ET tube using one smooth motion** |  |  |  |
| **12. Demonstrates insertion of ET tube to appropriate depth (checks position at level of cords and lip), and verbalizes final position of ETT at lip** |  |  |  |
| **13. Employs appropriate techniques to confirm correct placement of ET tube by primary (auscultation or confirmation of bilateral chest rise) and secondary (qualitative or quantitative EtCO2 detection) methods** |  |  |  |
| **Procedure successful? Success= *ET tube was placed through the cords (verified using VL OR independent instructor DL OR bilateral chest rise with PPV) on first attempt with <30 seconds of non-ventilated time*** |  |  |  |
| **Demonstrates ability to troubleshoot during procedure when prompted by facilitator (if necessary)?** Please describe*: __________________________________* |  |  |  |
| **If complications encountered, please describe:**  *__________________________________* |  |  |  |
| **Performs appropriate aftercare (Secures ET tube, confirms placement with CXR, selects ventilator settings)** |  |  |  |
