# Supplementary material for: Advanced Pediatric Emergency Airway Management: A Multimodality Curriculum Addressing a Rare but Critical Procedure
Source: MedEdPORTAL. 2020 Sep 4;16:10962. doi: 10.15766/mep_2374-8265.10962 (PMC7473185; doi:10.15766/mep_2374-8265.10962)
Supplement: Supplementary file 1 — Course Syllabus.docxStation 1 Didactic Videos.pptxStation 2 Needle Cricothyrotomy Cognitive Aid.pptxIntubation Teaching Feedback Rubrics.docxStation 3 Simulation.docxStation 4 Simulation.docxCurriculum Evaluation.docx [file mep_2374-8265.10962-s001.zip › E. Station 3 Simulation.docx]

| **Appendix E: MedEdPORTAL Simulation Case Template**  **SIMULATION CASE TITLE: Impending respiratory failure in a child**  **AUTHORS:** Michael P. Goldman, MD^1^, Ambika Bhatnagar, MBBS^2^, Joshua Nagler, MD, MHPEd^3^, Marc Auerbach, MD, MSc^4^ | |
| --- | --- |
| **PATIENT NAME: Sarah**  **PATIENT AGE: 2 y.o.**  **CHIEF COMPLAINT: Seizure** | |
|  | |
| **Brief narrative description of case** | This case describes EMS bringing a postictal 2 year-old female patient who started seizing at daycare 20 minutes ago. On presentation, the patient has mild stertor which improves with airway opening maneuvers and supplemental oxygen. However, she starts seizing after 2 minutes with excessive frothing in the mouth and progressive respiratory depression both from the seizure and benzodiazepime abortive therapies. The learner’s goals are to recognize impending respiratory failure in refractory status epilepticus, create a team-based plan for rapid sequence intubation and safely intubate via direct laryngoscopy technique but using a video laryngoscope so the airway assistant can provide immediate feedback based on what they observe through the video output. |
| **Primary Learning Objectives** | - Recognize at least two signs and symptoms of impending respiratory failure in the pediatric patient - Verbalize and implement a pediatric rapid sequence intubation plan considering the risks and benefits of different pre-medications, induction agents and neuromuscular blocking agents - Use a peri-intubation checklist^#^ - To optimize first pass success, perform “team-based intubation” using direct laryngoscopy technique with a video laryngoscope so the team leader / airway preceptor can provide immediate procedural feedback as indicated. - Experience the advantage of team-based intubation to confirm proper endotracheal tube placement - Improve teamwork and communication skills:   - Effectively pre-brief at the start of the scenario, assign roles, strategize, recap during the scenario and debrief after the scenario.   - Communicate with team members using closed loop communication, call-outs and check backs. |
| **Critical Actions** | - Demonstrate initial assessment of the child including prompt primary and secondary surveys followed by a focused history. - Use appropriate cardiorespiratory, oxygen saturation, end tidal CO2, and temperature monitoring. - Identify and manage upper airway obstruction with basic airway maneuvers, airway adjuncts, suction, supplemental oxygen and bag valve mask. - Choose appropriate abortive anti-epileptics while considering and preparing for the complication of further respiratory depression. - Recognize the need to intubate to manage respiratory failure. - Choose appropriate RSI medications and dosing by discussing among team members and/or by using cognitive aids. - Articulate a back-up plan if intubation fails. - Ensure appropriate positioning of airway, size of equipment, proper location and depth of endotracheal tube. |
| **Learner Preparation** | 2-year-old, Sarah, who EMS believes has a history of seizures, was found seizing at daycare. The daycare personnel gave her rectal Diastat 10 minutes ago and then EMS gave an additional dose of Intramuscular Midazolam for persisting movements now 3 mins ago.  Learners will get 60 seconds to prepare, assign roles (e.g. team leader, airway manager, etcetera) and then it will be announced, “the patient has arrived.” |

| Initial Presentation | | | |
| --- | --- | --- | --- |
| **Initial vital signs** | HR-160 bpm(sinus rhythm)  BP-100/50 mm Hg  RR-25 bpm, shallow  SpO2- 95%  Temp-38.5 ° C | | |
| **Overall Appearance** | Patient is not seizing. She is not crying and has an altered level of consciousness. | | |
| **Actors and roles in the room at case start** | Patient’s mother is present in the room and EMS has left. Mother played by an assistant or faculty will give the history of the patient. | | |
| **HPI** | Mother will tell the learners that, “She is no longer seizing and she always gets sleepy like this after seizure.” She is not concerned as her daughter has had many prior seizures that resolve with Midazolam.  When asked mother will give the following information when specifically asked:   - Initial seizure started 20 minutes ago. - School RN placed her on her side and gave diastat after 10 minutes. - EMS arrived and gave a dose of IM Midazolam as she was still seizing. - She had fever up to 102 and had flu-like symptoms this week, but did not have a fever this morning so went to school. - She has otherwise been drinking well and taking seizure meds normally. - Her typical seizures last 3-5 minutes and mom gives diastat if > 10 minutes. - Her longest seizure was 40 minutes. - Mom has never been told that the patient has a “difficult airway” from all the visits to the operating room for Ventriculoperitoneal Shunt revisions. - The patient’s vaccinations are up to date. | | |
| **Past Medical/Surgical History** | **Medications** | **Allergies** | **Family History** |
| Ex-27 week premie born with Hypoxic Ischemic Encephalopathy (HIE), had a prolonged stay in NICU. Now has a seizure disorder and some developmental delay | Keppra, twice daily | none | none |
| **Physical Examination** | | | |
| **General** | Patient is not seizing. She is not crying and has altered level of consciousness. She has mild stertor /upper airway congestion noises. | | |
| **HEENT** | Pupils 6 mm and minimally reactive bilaterally. | | |
| **Neck** | Normal. | | |
| **Lungs** | Coarse breath sound bilaterally. | | |
| **Cardiovascular** | Tachycardia. | | |
| **Abdomen** | Normal. | | |
| **Neurological** | Altered level of consciousness. | | |
| **Skin** | No bruising or trauma findings. | | |
| **GU** | Normal. | | |
| **Psychiatric** | NA | | |

| Instructor Notes - Changes and CASE Branch Points | | |
| --- | --- | --- |
| **Intervention / Time point** | **Change in Case** | **Additional Information** |
| Jaw-thrust, Chin Lift and/or oxygen supplementation provided | Oxygen saturation will increase from 95% to 98%. | Mom states that she is no longer seizing and that she “always gets sleepy like this after seizure” |
| Participant requests finger stick blood glucose. | Glucose level is 145. |  |
| 2-5 minutes | Patient starts seizing with frothing in mouth. Heart rate increases to 180 from 160 and oxygen saturation decreases from 95% to 85% over 2 minutes. | Mother starts advocating for more benzos, stating that “always works.” |
| If phenobarbital, benzodiazepenes or propofol is used | The seizure stops. But this causes a decrease in respiratory rate to 10 and oxygen saturation to 85% over 2 minutes. | Common Med Dosing:  Lorazepam- 0.05-0.1 mg/kg IV/IO  Midazolam- 0.1 – 0.2 mg/kg IV/IO  Midazolam – 0.2 mg/kg Intranasal |
| If fosphenytoin or keppra used | The seizure stops. But the patient vomits and oxygen saturation decreases to 80%. | Fosphenytoin – 15-20 PE/kg IVIO  Keppra – 50mg/kg IV/IO |
| During Intubation | During intubation, allow 15 seconds before saturations start to fall. | Atropine – 0.02 mg/kg IV/IO  Etomidate – 0.3mg/kg IV/IO  Ketamine – 1.5-2 mg/kg IV/IO |
| During intubation, if a paralytic is not used | Patient vomits. | Rocuronium – 1-1.2mg/kg IV/IO  Succinylcholine – 1 - 1.5mg/kg IV/IO |
| Post intubation | Vitals  HR 140  BP 100/50 (85/45 if propofol used for induction)  SpO2: 100%  RR 20 |  |
| Notify Pediatric Critical Care Team / transfer team | End simulation |  |

**Ideal Scenario Flow**

The learners enter the room to find a drowsy 2-year-old female patient, brought by EMS after seizing at school. Learners immediately place the patient on bedside monitors and recognize that the patient is hypoxic with some mild to moderate upper airway obstruction signs (i.e. stertor). The team suctions the airway and repositions the patient to improve air entry by performing maneuvers like head tilt-chin lift, jaw thrust, and/or by using a shoulder roll. The patient’s respiratory distress improves when these maneuvers plus supplemental oxygen is provided. The learners then begin to obtain an appropriate history from the mother and progress through the primary and secondary surveys to examine the patient. After 2 minutes, the patient starts seizing with a declining oxygen saturation. To control these ongoing seizures, learners order Benzodiazepines (BDZs). After administrating BDZs, they recognize the deteriorating respiratory status and start bagging the patient while articulating the plan to intubate. Appropriate RSI medications and medication doses are ordered along with the correct sized endotracheal tubes and layngoscopy blades. Learners also formulate a backup plan if they fail to intubate. After successful intubation, learners confirm correct placement of the tube through in-line End Tidal CO2 monitoring, portable chest x-ray, and listening to the lung sounds bilaterally. The case ends with notification to the critical care team or critical care transfer team.

*Potential Branch Points*

- While the case nearly forces the learners to intubate the patient, it is vital for learners to practice and use their basic airway maneuvers, which in pediatrics includes a Jaw-thrust, Chin Lift and emphasizes proper airway alignment with a shoulder roll. Applying oxygen is also of course a critical first step.
- A finger stick blood glucose check is a key step in the management of status epilepticus. It should be requested. But the value should return as normal so the learners do not go off task and focus on the differential diagnosis of altered mental status. Rather, the emphasis of the simulation is to manage the respiratory complications of status epilepticus.
- If phenobarbital, benzodiazepenes or propofol is used, the case focuses the learners on the emergency airway management as these medications are known respiratory depressants.
- If phosphenytoin or keppra used, the seizure stops but another indication for intubation arises given the compromised airway protective reflexes from a neurologic emergency.
- During Intubation, regardless of how flawless it goes, hypoxia is common as children desaturate quickly. Mimicking this phenomenon in the simulation is important to bring palbable stress to the scene.
- During intubation, if a paralytic is not used, the patient vomits. The use of a paralytic has been demonstrated to improve first pass success rate during the intubation of children. This will likely come up during the debrief as many will defer paralytics out of caution for inducing a situation where the patient cannot be intubated or ventilated.

**Anticipated Management Mistakes**

1. Advanced Airway Management starts with Basic Airway Management – While our curriculum focuses a lot on invasive airway management skills, we quickly realized the importance of emphasizing the critical importance of basic airway management skills at all four learning stations. Specifically, we advocate for the application of a shoulder roll to assist with airway patency and excellent bag mask ventilation, especially having two hands on the mask and an additional provider squeezing the bag. When learners did not pay attention to this skills in the SIM, we found that worsening the hypoxia moved them to address this with their basic airway management skills.
2. Sizes and Dosing – Again and again, our leanrers were flustered with finding and organizing the proper sized equipment and dosing of medications. We had to stop a couple of SIMs briefly to remind learners that their PALS cards or mobile phones have applications that can quickly help relieve this cognitive burden and that they should use these cognitive aids both in simulations and in real life, especially during the preparation phase (if they have such time). We also briefly reviewed known equations but emphasize “knowing your trauma bays” and your resources before the stressful case is in front of you.
3. Failure to Recognize Worsening Respiratory Status after dosing Abortive Seizure Medications – Most of the acute, abortive anti-epileptics in status epilepticus management are respiratory depressants. Some of our learners were astute to this complication and ordered varied anti-epileptics such as Keppra and Fos-Phenytoin. As such, to push the scenario back to its focus, airway management, we made the child vomit which forces the team towards intubation for airway protection.
4. Paralytics – Conventional teaching cautions the airway manager to avoid paralytics unless one is confident they can place the endotracheal tube. More recent literature suggests that first pass success in children is augmented by use of a paralytic. We encountered physicians, especially in the community hospital setting who refused to use a paralytic. As such, we made the patient vomit during the intubation so as to place a bookmark on the case for review this management decision during the debrief. Failure to use a paralytic also places the patient at risk for layngospasm.
5. Rocuronium versus Succinylcholine – In our curriculum, we emphasize the use of a paralytic that the intubation team is most familiar with, including nursing staff. We do like to remind folks of some of the pediatric specific considerations when using Succinylcholine. Essentially, the fear is that the type of kids that get intubated are often those that are chronically ill or in a bad trauma. Likely very small but a proportion of these chronically ill children may have diagnosed or undiagnosed myopathies. The trauma patient is at risk for rhabdomyolysis. As such, it may be best to avoid Succinylcholine in all kids especially as the onset of action for Rocuronium is comparable to Succinylcholine and there is an available antidote (i.e. Sugammadex). Of note, there is also a black box warning against Succinylcholine in children, with the caveat that the drug may be used in the emergency setting.
6. Atropine – There remains only one intubation premedication in pediatrics. As per PALS guidelines, Atropine is recommended in children less than 1 and considered in children less than 2 to address and prevent layngoscopy induced vagal mediated bradycardia. Learners often did not use atropine in this SIM but almost all asked about it after during the debrief.
7. Laryngoscopy with finesse, not force – Pediatric laryngoscopy, in contrast to some adult cases, requires little force and a lot of finesse. We have experienced many of our providers more used to caring for adults use a lot of muscle when trying to place the endotracheal tube. This has the potential for orophaygeal and airway trauma which can subsequently make for a challenging intubation. We have discussed this concept many times during the debriefs. Subtle dissection for the landmarks is emphasized in Station 1 as we review the real patient videos, during the deliberate practice stations (station 2) and should again be emphasized during this SIM.
8. Cuffed versus uncuffed endotracheal tubes – PALS and airway texts recommend cuffed endotracheal tubes in the pediatric population with the exception of newborn babies requiring intubation after delivery as per Neonatal Resucitation Program guidelines. Learners frequently brought up this learning point during the debriefs. In brief, the justification for using a cuffed tube is as follows: cuffed tubes are safer for transport with respect to tube displacement. Further, no sizing equation or application is perfect. If you are slightly off, the cuff will hopefully allow minor adjustments to manage air leaking around the tube by changing the inflation of the cuff without needing to replace the tube and risk airway trauma or other complications through repeated laryngoscopy. Previously, cuffed tubes raised concern for airway tissue necrosis and scaring but current low profile tubes and attention to this complication once the patient reaches definitve care guides the current PALS recommendations to use cuffed tubes.

***Debriefs with Sample Prompts:***

1. Thoughts / Feeling / Emotions
   1. How was this similar or different from your adult intubations?
2. If you were designing this SIM, what do you think the goals/objectives of this learning exercise were?
   1. Recognize and manage pediatric respiratory failure – basic airway management, positioning, RSI meds, mechanics of laryngoscopy
   2. Appreciate the utility of using a video laryngoscope as both a precepting tool and a confirmatory tool for proper endotracheal tube placement.
3. Specific Medical or Team based observations
   1. Keep a running list as the learners progress through the SIM
   2. FYI, sample Intubation Rubrics are below which can help SIM instructors give specific airway related feedback during the debrief
   3. FYI, some talking points below speaking to role of Video Laryngoscopy (VL) in our airway training initiatives
      1. The Short Story – Video Layngoscpy (VL) is not to replace the skill of Direct Laryngoscopy (DL), it is not “better.” Having it simply allows a teammate or preceptor to be more active in their active coaching of the procedure. Further, it is a great way to confirm endotracheal tube placement. If multiple people see the tube pass through the cords but the sats are dropping, we can more confidently advocate to use this endotracheal tube to address the hypoxia as opposed to pulling the tube and bagging! This is important because complications related to intubation increase with every additional laryngoscopy attempt.

*Finally, many of these topics are covered in depth at the following resources:*

1. Nagler, J, Balga, T, Goldman, MP. “Approach to Pediatric Emergency Airway Management Podcast.” Yale Emergency Medicine Apple Podcasts. Co-host and content developer. <https://podcasts.apple.com/us/podcast/yale-emergency-medicine-podcasts/id986369835?i=1000440671132>. 4/2019.
2. Nagler J, Nishisaki, A, Goldman MP, Johnston L, Scherzer D. Sawyer T, White ML, Auerbach M, Wolbrink TA. Pediatric Emergent Tracheal Intubation. Online video. OPENPediatrics. <https://www.openpediatrics.org/assets/video/pediatric-endotracheal-intubation.12/2019>
3. The Difficult Airway Course: Emergency and Walls, RM, et al. Manual of Emergency Airway Management, 2nd Ed, Lippincott Williams & Wilkins, Philadelphia 2004
4. Strayer, R. Emergency Ventilation in 11 Minutes. <https://vimeo.com/34883844> -

*#An example of a peri-intubation checklist is available on uptodate. Otherwise we advocate for use of whatever tool is used at your institution.*

[*https://www.uptodate.com/contents/emergency-endotracheal-intubation-in-children?search=pediatric%20emergency%20intubation%20%20management&source=search_result&selectedTitle=1~150&usage_type=default&display_rank=1*](https://www.uptodate.com/contents/emergency-endotracheal-intubation-in-children?search=pediatric%20emergency%20intubation%20%20management&source=search_result&selectedTitle=1~150&usage_type=default&display_rank=1)
