# Supplementary material for: Advanced Pediatric Emergency Airway Management: A Multimodality Curriculum Addressing a Rare but Critical Procedure
Source: MedEdPORTAL. 2020 Sep 4;16:10962. doi: 10.15766/mep_2374-8265.10962 (PMC7473185; doi:10.15766/mep_2374-8265.10962)
Supplement: Supplementary file 1 — Course Syllabus.docxStation 1 Didactic Videos.pptxStation 2 Needle Cricothyrotomy Cognitive Aid.pptxIntubation Teaching Feedback Rubrics.docxStation 3 Simulation.docxStation 4 Simulation.docxCurriculum Evaluation.docx [file mep_2374-8265.10962-s001.zip › F. Station 4 Simulation.docx]

| **Appendix F: MedEdPORTAL Simulation Case Template**  **SIMULATION CASE TITLE: Managing difficult pediatric airway in respiratory failure**  **AUTHORS:** Michael P. Goldman, MD^1^, Ambika Bhatnagar, MBBS^2^, Joshua Nagler, MD, MHPEd^3^, Marc Auerbach, MD, MSc^4^ | |
| --- | --- |
| **PATIENT NAME: Benny**  **PATIENT AGE: 7 months**  **CHIEF COMPLAINT: Unconsciousness** | |
|  | |
| **Brief narrative description of case** | This case describes EMS bringing in a 7-month-old baby with Down Syndrome who was found unresponsive in his crib. The father gave rescue breaths, started CPR and called 911. When EMS came in, they continued CPR and gave one dose of IV epinephrine. They achieved return of spontaneous circulation (ROSC) with spontaneous respiratory efforts. They report an oxygen saturation of 92% on a Non-rebreather facemask. Parents share that his neck is “unstable” and six weeks ago he was intubated for “RSV.” The learners’ goal is to anticipate a difficult airway, develop a backup plan early in the case, apply a difficult airway algorithm when intubation fails forcing the learner to rely on effective bag mask valve ventilation, airway adjuncts, consideration of a supraglottic airway and activation of local and regional pediatric critical care resources.  ***Of note, for this case, the role of team leader and airway manager is assigned by the simulation facilitator. This is because the airway manager will serve as a “plant.” We suggest asking the most senior learner in the room to assume this role. No matter how many times she tries, the airway manager knows they will not be able to pass the endotracheal tube (ETT).*** |
| **Primary Learning Objectives** | - List clinical signs, symptoms and historical factors that should raise concern for a difficult pediatric airway - Activate back-up plans under the control of the immediate provider as well as within a hospital or regional health care system - Apply a difficult airway algorithm that includes bag mask ventilation, use of airway adjunctive equipment and early consideration of a supraglottic airway device - Improve team-work and communication skills:   - Effectively pre-brief at the start of the scenario, assign roles, strategize, recap during the scenario and debrief after the scenario.   - Communicate with team members using closed loop communication, call-outs and check backs |
| **Critical Actions** | - Demonstrate initial assessment of the child including prompt primary and secondary surveys followed by a focused history. - Interpret historical and examination clues to anticipate difficult airway. - Use appropriate cardiorespiratory, oxygen saturation, end tidal CO2, and temperature monitoring. - Consider stabilizing the cervical spine during intubation. - Request Bag mask valve, Airway adjuncts (oropharyngeal and nasopharyngeal airways), a supraglottic airway, Suction, Oxygen and IV access and a shoulder rolls to optimize airway positioning. - Articulate indications for intubation. - Identify a modality for intubation. - Choose appropriate RSI medication and dosing by discussing among team members and/or by using cognitive aids. - Verbalize the need for additional personnel to be activated early in the case: for example, this case may warrant the presence of ENT, Anesthesia, NICU, and or pediatric critical care transport teams. - Articulate a back-up plan should initial attempt at intubation fail. - Select appropriate blade size, cuffed endotracheal tube size and depth - When airway manager cannot place the endotracheal tube, the team leader organizes trials of strategies to improve oxygenation and ventilation, eventually leading to the placement of a supraglottic device such as an LMA. |
| **Learner Preparation** | EMS is bringing in a 7-month-old male patient who was found unresponsive in his crib. EMS is 2 minutes away and report that the father gave rescue breaths and started CPR. EMS continued CPR on arrival and gave one dose of IV epinephrine. They quickly noted return of spontaneous circulation (ROSC) and a shallow but spontaneous respiratory effort. The patient is currently in sinus rhythm with an oxygen saturation of 92% on a Non-rebreather facemask (NRB). EMS also mention that the parents told them he has Down Syndrome and that the family was told that if he ever gets very sick, they need to communicate that his neck is “unstable.”  Learners will get 60 seconds to prepare, assign roles, and then it will be announced, “The patient has arrived”. |

| Initial Presentation | | | |
| --- | --- | --- | --- |
| **Initial vital signs** | HR-180 (sinus rhythm)  BP-100/50  RR-15; shallow and irregular  SpO2-92% on Non-Rebreather oxygen mask  Temp-38.5°C | | |
| **Overall Appearance** | Altered level of consciousness. | | |
| **Actors and roles in the room at case start** | The patient’s mother is present in the room and EMS has now left. The mother played by additional faculty or the SIM facilitator will give the history.  *Team member confederate (we recommend using the most experienced Physician on the team): This team member knows the kid from previous admissions and mentions difficult airway experience in the past with this patient. This physician takes lead (airway operator) on intubating the kid and fails to insert the endotracheal tube on multiple attempts. | | |
| **HPI** | Mother will tell the learners voluntarily that:   - “Benny has Down’s Syndrome and we were told that if he is ever very sick, we need to communicate that his neck is unstable.”   Mother gives the following information when asked:   - “I first noted him not moving and being face down approximately 10 minutes after being placed in crib. He looked so terrible we called 911, his father blew into his mouth and nose and started CPR. Thankfully EMS was there in under 5 minutes and took over!” - “He has been well this past week” - “Feeding normally via G-Tube” - Development- delayed - Vaccines - up to date for age | | |
| **Past Medical/Surgical History** | **Medications** | **Allergies** | **Family History** |
| Ex-36 weeker  Prenatally diagnosed with Down Syndrome. He had a recent PICU stay for respiratory failure from RSV.  He also has reflux and constipation | Omeprazole, Miralax, and all feeds go through his G-Tube | None | None |
| **Physical Examination** | | | |
| **General** | Stertor and upper airway congestion noises. | | |
| **HEENT** | Pupils 6 mm and minimally reactive bilaterally | | |
| **Neck** | Normal | | |
| **Lungs** | Abnormal rate and depth, coarse breath sound bilaterally. | | |
| **Cardiovascular** | Strong pulses at 2+, tachycardia. | | |
| **Abdomen** | Normal | | |
| **Neurological** | Altered | | |
| **Skin** | No bruising or trauma findings. | | |
| **GU** | Normal | | |
| **Psychiatric** | NA | | |

| Instructor Notes - Changes and CASE Branch Points | | |
| --- | --- | --- |
| **Intervention / Time point** | **Change in Case** | **Additional Information** |
| 0 minutes after EMS pre-brief |  | Confederate: “Oh my goodness, I know this family… 2 months ago he was intubated by anesthesia after several attempts for RSV and it was a disaster!” |
| Bag valve mask | Oxygen saturation 96% |  |
| Any of the following maneuvers used:  Jaw thrust only (no head tilt/chin lift out of concern for his “unstable neck.”  Two-person bagging technique to maximize seal  Use of nasopharyngeal airway Venting of G-Tube. | Oxygen saturation >96% | All of these maneuvers will assist his basic airway support.  Learners may be aware that this patient is a high risk an intubation. As such, they may elect to stop further intervention and try to activate a transfer or solicit additional personnel / resources. This is actually a very astute management decision.  If this occurs, one can mimic the following scenarios based on the learning group’s work environment:  -The PICU is over census and cannot accept the patient immediately  -The Pediatric Critical Care Transport Team is an hour away.  This will force the team to continue to actively manage the patient and progress through a difficult airway algorithm. |
| Oropharyngeal airway before paralysis | Gag reflex intact. Patient vomits.  Oxygen saturation decreases to 75% |  |
| Participant requests finger stick blood glucose. | Glucose level is 100. |  |
| 6-10 minutes | Now also frothing at mouth.  HR-90s  BP-100/50  RR-decreasing trend  Trend oxygen saturation from 94% to 75% over 1 minute. | Mother is concerned because someone told her the baby’s neck is not stable.  She also says “Last time, placing the breathing tube was a total mess!” |
| Attempt to intubate | Quickly drop oxygen saturation from 85% to 75%. If atropine was not given, drop HR to 60s | Confederate (airway operator) will not be successful in inserting the endotracheal tube no matter what.  Mom starts crying out… “not this again!” |
| If intubation plan is aborted in favor of bagging AND team uses 2 person bagging technique or appropriately uses an oropharyngeal airway (OPA) or nasopharyngeal airway (NPA). | Bring oxygen saturations up to 94% | Patients who are comatose or chemically paralyzed are eligible for an OPA.  NPAs should not be used in the facial / nasal trauma patient. |
| If intubation is attempted without paralysis. | Make the patient vomit and drop oxygen saturation to 75% | Atropine – 0.02 mg/kg IV/IO  Etomidate – 0.3mg/kg IV/IO  Ketamine – 1.5-2 mg/kg IV/IO |
| After RSI administration | Patient stops breathing  Post RSI vitals:  HR 100 (70 if no atropine)  BP 100/50 (85/45 if propofol used for induction)  RR 0 | Rocuronium – 1-1.2mg/kg IV/IO  Succinylcholine – 1 - 1.5mg/kg IV/IO |
| If bagging is going well – using techniques as above | SpO2: 94% | The transport team (on phone) or confederate team member will suggest placing an LMA. |
| If bagging is going poorly | SpO2: 82% | The confederate team member or sim facilitator can give hints on how to improve the bagging, then, transport team will ask for LMA to be placed. |
| If team proceeds to cricothyroidotomy before the LMA. |  | The confederate team member or sim facilitator guide team towards the bagging and LMA interventions |
| Airway secured with LMA | HR-120  BP-100/50  SpO2-97%  RR-0 | Sign out to transport team. |

**Ideal Scenario Flow**

The learners enter the room to find an infant with noisy breathing and oxygen saturations at 92% on a non-rebreather. They immediately put the patient on bedside monitors and ask the nurse to get additional IV access. While taking a focused history from the mother and examining the patient, learners recognize and announce to the group that the child has an anticipated “difficult airway.” This is further emphasized by the confederate announcing she “knows this patient from the last time.” The team stabilizes the spine, suctions the airway and uses bag valve mask with jaw thrust and a two-handed BVM technique to provide effective ventilation. However, after 5 minutes excessive frothing is seen in patient’s mouth and oxygen saturation starts dropping. Anticipating a difficult airway, the team leader recognizes the need to intubate which is why the most experienced team member is assigned to airway management (as opposed to team leader). Based on local resources and protocols, the team activates additional personnel such as ENT, anesthesia and/or a pediatric critical care transfer teams. The team leader verbalizes the plan to intubate with appropriately sized intubation and backup equipment (endotracheal tube, laryngoscope blade, OPA, NPA and LMA). RSI medications should include atropine in this age as a premedication along with both a sedative and a paralytic. After RSI medications are administered, the airway operator attempts to intubate but fails to insert the endotracheal tube. Ideally, the team leader then immediately activates the pre-planned back up plan and guides bagging the patient with a two-handed technique. The team leader then decides to secure the airway with an LMA and signs out to transfer team.

*Potential Branch Points*

- While the case nearly forces the learners to intubate the patient, it is vital for learners to practice and use their basic airway maneuvers, especially when the patient is an anticipated difficult airway. For this case however, one layer of complexity on top of the patient’s acute decompensation is the history of Down Syndrome. Patients with Down Syndrome are at high risk for atlanto-axial instability in the cervical spine. As such, caution must be exercised with the amount of neck manipulation used during advanced airway management. This condition, along with other Down Syndrome features (e.g. small mouth / large tongue, possible cardiac, neurologic and other comorbidities) make this patient very high risk. This is further emphasized by historical data such as the confederate sharing how challenging it was the last time the patient required intubation.
- A finger stick blood glucose check is a key step in the management of any sick baby or toddler as they have such minimal reserve. In this case, it is normal to focus the learner back to the airway management.
- During Intubation, regardless of how experienced the airway manager is, the patient is essentially unable to be intubated. We anticipate teams to try again but ideally not a third or fourth time. Should a team continue to try for intubation, the SIM facilitator can encourage the use of bagging or suggest a call to the pediatric critical care resource who can push the team towards a difficult airway management algorithm that prompts excellent basic airway management skills and or an LMA.
- During intubation, the use of a paralytic may or may not be used. If the team states they are purposefully not using one out of concern for difficult airway, this is valid. If no mention of this decision is made, the child vomits. Essentially, this serves to bookmark this decision for the debrief discussion.

**Anticipated Management Mistakes**

1. No back up plan – Given the layers of data that push the learner to consider the idea of a difficult airway scenario (e.g. Down Syndrome, mom’s concerns, the airway manager recalling how challenging intubation was the last time, etc…), we have yet to run this SIM where some form of a backup plan is not articulated. Even when a minimally detailed plan is mentioned, the fact that the patient “cannot be intubated” pushed all the teams to consider additional equipment, personnel and resources to best manage this patient during the debrief. Further, the SIM “ends” one way or another with the placement of an LMA.
2. Advanced Airway Management starts with Basic Airway Management – While our curriculum focuses a lot on invasive airway management skills, we quickly realized the importance of emphasizing the critical importance of basic airway management skills at all four learning stations. Specifically, we advocate for the application of a shoulder roll to assist with airway patency and excellent bag mask ventilation, especially having two hands on the mask and an additional provider squeezing the bag. When learners did not pay attention to this skills in the SIM, we found that worsening the hypoxia moved them to address this with their basic airway management skills.
3. Failure to use RSI meds/ paralytics – There are very few indications for placing an endotracheal tube or LMA without RSI. We emphasized in all of our learning stations that an intubation is always a very organized, thoughtful and yes, urgent procedure. RSI meds and paralytics help to improve first pass success rates and in this age group, the use of Atropine is advocated for by PALS. Further, the decision around paralytics for this patient is a great topic to review during the debrief and has come up several times. Given how risky this patient is, sedation without paralytics up until the point of laryngoscopy or LMA insertion are appropriate.
4. Failure to account for cervical spine instability – While there are no definitive recommendations as to how to manage the *possibility* of atlantoaxial instability, simply raising how this condition further contributes to the fact that the patient should be considered a difficult airway. Some groups have placed a c-spine collar, others hold in-line cervical spine stabilization during intubation attempts. Regardless, this also has come up several times during our debriefs.
5. Early needle cricothyrotomy prior to supraglottic device – A few teams have prepared equipment for needle cricothyrotomy and one in particular spent several minutes gathering the supplies while paying little attention to the rapidly decompensating patient. For this case, we simply advanced the patient’s condition quicker forcing intervention with basic airway maneuvers. We also markedly improved the patient’s condition once these basic maneuvers were implemented. It would be acceptable for a team leader to designate a teammate to gather such supplies and close the loop back with the team leader once all supplies are ready. We have also successfully used the confederate and or the pediatric critical care transfer team to guide the team leader towards LMA placement, certainly before needle cricothyrotomy is attempted.

Debrief:

1. Thoughts / Feeling / Emotions
   1. How was this similar or different from your adult difficult intubations?
2. If you were designing this SIM, what do you think the goals/objectives of this learning exercise were?
   1. Anticipate a difficult airway from historical (i.e. Down Syndrome and associated co-morbidities, recent visit with situation awareness from team members, review of anesthesia records, etc…) and physical exam findings – (i.e. the LEMON mnemonic -Look, Evaluate, Mallampati, Obstruction, Neck Mobility).
   2. Articulate backup plans *before* initiating intubation as it is easier to assume and plan for it to go wrong as opposed to assuming it will always go right!
   3. Apply a difficult airway algorithm.
   4. Know your local resources for airway backup and activate pediatric critical care transport early if this is relevant to your work environment.
   5. Familiarize yourself and practice using airway adjuncts and supraglottic devices.
3. Specific Medical or Team based observations
   1. Keep a running list as the learners progress through the SIM
   2. FYI, sample Intubation Rubrics are below which can help SIM instructors give specific airway related feedback during the debrief
   3. FYI, some references listed speaking to
      1. Predicting difficult airways
      2. Algorithms for difficult and / or failed airways
      3. Focusing on ventilation skill set as the most important airway manager procedural skill to master and maintain.

*Finally, many of these topics are covered in depth at the following resources:*

1. Nagler, J, Balga, T, Goldman, MP. “Approach to Pediatric Emergency Airway Management Podcast.” Yale Emergency Medicine Apple Podcasts. Co-host and content developer. <https://podcasts.apple.com/us/podcast/yale-emergency-medicine-podcasts/id986369835?i=1000440671132>. 4/2019.
2. Nagler J, Nishisaki, A, Goldman MP, Johnston L, Scherzer D. Sawyer T, White ML, Auerbach M, Wolbrink TA. Pediatric Emergent Tracheal Intubation. Online video. OPENPediatrics. <https://www.openpediatrics.org/assets/video/pediatric-endotracheal-intubation.12/2019>
3. The Difficult Airway Course: Emergency and Walls, RM, et al. Manual of Emergency Airway Management, 2nd Ed, Lippincott Williams & Wilkins, Philadelphia 2004
4. Strayer, R. Emergency Ventilation in 11 Minutes. <https://vimeo.com/34883844> -
