# Supplementary material for: Advanced Pediatric Emergency Airway Management: A Multimodality Curriculum Addressing a Rare but Critical Procedure
Source: MedEdPORTAL. 2020 Sep 4;16:10962. doi: 10.15766/mep_2374-8265.10962 (PMC7473185; doi:10.15766/mep_2374-8265.10962)
Supplement: Supplementary file 1 — Course Syllabus.docxStation 1 Didactic Videos.pptxStation 2 Needle Cricothyrotomy Cognitive Aid.pptxIntubation Teaching Feedback Rubrics.docxStation 3 Simulation.docxStation 4 Simulation.docxCurriculum Evaluation.docx [file mep_2374-8265.10962-s001.zip › G. Curriculum Evaluation.docx]

**PEDIATRIC AIRWAY COURSE**

**Date:** ____

**Position (choose one):** *MD___ RN ____ Tech ____ Resp Therapist ____ PA/NP _____ Other:_____*

*Years in Practice:_____*

*Years at your current position at (______): ___*

| **Please rate your agreement with the following statements:** | | | | | | |
| --- | --- | --- | --- | --- | --- | --- |
|  | **Strongly Disagree** | **Disagree** | **Neutral** | **Agree** | **Strongly Agree** | **Not**  **Applicable** |
| I was comfortable with pediatric airway management BEFORE today | 1 | 2 | 3 | 4 | 5 | n/a |
| I am MORE comfortable with pediatric airway management AFTER today | 1 | 2 | 3 | 4 | 5 | n/a |
|  | **Strongly Disagree** | **Disagree** | **Neutral** | **Agree** | **Strongly Agree** | **Not**  **Applicable** |
| The Airway Lecture and Video Session added to my knowledge/skills/attitudes of pediatric airway management | 1 | 2 | 3 | 4 | 5 | n/a |
| The Mini Skills Station for Pediatric Airway Management (Speed/Accuracy Drills game and Needle Crics) added to my knowledge/skills/attitudes of pediatric Airway Management | 1 | 2 | 3 | 4 | 5 | n/a |
| The Respiratory Failure Station Emphasizing VL for Airway Precepting and ETT Placement added to my knowledge/skills/attitudes of pediatric airway management | 1 | 2 | 3 | 4 | 5 | n/a |
| The Respiratory Failure / Difficult Airway Station added to my knowledge/skills/attitudes of pediatric airway management | 1 | 2 | 3 | 4 | 5 | n/a |

**List one “take away” that you will apply in the future when taking care of a pediatric patients:**

Learning Airway through videos didactic 1. ______________________________

Airway speed and accuracy drills 1. _______________________________

Needle Crics – When, Why and How 1. _______________________________

Benefits of VL for Airway Management SIM 1. ______________________________

Can’t Intubate Predictors and Backup Plans SIM 1. _______________________________

**List the top 3 clinical topics you feel most uncomfortable with related to caring for infants/children**

1. _______________________________

2. _______________________________

3. _______________________________

**List the total number of critically ill pediatric patients you have cared for in each location:**

PED _____ General ED _____ Simulation ______ Other_______

***A FEW MORE QUESTIONS ON THE REVERSE SIDE, PLEASE!!!!!***

**Where there any stations today that you felt were particularly helpful for your learning?**

**Where there any stations you felt were redundant / had minimal added educational value for your learning?**

**Please share any additional feedback regarding today’s session:**

**Please provide any additional feedback regarding additional pediatric experiences / education / training you desire in a future educational initiative:**
