# Supplementary material for: Advanced Pediatric Emergency Airway Management: A Multimodality Curriculum Addressing a Rare but Critical Procedure
Source: MedEdPORTAL. 2020 Sep 4;16:10962. doi: 10.15766/mep_2374-8265.10962 (PMC7473185; doi:10.15766/mep_2374-8265.10962)
Supplement: Supplementary file 1 — Course Syllabus.docxStation 1 Didactic Videos.pptxStation 2 Needle Cricothyrotomy Cognitive Aid.pptxIntubation Teaching Feedback Rubrics.docxStation 3 Simulation.docxStation 4 Simulation.docxCurriculum Evaluation.docx [file mep_2374-8265.10962-s001.zip › _Educational_Summary_Report_10962.pdf]

MedEdPORTAL® The AAMC Journal of Teaching and Learning Resources

Original Publication Open Access

# Advanced Pediatric Emergency Airway Management: A Multimodality Curriculum Addressing a Rare but Critical Procedure

Michael P. Goldman, MD*, Ambika Bhatnagar, MBBS, Joshua Nagler, MD, MHPEd, Marc A. Auerbach, MD, MSc

*Corresponding author: michael.goldman@yale.edu

## Abstract

**Introduction:** Advanced airway management in pediatrics is a rare, high stakes skillset. Developing proficiency in these skills is paramount, albeit challenging. Providers require innovative approaches to address initial training and maintenance of procedural competency. To address this, we developed a multimodality curriculum. **Methods:** Through an interactive problem-based learning session utilizing real intubation videos, hands-on skill stations, and two simulation-based scenarios, participants advanced through educational objectives towards the goal of improving perceived comfort, knowledge, skills, and attitudes in emergency pediatric advanced airway management. Content was developed by integrating varied learning modalities under the *learn, see, practice, prove, do, maintain* construct. Please note the specialized equipment needed for this curriculum included pediatric airway trainers and a video laryngoscope. **Results:** We have conducted the curriculum in its entirety four times, reaching 131 interdisciplinary participants. Forty-nine physicians of varying training backgrounds and clinical working environments completed postparticipation evaluations. On a Likert scale ranging from 1 (*strongly disagree*) to 5 (*strongly agree*), a significant improvement in perception of comfort with managing the emergent pediatric airway was noted (2.7 to 4.6, $p < .0001$). Further, 94% of participants reported they strongly agreed (71%) or agreed (23%) that each station added to their perceived knowledge, skills, and attitudes of pediatric airway management. **Discussion:** After participating in our curriculum, participants self-reported improved comfort in managing the emergent pediatric airway. This curriculum provides educators with resources to navigate the paradigm of obtaining and maintaining competency of a rare but critical skillset.

**Keywords**
Airway Management, Pediatric Emergency Medicine, Intubation, Respiratory Failure

## Educational Objectives

By the end of this curriculum, learners will be able to:

1. Review and apply key pediatric anatomic and physiologic features that promote successful assessment and management of the pediatric airway.
2. Deliberately practice and receive coaching on varied laryngoscopy and failed airway skill sets.
3. Recognize and manage respiratory failure in a pediatric patient.
4. Predict, plan for, and manage the difficult pediatric airway.

**Citation:**
Goldman MP, Bhatnagar A, Nagler J, Auerbach MA. Advanced pediatric emergency airway management: a multimodality curriculum addressing a rare but critical procedure. *MedEdPORTAL*. 2020;16:10962. https://doi.org/10.15766/mep_2374-8265.10962

## Introduction

The initial training and subsequent maintenance of competency for pediatric advanced airway management is a prime example of the medical educators' challenging task of adequately addressing a rare and difficult skill set.[1-3] Contributing to this challenge are the facts that actual pediatric critical care is uncommon (when compared to the adult population), the distribution of where children seek emergency medical care and who is there to care for them varies, and there are many competing demands on a health care providers' initial and continuing medical education.[4-11] Further compounding these facts is the variation in the rate and quality of procedural skill acquisition amongst each individual learner. This supports the need to utilize varied educational modalities and providing customizable frequencies of practice to meets all learners' needs.[3,12-18]

In the face of this challenge, we developed our curriculum through the construct of *learn, see, practice, prove, do, maintain,* progressing learners through variable learning modalities

Copyright © 2020 Goldman et al. This is an open-access publication distributed under the terms of the Creative Commons Attribution license. 

and exposing them to novel airway educational interventions such as the use of real patient laryngoscopy videos.[14,15,19-22] As such, our curriculum differs from existing resources and thereby offers a unique contribution to the literature as it offered extensive resources specific to pediatrics, highlighted the video laryngoscope as both a patient safety and educational adjunct, and provided learners multiple opportunities to apply their understanding of key concepts through both deliberate practice, coaching, and simulation.[12,19,23-25]

While components of our curriculum can be implemented with any learner along the medical education continuum, we have targeted our curriculum to the following learners: senior pediatric residents, pediatric emergency medicine fellows, pediatric intensive care fellows, emergency medicine residents, critical care transport paramedics and community hospital emergency medicine physicians, physician assistants, and nurse practitioners. We felt this cadre of learners were primed with basic airway management principles and best prepared to engage in more advanced discussions, practice, and high-fidelity simulations that would push their medical knowledge, skills, and comfort to a higher level than before participation in our curriculum.[26,27] We highlighted the latter groups of community hospital-based learners based on epidemiologic trends of where pediatric critical illness is often initially managed, and based on our author group's and others' prior experiences using in-situ simulation as a tool to gain a deeper understanding of the challenges providers face with respect to their pediatric critical care skills.[4-8,11,25,28-30]

Our goal in sharing this curriculum was for future facilitators and their learners to appreciate how the use of multiple learning modalities helps reach a wide array of learners and can fill a training gap relevant to the care of acutely ill and injured children.

## Methods

Development

We developed a curriculum grounded in procedural educational theory that embraced the challenges of the initial training and subsequent maintenance of the competency of emergent pediatric airway management.[14] As part of the iterative development of the curriculum, we piloted individual components with pediatric emergency medicine fellows, community hospital providers, and pediatric senior residents, prioritizing the fine-tuning of the curriculum's components and the development of a robust facilitator's syllabus (Appendix A). Additionally, our curriculum was reviewed by experts in pediatric emergency airway management inclusive of faculty practicing pediatric emergency medicine, pediatric anesthesiology, and pediatric otolaryngology.

Equipment/Environment

*Station 1:* Reviewing key pediatric airway management considerations

- PowerPoint (Appendix B)
- AV connection
- Laser pointer

*Station 2:* Choosing the right equipment quickly and needle cricothyroidotomy

- Intubation trainers (e.g., Syndaver Child and Infant)
- Laryngoscope handles
- Laryngoscope blades–Wis-Hipple 00, Miller 0, 1, 2; Macintosh 1, 2, 3
  - Video laryngoscope blades and handles
- Cuffed endotracheal tubes sizes 3.0, 3.5, 4.0, 4.5, 5.0, 5.5, 6.0, 6.5
- Laryngeal mask airway (LMA) sizes 1, 1.5, 2, 2.5, 3, 4, 5
- Cognitive aids (e.g., Broselow tape; Patient Advice and Liaison Service, PALS, cards; PediStat app; e-Broselow app)
- Stopwatch
- Needle cricothyrotomy trainer
- Needle cricothyrotomy cognitive aid (Appendix C)
  - 14g IV
  - 3mL syringes
  - Top of a 7.0 endotracheal adapter
  - Pediatric self-inflatable bag valve mask
- Intubation teaching rubrics (Appendix D)[29,31]

*Station 3:* Simulation 1–impending respiratory failure in the pediatric patient

- Simulation scenario instructor guide (Appendix E)
- SIM Baby with IV in place, no additional moulage
- Blades, Wis-Hipple 00, Miller 0, 1, 2, Macintosh 2, 3
- Cuffed endotracheal tubes of variable sizes
- Video laryngoscope (e.g., Glidescope or CMAC system)
- Self-inflating bag and masks sizes infant, pediatric/child, and adult
- Cognitive aids (e.g., Broselow tape, PALS cards, PediStat app, e-Broselow app)
- Airway adjuncts–nasopharyngeal airways, oropharyngeal airways of varying sizes
- Airway rescue devices–supraglottic airway such as an LMA
- Code and rapid sequence intubation (RSI) medication tray
- Intubation teaching rubrics (Appendix D)[29,31]

Copyright © 2020 Goldman et al. This is an open-access publication distributed under the terms of the Creative Commons Attribution license. 

*Station 4:* Simulation 2–the difficult pediatric airway

- Simulation scenario instructor guide (Appendix F)
- SIM Baby with IV in place, no moulage
- Blades, Wis-Hipple 00, Miller 1, 2, Mac 2, 3
- Cuffed endotracheal tubes of variable sizes
- Video laryngoscope such as Glidescope or CMAC system
- Self-inflating bag and masks sizes infant, pediatric/child, and adult
- Cognitive aids (e.g., Broselow tape, PALS cards, PediStat app, e-Broselow app)
- Airway adjuncts–nasopharyngeal airways, oropharyngeal airways
- Airway rescue devices–supraglottic airway
- Code and RSI medication tray
- Intubation teaching rubrics (Appendix D)[29,31]

Personnel

We have run the full curriculum four times in the last 2.5 years. Each time, the number of instructors varied based on the size of the learning group. While the curriculum is ideally delivered in sequential order, we have successfully run the curriculum with all four stations occurring simultaneously with participants rotating from station to station. As such, the minimum number of instructors for a small group of learners was three: one delivered the content, one assisted the main facilitator with direct procedural feedback and debriefing the simulations, and one operated the simulation technology. For the times we ran the curriculum in four separate rooms simultaneously, a minimum of two facilitators per room was required. Should the future facilitator have additional faculty at their disposal, we placed additional staffing in station 2. This allowed for learners to receive the maximal individual deliberate practice and coaching of the many kinesthetic skills covered during this station. Finally, optional additional personnel included actors who can serve as distraught family members during the simulations. All faculty teaching the curriculum should have adequate training and experience in pediatric emergency airway management. All preparation materials for faculty are included in the syllabus to promote standardization of teaching content and minimize teaching preparation time for busy instructors (Appendix A).

Our smallest number of participants was a group of five pediatric hospitalists. Our maximum number of participants included a diverse learning group of 50 participants from a neighboring community hospital site. There is no minimum number of participants needed, though ideally a team of at least three participants is best to facilitate realistic care teams during the simulations. The maximum number of participants to enroll is capped by the number of roles one can assign during the simulations and should best mimic the learners' working environment to promote realism. When we were presented with higher learner numbers, we assigned roles to participants to promote active observation and reflection versus passive observation.[32-34]

Implementation

To reach our target learners for this curriculum, we dedicated a standing pediatric procedure-oriented teaching day with our institution's emergency medicine residency program. Similarly, we used a dedicated in-situ simulation teaching day to deliver this curriculum to a long-standing community hospital partner. Subsequently we were approached by a pediatric hospitalist division and were asked by the emergency medicine residency leadership to run the curriculum again as two new classes of learners had joined their program since our initial contact with this group. Further, individual components of the curriculum were used multiple times in the ongoing pediatric airway management education of our pediatric emergency medicine fellows and senior pediatric residents.

The logistics, equipment needs, overarching learning goals, and the specific objectives for each learning station of our curriculum were outlined in depth in the course syllabus (Appendix A). The full curriculum required on average 3.5 hours to complete. However, this time has varied based on the numbers of learners participating, and whether or not we have included a lunch break. One could consider splitting the learning into two or more time frames within the same week or month depending on teaching time allotments at a given institution. We have run our course when a small number of facilitators progressed through the stations in sequential order together with a small learning group. However, most of the time we delivered our curriculum to large learning groups which required stations to run simultaneously. As such, a sample rotation schedule is offered in the syllabus (Appendix A).

In all four implementations, participants started together in a conference room for a brief overview of the goals of the curriculum, an introduction to each of the four learning stations through a review of each station's individual learning objectives, and orientation to the facility and rotation plan.

For the initial station, the main curriculum facilitator used a PowerPoint with graphics, slide annotations, and real patient intubation videos (Appendix B). Each video offered the opportunity to discuss varying anatomy and laryngoscopy techniques and tips.[19]

Copyright © 2020 Goldman et al. This is an open-access publication distributed under the terms of the Creative Commons Attribution license.

At station 2, intubation mannequins and a needle cricothyrotomy model were used. At the airway trainer mannequins, multiple sizes of endotracheal tubes and blades were purposefully placed together in a disorganized pile in front of the participant. Preceptors timed how long it took to prepare first without, and then with, a cognitive aid such as a PALS card, Broselow tape, or a smartphone application like PediStat. This demonstrated how the use of a cognitive aid improved a learner's procedural chronometry.[35] Please note, this curriculum did not endorse any particular cognitive aid, rather we advocated for the consistent use of a learner's preferred aid so that it is readily accessed and used during real, high stakes cases. Thereafter, facilitators provided direct observational feedback and coaching as learners practiced intubation and laryngeal mask airway (LMA) placement on different-sized airway trainers. Examples of previously published and validated intubation teaching rubrics were provided to facilitators to promote standardization of teaching scripts and to ensure key procedural techniques were covered (Appendix D).[29,31] In this station, an additional preceptor worked with learners on needle cricothyrotomy. There learners reviewed indications, demonstrated the proper technique, and received direct observation and feedback on their approach. A needle cricothyrotomy cognitive aid was developed and provided to learners to take home (Appendix C). Of note, on one occasion we had limited faculty and ran this activity as independent learning where participants watched a brief instructional video and practiced the technique with a peer; this optional video resource is included in Appendix A.

The simulations at stations 3 and 4 provided an opportunity to apply and expand upon the learners' expanding emergency pediatric airway management knowledge and skills, while also serving to assess latent safety threats pertaining to emergency airway management within the participants' institution when the simulation was run in-situ (Appendices E and F).[36] Learners engaged with the simulation exercise for 10-15 minutes and then debriefed the scenario for approximately 20 minutes. Debriefing prompts were provided in the simulation appendices. The postparticipation curriculum evaluations completed the learning experience (Appendix G).[37,38]

### Debriefing

Each of the four learning stations offered unique opportunities for debrief, discussion, direct observation, and feedback. In the initial interactive didactic station, the videos prompted the learning group to engage in active discussion to clarify and solidify the learning points. In our experiences, this was facilitated by keeping the size of the learning group below 15. For the intubation and needle cricothyrotomy station, learners engaged with peers and faculty through deliberate practice and coaching on their preparation and their laryngoscopy techniques.

The model implemented for debriefing the simulations favored a facilitator-guided method which focused on both medical knowledge gaps and systems threats brought to the forefront during the simulation.[39-41] Facilitators were provided additional debriefing prompts at the end of each simulation scenario (Appendices E and F).

### Assessment

For our first three implementations, we used an immediate, paper, postparticipation evaluation encouraging a retrospective pre-/postreflection.[37,38] Subsequently, we used a mobile phone-friendly electronic survey tool to promote evaluation completion rate (Qualtrics, Provo, UT).

After obtaining basic demographics on our learners, they were asked to reflect upon their perceived change in comfort, knowledge, and skill set with respect to pediatric airway management. Additionally, they were asked how each station contributed to their learning (Appendix G). Statistical analysis for our main question was performed using paired *t* tests.

Respondents also had an opportunity to provide insight through free text response addressing what type of emergency pediatric cases make them most uncomfortable and how they would suggest improving our curriculum.

## Results

As of January 15, 2020, we have run our curriculum in its entirety four times, reaching a large number of learners with varied backgrounds, training, practice settings, and pediatric experiences. Full curricula were given to an emergency medicine residency group twice, spaced 2 years apart, a community-based hospital's general emergency medicine team, and a community-based hospital's pediatric hospitalist division, totaling 131 learners. Included in these 131 participants were emergency medicine trainees, emergence medicine attending physicians, pediatric hospitalist attending physicians, advanced practice providers, nurses, respiratory technicians, medical students, and physician assistant students.

We collected and analyzed 60 postparticipation evaluations that were completed by our curriculum's target learners. Of these 60 evaluations, the majority of respondents were physicians (49), most of whom were residents in training (37; Table). Twelve participants were attending physicians, seven were emergency

Copyright © 2020 Goldman et al. This is an open-access publication distributed under the terms of the Creative Commons Attribution license. 

medicine trained, and five were pediatric hospitalist trained. The remainder of respondents were nurses (6), one technician, one respiratory therapist, two physician assistant students, and one did not provide their professional role.

The two emergency medicine sessions engaged 76 trainees, 42 at the first and 34 at the second. Data were returned from a total of 37 emergency medicine trainees, 13 after the first session and 24 after the second. In total, 16 interns, nine second-years, seven third-years, four fourth-years, and one other who did not provide their postgraduate year of training (Table).

Analysis of our sample of immediate postparticipation questionnaires resulted in the following quantifiable feedback of our curriculum. When learners were asked to rank their agreement with the statement, "I was comfortable with emergency pediatric airway management before (and after) the curriculum" on a 5-point Likert scale ranging from 1 (*strongly disagree*) to 5 (*strongly agree*), a significant improvement in perception of comfort (2.7 to 4.6, $p < .0001$) was noted. Further, 94% of participants reported they strongly agreed (71%) or agreed (23%) that all four stations added to their knowledge, skills, and attitudes of emergency pediatric airway management.

Qualitative analysis of responses recorded on our postparticipation questionnaire were assessed for common themes. First, the desire for additional training on the management of the emergent pediatric airway was reported as a major theme for all learning groups. This was supported by the most common responses to the probe, "What about emergency pediatric care makes you the most uncomfortable?" were pediatric "respiratory failure," "intubation," and "the difficult pediatric airway." Second, the use of the video laryngoscope for team-based intubations and subsequent learning from real intubation videos were identified as highly regarded learning modalities. Finally, learners appreciated the emphasis on basic

**Table.** Characteristics of the 49 Physician Participants who Completed Postcurriculum Evaluations

| Level of Training (*n*) | Training Background (*n*) | Current Practice Environment (*n*) |
|---|---|---|
| Attending (12) | Emergency medicine (7)<br>Pediatrics (5) | Community hospital emergency room (7)<br>Community hospital pediatric inpatient ward (1)<br>Academic children's hospital inpatient ward (4) |
| Trainee (37)[a] | Emergency medicine (37) | Academic general emergency department and academic children's hospital emergency department (37) |

[a]Trainee level: PGY 1 ($n = 16$); PGY 2 ($n = 9$); PGY 3 ($n = 7$); PGY 4 ($n = 4$); PGY 5 ($n = 1$).

airway management skills (as opposed to just laryngoscopy skills) as important tactics to practice and master in order to stabilize patients while gathering additional personnel and equipment during challenging clinical scenarios.

## Discussion

Medical educators, especially those charged with teaching critical care procedures, face multiple impediments to bringing their learners to competency and mastery, and then face a similarly daunting feat to maintain these skill sets, especially when these skills are utilized infrequently in the clinical setting. As such, creative modalities that aim to reach a specific set of learners[26] who may have variable learning characteristics[13,17,42,43] are needed to build learners' confidence, knowledge, and skills in these infrequent but critical procedures.

Consistent with Meinema et al's framework for describing educational interventions and grounded in Sawyer et al's framework for learning procedures, we shared our experience developing, implementing, and reflecting upon our pediatric emergency airway management curriculum, which aimed to guide the learner from the lecture hall to the simulation lab and then to the bedside with greater confidence and preparedness to address this challenging medical scenario.[14,37]

The curriculum had a number of unique strengths. First, we acknowledged the stress and challenge of this clinical scenario and recognized up front that each learner will learn each skill differently and at differing paces. Thus, providing opportunities for repetitive exposures and multimodality learning technologies were key to our work.[13-15,21,22,25,29,32,33] Second, we synergized relevant educational and pediatric emergency airway management literature. This is highlighted by our emphasis on learning the intubation technique through a review of intubation videos that captured native pediatric airway anatomy and promoted team-based video intubation during the simulations.[19,44-46] Third, the novel concept of measuring proficiency through chronometry applied greatly to this procedure that has a variety of infrequently utilized equipment that needs quick organization within a high stress clinical setting.[35] Fourth, we emphasized the use of cognitive aids to assist in cognitive unloading during these high stakes procedures, facilitating a comfort likely to translate to real clinical scenarios.[44]

Finally, an additional strength of our curriculum included the ability to tailor the instruction to the available allotment of learning time or practice setting at a given institution. While we recommended the four stations in succession, we believe each

Copyright © 2020 Goldman et al. This is an open-access publication distributed under the terms of the Creative Commons Attribution license.

component can be used on its own. This allows instructors to consider their learners' particular needs and aim to meet them at the most appropriate educational junction. In a similar vein, we did not mandate a single simulation debrief methodology. Anticipating that each simulation may elucidate different teachable moments, flexibility while facilitating the debrief to fit the learning environment is encouraged.[39,47] As a foundation, we referred the future facilitator to the promoting excellence and reflective learning in simulation (PEARLS) method of debriefing which integrated general reactions after physically stepping away from the simulation (learner self-reflection), probing for facts of the case (facilitating focused discussion), focused teaching through sharing observations gleaned from the simulation, and finally a summary of key learning take-home points.[48]

We encountered several challenges over the four full-day sessions which we share with our future facilitators in hopes of promoting smooth future sessions. First, running the entire curriculum as designed required a significant investment of time, resources, and personnel. We provided our thoughts and experiences with respect to a minimum number of facilitators, but encourage the organizer to try and form a dedicated cadre of co-facilitators to ensure content is delivered in-depth and as per the course syllabus as much as possible. We have also had to add in additional teaching stations that were outside of the above components of our airway curricula simply to ensure the learning groups stayed small to promote active participation of all learners and provide the best opportunity for individual deliberate practice and coaching. Second, while we had recommended certain teaching tools, we recognize that available resources vary across learning centers. Some centers may not have a video laryngoscope, for instance, which will limit the ability to directly apply select portions of our curriculum. However, the prerecorded videos of native airway anatomy will still be useful during station 1 and may spark interest for investment in this important airway management and educational adjunct. Third, even when the equipment was available, the listed equipment was extensive and required a significant amount of time to organize each station. This may prompt future facilitators to use their medical simulation centers over in-situ simulation to allow for set-up to potentially occur the evening before, and to work with simulation center staff who can become familiar with the detailed needs of each station. We have included detailed equipment checklists for each learning activity in the syllabus to help with learning preparation and set-up.

Additionally, the results of our curriculum's evaluation have important limitations to address. First, the findings were limited to the common biases in survey methodology and as such can only assess a learner's self-reported perceptions of comfort with pediatric emergency airway management before and after the curriculum.[38] While the results were certainly encouraging data to review, there were obvious limits to the validity and generalizability of the findings. Further, our results were limited by the fact that we were unable to query all of our target learners who participated, but did note much improved survey response rates once we transitioned to a mobile friendly electronic evaluation (37% vs. 71%).

We opted to not collect competency-based assessments through the scoring, collection, and analysis of the intubation teaching rubrics (Appendix D) used in the deliberate practice stations or during the simulations (stations 2-4). Instead, we promoted the use of these rubrics to help faculty deliver standardized teaching points. Similarly, we also did not analyze the chronometric data employed in station 2 as, again, we used this assessment technique to help learners think about how they could best prepare for advanced airway management in their real work environment over emphasizing the importance of beating their own time. Further, these teaching rubrics were inconsistently used by varying facilitators.

Finally, measuring provider behavior changes in the real clinical setting and improvements in patient level outcomes such as future first pass intubation success rates before and after our curriculum were also not measured or analyzed. First, we did not have access to these data at the outside institutions we worked with and, second, as mentioned, a pediatric intubation is an exceedingly rare event thus powering a medical education intervention to change this outcome would be quite challenging.

Moving forward, we are likely to invite learning groups to engage in our curriculum in our simulation center to facilitate use of local faculty personnel, resources, and equipment. While this in-vitro experience may lose some ability to uncover important latent safety issues at participants' facilities, the ability to transport extensive amounts of equipment and improve recruitment of a consistent, local faculty likely outweighs the potential benefits we experienced during the in-situ implementation.[36,40,41] We plan to continue to run our curricula every other year with our pediatric emergency medicine fellows and the emergency medicine residency to balance the many competing educational demands our learners face. We are also in the process of organizing an opportunity for community hospital providers, paramedics, emergency medical services personnel, and critical care or emergency medicine bound pediatric residents to participate in the near future.

Copyright © 2020 Goldman et al. This is an open-access publication distributed under the terms of the Creative Commons Attribution license.

In sum, we are excited to share our multimodal, pediatric emergency airway management curriculum that was grounded in proven educational strategies and theories and has been developed and reviewed by both pediatric airway experts and leading medical educators in the field. Our curriculum has been well received by our local trainees and those with varying training and pediatric experiences amongst our regional medical community. We openly welcome future facilitators and participants feedback on our curriculum. We hope these discussions will cultivate future collaborations as we all work to address the universal medical educator's challenge of teaching and maintaining competency of rare but critical procedures.

## Appendices

A. Course Syllabus.docx
B. Station 1 Didactic Videos.pptx
C. Station 2 Needle Cricothyrotomy Cognitive Aid.pptx
D. Intubation Teaching Feedback Rubrics.docx
E. Station 3 Simulation.docx
F. Station 4 Simulation.docx
G. Curriculum Evaluation.docx

*All appendices are peer reviewed as integral parts of the Original Publication.*

**Michael P. Goldman, MD:** Assistant Professor, Departments of Pediatrics and Emergency Medicine, Yale University School of Medicine

**Ambika Bhatnagar, MBBS:** Resident in Pediatrics, Riley Hospital for Children, Indiana University Health

**Joshua Nagler, MD, MHPEd:** Associate Professor, Department of Pediatrics and Emergency Medicine, Boston Children's Hospital, Harvard Medical School

**Marc A. Auerbach, MD, MSc:** Associate Professor, Departments of Pediatrics and Emergency Medicine, Yale University School of Medicine

### Disclosures
None to report.

### Funding/Support
None to report.

### Ethical Approval
Reported as not applicable.

## References

1. Kovacs G. Procedural skills in medicine: linking theory to practice. *J Emerg MEd.* 1997;15(3):387-391. https://doi.org/10.1016/S0736-4679(97)00019-X
2. Grantcharov TP, Reznick RK. Teaching procedural skills. *BMJ*. 2008;336(7653):1129-1131. https://doi.org/10.1136/bmj.39517.686956.47
3. Craig SS, Auerbach M, Cheek JA, et al. Preferred learning modalities and practice for critical skills: a global survey of paediatric emergency medicine clinicians. *Emerg Med J*. 2018;36(5):273-280. https://doi.org/10.1136/emermed-2017-207384
4. Gausche-Hill M, Ely M, Schmuhl P, et al. A national assessment of pediatric readiness of emergency departments. *JAMA Pediatr*. 2015;169(6):527-534. https://doi.org/10.1001/jamapediatrics.2015.138
5. Remick K, Gausche-Hill M, Joseph MM, et al. Pediatric readiness in the emergency department. *Pediatrics*. 2018;142(5):e20182459. https://doi.org/10.1542/peds.2018-2459
6. Brown AM, Decker SL, Selck FW. *NCHS Data Brief, no. 192: Emergency Department Visits and Proximity to Patients' Residences, 2009-2010.* National Center for Health Statistics; 2015. https://www.cdc.gov/nchs/data/databriefs/db192.pdf
7. Goldman MP, Wong AH, Bhatnagar A, Emerson BL, Brown LL, Auerbach MA. Providers' perceptions of caring for pediatric patients in community hospital emergency departments: a mixed-methods analysis. *Acad Emerg Med*. 2018;25(12):1385-1395. https://doi.org/10.1111/acem.13509
8. Query LA, Olson KR, Meyer MT, Drendel AL. Minding the gap: a qualitative study of provider experience to optimize care for critically ill children in general emergency departments. *Acad Emerg Med*. 2018;26(7):803-813. https://doi.org/10.1111/acem.13624
9. Michelson KA, Lyons TW, Hudgins JD, et al. Use of a national database to assess pediatric emergency care across United States emergency departments. *Acad Emerg Med*. 2018;25(12):1355-1364. https://doi.org/10.1111/acem.13489
10. Michelson KA, Hudgins JD, Lyons TW, Monuteaux MC, Bachur RG, Finkelstein JA. Trends in capability of hospitals to provide definitive acute care for children: 2008 to 2016. *Pediatrics*. 2020;145(1):e20192203. https://doi.org/10.1542/peds.2019-2203
11. Auerbach M, Whitfill T, Gawel M, et al. Differences in the quality of pediatric resuscitative care across a spectrum of emergency departments. *JAMA Pediatr*. 2016;170(10):987-994. https://doi.org/10.1001/jamapediatrics.2016.1550
12. Ericsson KA. Deliberate practice and acquisition of expert performance: a general overview. *Acad Emerg Med*. 2008; 15(11):988-994. https://doi.org/10.1111/j.1553-2712.2008.00227.x

Copyright © 2020 Goldman et al. This is an open-access publication distributed under the terms of the Creative Commons Attribution license.

13. Pusic M, Pecaric M, Boutis K. How much practice is enough? Using learning curves to assess the deliberate practice of radiograph interpretation. *Acad Med*. 2011;86(6):731-736. https://doi.org/10.1097/ACM.0b013e3182178c3c

14. Sawyer T, White M, Zaveri P, et al. Learn, see, practice, prove, do, maintain: an evidence-based pedagogical framework for procedural skill training in medicine. *Acad Med*. 2015;90(8):1025-1033. https://doi.org/10.1097/ACM.0000000000000734

15. Cheng A, Lang TR, Starr SR, Pusic M, Cook DA. Technology-enhanced simulation and pediatric education: a meta-analysis. *Pediatrics*. 2014;133(5):e1313-e1323. https://doi.org/10.1542/peds.2013-2139

16. Kessler D, Pusic M, Chang TP, et al. Impact of just-in-time and just-in-place simulation on intern success with infant lumbar puncture. *Pediatrics*. 2015;135(5):e1237-e1246. https://doi.org/10.1542/peds.2014-1911

17. Pusic MV, Kessler D, Szyld D, Kalet A, Pecaric M, Boutis K. Experience curves as an organizing framework for deliberate practice in emergency medicine learning. *Acad Emerg Med*. 2012;19(12):1476-1480. https://doi.org/10.1111/acem.12043

18. Auerbach MA, White ML, Bhargava S, et al. Are graduating pediatric residents prepared to perform infant lumbar punctures? A multi-institutional descriptive study. *Pediatr Emerg Care*. 2018;34(2):116-120. https://doi.org/10.1097/PEC.0000000000000914

19. Nagler J, Nagler A, Bachur RG. Development and assessment of an advanced pediatric airway management curriculum with integrated intubation videos. *Pediatr Emerg Care*. 2017;33(4):239-244. https://doi.org/10.1097/PEC.0000000000000777

20. Green-Hopkins I, Werner H, Monuteaux MC, Nagler J. Using video-recorded laryngoscopy to evaluate laryngoscopic blade approach and adverse events in children. *Acad Emerg Med*. 2015;22(11):1283-1289. https://doi.org/10.1111/acem.12799

21. Issa N, Schuller M, Santacaterina S, et al. Applying multimedia design principles enhances learning in medical education. *Med Educ*. 2011;45(8):818-826. https://doi.org/10.1111/j.1365-2923.2011.03988.x

22. Pinsky LE, Wipf JE. A picture is worth a thousand words: practical use of videotape in teaching. *J Gen Intern Med*. 2000;15:805-810. https://doi.org/10.1046/j.1525-1497.2000.05129.x

23. Lefebvre C, Manthey D. Advanced airway management classroom course. *MedEdPORTAL*. 2014;10:9960. https://doi.org/10.15766/mep_2374-8265.9960

24. Lefebvre C, Manthey D, Glass C, et al. Basic airway management classroom course. *MedEdPORTAL*. 2014;10:9766. https://doi.org/10.15766/mep_2374-8265.9766

25. Craig SS, Auerbach M, Cheek JA, et al. Preferred learning modalities and practice for critical skills: a global survey of paediatric emergency medicine clinicians. *Emerg Med J*. 2019;36(5):273-280. https://doi.org/10.1136/emermed-2017-207384

26. Leung SK, Cruz AT, Macias CG, Sirbaugh PE, Patel B. Improving pediatric emergency care by implementing an eligible learner endotracheal intubation policy. *Pediatr Emerg Care*. 2016;32(4):205-209. https://doi.org/10.1097/PEC.0000000000000764

27. Grissom TE, Sappenfield J, Galvagno SM, Cherry SV, Chang Y-CC, Hu PF. Performance assessment in airway management training for nonanesthesiology trainees: an analysis of 4,282 airway procedures performed at a level-1 trauma center. *Anesthesiology*. 2014;120(1):185-195. https://doi.org/10.1097/ALN.0000000000000064

28. Whitfill T, Auerbach M, Scherzer DJ, Shi J, Xiang H, Stanley RM. Emergency care for children in the United States: epidemiology and trends over time. *J Emerg Med*. 2018;55(3):423-434. https://doi.org/10.1016/j.jemermed.2018.04.019

29. Abu-Sultaneh S, Whitfill T, Rowan CM, et al. Improving simulated pediatric airway management in community emergency departments using a collaborative program with a pediatric academic medical center. *Respir Care*. 2019;84(9):1073-1081. https://doi.org/10.4187/respcare.06750

30. Abulebda K, Lutfi R, Whitfill T, et al. A collaborative in-situ simulation-based pediatric readiness improvement program for community emergency departments. *Acad Emerg Med*. 2017;25(2):177-185. https://doi.org/10.1111/acem.13329

31. Johnston L, Sawyer T, Nishisaki A, et al. Neonatal intubation competency assessment tool: development and validation. *Acad Pediatr*. 2019;19(2):157-164. https://doi.org/10.1016/j.acap.2018.07.008

32. Okuda Y, Bryson EO, DeMaria S, et al. The utility of simulation in medical education: what is the evidence? *Mt Sinai J Med*. 2009;76(4):330-343. https://doi.org/10.1002/msj.20127

33. So HY, Chen PP, Wong GKC, Chan TTN. Simulation in medical education. *J R Coll Physicians Edinb*. 2019;49(1):52-57. https://doi.org/10.4997/JRCPE.2019.112

34. Delisle M, Ward MAR, Pradarelli JC, Panda N, Howard JD, Hannenberg AA. Comparing the learning effectiveness of healthcare simulation in the observer versus active role. *Simul Healthc*. 2019;14(5):318-322. https://doi.org/10.1097/SIH.0000000000000377

35. Pusic MV, Brydges R, Kessler D, Szyld D, Nachbar M, Kalet A. What's your best time? Chronometry in the learning of medical procedures. *Med Educ*. 2014;48(5):479-488. https://doi.org/10.1111/medu.12395

36. Stone KP, Patterson MD, Reid JR, Geis GL, Auerbach M. Simulation to improve patient safety in pediatric emergency medicine. *Clin Ped Emerg Med*. 2016;17(3):185-192. https://doi.org/10.1016/j.cpem.2016.05.008

Copyright © 2020 Goldman et al. This is an open-access publication distributed under the terms of the Creative Commons Attribution license.

37. Meinema JG, Buwalda N, van Etten-Jamaludin FS, Visser MRM, van Dijk N. Intervention descriptions in medical education: what can be improved? A systematic review checklist. *Acad Med*. 2019;94(2):281-290. https://doi.org/10.1097/ACM.0000000000002428

38. Skeff KM, Stratos GA, Bergen MR. Evaluation of a medical faculty development program: a comparison of traditional pre/post and retrospective pre/post self-assessment ratings. *Eval Health Prof*. 1992;15(3):350-366. https://doi.org/10.1177/016327879201500307

39. Sawyer T, Eppich W, Brett-Fleegler M, Grant V, Cheng A. More than one way to debrief: a critical review of healthcare simulation debriefing methods. *Simul Healthc*. 2016;11(3):209-217. https://doi.org/10.1097/SIH.0000000000000148

40. Auerbach M, Stone K, Patterson M. The role of simulation in improving patient safety. In: *Comprehensive Healthcare Simulation: Pediatrics*. Springer; 2016:55-65.

41. Auerbach M, Kessler DO, Patterson M. The use of in situ simulation to detect latent safety threats in paediatrics: a cross-sectional survey. *BMJ Simul Technol Enhanc Learn*. 2015;1(3):77-82. https://doi.org/10.1136/bmjstel-2015-000037

42. Cutrer WB, Miller B, Pusic MV, et al. Fostering the development of master adaptive learners: a conceptual model to guide skill acquisition in medical education. *Acad Med*. 2016;92(1):70-75. https://doi.org/10.1097/ACM.0000000000001323

43. Boutis K, Pecaric M, Seeto B, Pusic M. Using signal detection theory to model changes in serial learning of radiological image interpretation. *Adv Health Sci Educ* 2010;15(5):647-658. https://doi.org/10.1007/s10459-010-9225-8

44. Quasim I. Advanced airway management. In: *Core Topics in Critical Care Medicine*. Cambridge University Press, 2010:6-15. https://doi.org/10.1017/CBO9780511712289.004

45. Green-Hopkins I, Eisenberg M, Nagler J. Video laryngoscopy in the pediatric emergency department: advantages and approaches. *Clin Pediatr Emerg Med*. 2015;16(3):195-202. https://doi.org/10.1016/j.cpem.2015.08.004

46. Eisenberg MA, Green-Hopkins I, Werner H, Nagler J. Comparison between direct and video-assisted laryngoscopy for intubations in a pediatric emergency department. *Acad Emerg Med*. 2016;23(8):870-877. https://doi.org/10.1111/acem.13015

47. Arafeh JMR, Hansen S, Nichols A. Debriefing in simulated-based learning: facilitating a reflective discussion. *J Perinat Neonatal Nurs*. 2010;24(4):302-309. https://doi.org/10.1097/JPN.0b013e3181f6b5ec

48. Eppich W, Cheng A. Promoting excellence and reflective learning in simulation (PEARLS): development and rationale for a blended approach to health care simulation debriefing. *Simul Healthc*. 2015;10(2):106-115. https://doi.org/10.1097/SIH.0000000000000072


**Received:** July 19, 2019
**Accepted:** February 26, 2020
**Published:** September 4, 2020

Copyright © 2020 Goldman et al. This is an open-access publication distributed under the terms of the Creative Commons Attribution license.
